# Risk association and diagnostic value of body roundness index for cardiovascular-kidney-metabolic-related outcomes: a systematic review and meta-analysis

**DOI:** 10.3389/fendo.2026.1814762

**Published:** 2026-04-14

**Authors:** Hongjia Fu, Hui Liu, Yamei Han, Tie Wang, Jia Yu, Wei Liu, Zhen Liu, Xiuru Hu, Zhenyan Shen, Yunxia Gao

**Affiliations:** 1 Department of Nursing, Hebei Cangzhou Hospital of Integrated Traditional Chinese Medicine and Western Medicine, Cangzhou, Hebei, China; 2 Faculty of Nursing, Hebei University of Chinese Medicine, Shijiazhuang, Hebei, China; 3 Department of Gastrointestinal and Abdominal Wall Hernia Surgery II, Hebei Cangzhou Hospital of Integrated Traditional Chinese Medicine and Western Medicine, Cangzhou, Hebei, China; 4 Nursing Department, Tongji Hospital, School of Medicine, Tongji University, Shanghai, China; 5 Department of Radiation Oncology, Hebei Cangzhou Hospital of Integrated Traditional Chinese Medicine and Western Medicine, Cangzhou, Hebei, China; 6 Department of Endocrinology, Hebei Cangzhou Hospital of Integrated Traditional Chinese Medicine and Western Medicine, Cangzhou, Hebei, China

**Keywords:** anthropometric indices, body roundness index, cardiovascular-kidney-metabolic syndrome, diagnostic value, meta-analysis

## Abstract

**Background:**

The accumulation of visceral fat is a pivotal factor in the development and progression of Cardiovascular-Kidney-Metabolic (CKM) Syndrome. The early identification of high-risk individuals is crucial for delaying disease progression. The body roundness index (BRI) is a novel measures for assessing visceral fat, but its association with CKM-related outcomes lacks comprehensive evidence.

**Methods:**

A comprehensive literature search was conducted to identify observational studies that examined the association between BRI and CKM-related outcomes. The search was performed in PubMed, Web of Science, and Embase, and updated to July 7, 2025. Effect sizes were pooled using a random-effects model, with heterogeneity and publication bias evaluated. Additionally, a diagnostic meta-analysis was performed to assess the discriminatory ability of BRI for specific metabolic risk factors.

**Results:**

A total of 93 studies (involving 13 countries) were included. BRI was significantly associated with the risk of multiple CKM-related outcomes, but its strength varied by outcome and gender subgroup. For metabolic syndrome, BRI exhibited consistent risk associations in the overall population and gender subgroups, with good discriminatory ability in the diagnostic meta-analysis. However, its predictive ability for chronic kidney disease, cardiovascular disease, and mortality was relatively constrained.

**Conclusion:**

Within the CKM framework, a significant association between BRI and the risk of multiple CKM-related outcomes has been identified, with favorable risk assessment and discriminatory performance of BRI observed, especially for metabolic abnormalities. As a complementary tool to conventional anthropometric indices, BRI can provide incremental information to optimize the identification and risk stratification of CKM-related risks. Further definition of the long-term predictive value and clinical utility of BRI across diverse global populations is warranted through high-quality prospective cohort studies.

**Systematic review registration:**

https://www.crd.york.ac.uk/PROSPERO/view/, identifier CRD420251110973.

## 1 Introduction

Cardiovascular-Kidney-Metabolic (CKM) Syndrome is a systemic disorder describing the interconnected and progressive pathological processes linking metabolic abnormalities, renal impairment, and cardiovascular disease (CVD) (1). This syndrome originates from early metabolic dysregulation, subsequently involving the kidneys and cardiovascular system as the disease progresses, and ultimately progresses to endpoints such as cardiovascular events and mortality (2). Consequently, early risk identification and stratified management during the progression of CKM constitutes a pivotal strategy for delaying disease progression and reducing adverse outcomes and mortality.

At different stages of CKM, excess fat or dysfunctional adipose tissue represents a core feature of the early disease stage (Stage 1), typically assessed using indices like body mass index (BMI) and waist circumference (WC) (1, 2). However, with growing recognition of obesity heterogeneity, traditional anthropometric indices have limitations in reflecting body fat distribution characteristics (3). These conventional anthropometric indices are unable to distinguish between fat mass and lean mass, and the distribution of visceral adipose tissue cannot be accurately captured by these measures. Visceral fat accumulation is widely acknowledged as a core driver of metabolic dysfunction, with the onset and progression of CKM promoted by this pathological state via key pathophysiological processes including chronic inflammation, insulin resistance, lipotoxicity, and vascular endothelial dysfunction (4–6). Relevant expert consensus notes that using BMI as the sole metric to define obesity may lead to adverse outcomes, including overdiagnosis (7). Despite sharing an identical BMI, people can display substantial heterogeneity in their body fat patterning. This variation is not trivial, as it may have profound implications for an individual’s cardiometabolic risk profile (8).

The Body Roundness Index (BRI) was conceived to provide a more precise quantification of an individual’s body fat distribution. Its design prioritizes the assessment of abdominal and visceral fat accumulation relative to overall body composition (9, 10). This is achieved by quantifying the roundness of the human body shape. In recent years, BRI has been increasingly utilized in research fields such as metabolic diseases, Chronic kidney disease (CKD), and CVD. Existing evidence confirms a robust link between BRI and multiple cardio-renal metabolic outcomes. In certain populations, BRI has exhibited superior risk predictive ability compared to BMI (11–13). However, single disease entities or isolated organ systems have been the primary focus of most prior research. The associations between BRI and the risk of the full spectrum of CKM-related outcomes, as well as its corresponding diagnostic performance, have not yet been systematically and comprehensively evaluated.

A systematic review and meta-analysis were conducted to synthesize available evidence on the associations between BRI and metabolic, renal, cardiovascular, and mortality clinical endpoints. A diagnostic meta-analysis was performed to assess the discriminatory performance of BRI for identifying key metabolic risk factors. In addition, a comprehensive analysis using conventional anthropometric indices was conducted to evaluate the performance of BRI from both risk association and diagnostic accuracy perspectives. This study was designed to define the clinical utility of BRI for risk stratification of CKM-related outcomes, and to provide evidence-based reference for the application of anthropometric indices in CKM-focused risk assessment.

## 2 Methods

### 2.1 Materials

The present work complied with the PRISMA 2020 statement for systematic reviews and meta-analyses (14). The protocol for this systematic review is publicly recorded in the PROSPERO registry, with the unique identifier CRD420251110973.

### 2.2 Search strategy

We performed a structured search across PubMed, Web of Science, and Embase, covering all records up to July 7, 2025. In order to capture all relevant studies, the strategy was designed utilizing both standardized MeSH terms and free-text keyword. The search terms included “body roundness index” or “BRI”. The search strategy for each database is available in the Supplementary Materials. Following deduplication of all retrieved records, we conducted an initial screening based on titles and abstracts, with unclear records proceeding directly to full-text review. Eligible full texts were evaluated using predefined criteria. All screening steps were performed independently by two researchers (L.H. and F.H.J.), with consensus obtained through consultation with a third (H.Y.M.) in cases of disagreement. The search strategy omitted terms for other anthropometric measures because studies lacking BRI data would be filtered out during screening. However, for included studies that also reported conventional indices (e.g., BMI), relevant numerical data were collected for comparative analysis within this review. This comparison focused on assessing the risk association and predictive efficacy for CKM-related outcomes and mortality.

### 2.3 Study eligibility criteria

Inclusion criteria were as follows (1): Outcomes: Studies reporting risks of CKM-related outcomes and mortality (2); Anthropometric index: BRI (3); Participants: Adults aged ≥18 years (4); Purpose: To assess the risk association between BRI and the above outcomes (5); Study design: Cohort or cross-sectional studies (6); Language: English.

Studies were excluded if they met any of the following conditions (1): Failure to report health outcomes associated with BRI (2); Letters, editorials, reviews, conference abstracts, guidelines, or research/review protocols (3); Unavailable full text (4); Non-peer-reviewed (5); Availability of fewer than two studies per specific outcome (6); Data formats or statistical models incompatible with other studies, and unable to be converted into a unified format for meta-analysis.

### 2.4 Data extraction

We employed a standardized form for data extraction across all included studies. The extracted information encompassed both methodological and outcome-related items. Methodological details consisted of the first author’s name, year of publication, country, study design, data source, participant number, age range, proportion of female participants, disease status, follow-up period, confounder adjustment, statistical approaches, and the method for assessing BRI. Outcome data comprised measures such as odds ratios (OR), risk ratios (RR), hazard ratios (HR), the area under the receiver operating characteristic curve (AUC), and the predictive estimates for anthropometric indices including BRI, BMI, and WC etc.

### 2.5 Quality assessment

The National Institutes of Health (NIH) quality assessment tool for studies of diagnostic accuracy was applied to evaluate every study incorporated in this review (15).The tool comprises 14 evaluation items: a “Yes” response is awarded 1 point, while “No,” “Not Applicable,” “Not Reported,” or “Unclear” responses are awarded 0 points. The final score is defined as the sum of all item scores, with possible values ranging 0 to 14.

For studies included in the diagnostic meta-analysis, the Quality Assessment of Diagnostic Accuracy Studies-2 (QUADAS-2) tool was additionally employed. An evaluation covering the domains of index test, reference standard, patient selection, and participant flow/timing was conducted to determine the risk of bias and applicability of each study, using Review Manager 5.4 (16).

Two researchers (F.H.J. and L.H.) independently appraised the quality of each study, with a third (H.Y.M.) resolving any disagreements. Although quality indicates the evidence’s strength, it was not a basis for inclusion.

### 2.6 Statistical analysis

A meta-analysis was performed using StataMP 18.0 to assess the risk association between each included anthropometric index and CKM-related outcomes. A pooled effect size was estimated only when at least two studies employed the same outcome measure. Effect sizes for each anthropometric index were standardized per 1 standard deviation (SD) increment. For each anthropometric index, we first calculated the logarithm of the relative risk (log(RR)), hazard ratio (log(HR)), or odds ratio (log(OR)) corresponding to each SD increment of the index reported in the study. The result was then exponentiated to derive the corresponding RR, HR, or OR for a one-SD increase in the anthropometric index. During the synthesis of effect sizes, ORs and HRs were reliably approximated to RRs for studies with a low event rate (< 10%). For studies with an event rate of 10% or higher, OR values were transformed into RRs using a standardized conversion procedure, to ensure consistency and comparability across all pooled effect estimates. The conversion utilized the formula:


$$RR=\frac{OR}{\left(1-P0)+(P0\times OR\right)}$$

Following these principles, eligible HR, OR, and RR values were combined, with results uniformly reported in RR format. To verify the robustness of results, sensitivity analyses employing the “leave-one-out” method were conducted by excluding individual studies from the risk association meta-analysis. Publication bias was also quantitatively evaluated using Egger’s test, a commonly employed statistical method to assess funnel plot asymmetry (17).

Furthermore, to evaluate the diagnostic performance of each anthropometric index for CKM-related metabolic risk outcomes, a diagnostic meta-analysis was subsequently performed using the midas module in StataMP 18.0. This module is designed to calculate pooled sensitivity, specificity, positive likelihood ratio (PLR), negative likelihood ratio (NLR), diagnostic odds ratio (dOR), and area under the curve (AUC), with 95% confidence intervals provided for each metric. Additionally, the metadta package was utilized to plot summary receiver operating characteristic (SROC) curves, facilitating the evaluation of diagnostic accuracy of anthropometric indices (18). Overall discriminative performance was quantified using AUC-ROC. This package also supports graphical representation of SROC curves for different subgroups, enabling intuitive comparison of diagnostic performance across groups (19). anthropometric indices were classified based on the AUC of the SROC curve, using thresholds defined by Swets (20): ≤0.5 (no discriminatory ability), >0.5 to ≤0.7 (low discriminatory ability), >0.7 to ≤0.9 (good discriminatory ability), and 1 (perfect test). In diagnostic meta-analyses, potential publication bias was identified using the Deek’s Funnel Plot Asymmetry Test.

We assessed heterogeneity using Cochran’s Q test and the Higgins I² statistic. In accordance with Cochrane recommendations, I² values were interpreted as follows: 0% (negligible), 25% (low), 50% (moderate), and 75% (high). An I² value >75% indicates considerable heterogeneity (21). Given the anticipated between-study heterogeneity, effect sizes were synthesized using a random-effects model (22). A *p<* 0.05 (two-sided) was considered statistically significant for all analyses. In Deek’s test for funnel plot asymmetry, a threshold of *p* < 0.10 was applied to evaluate potential publication bias.

## 3 Results

### 3.1 Study selection

**Figure 1:**
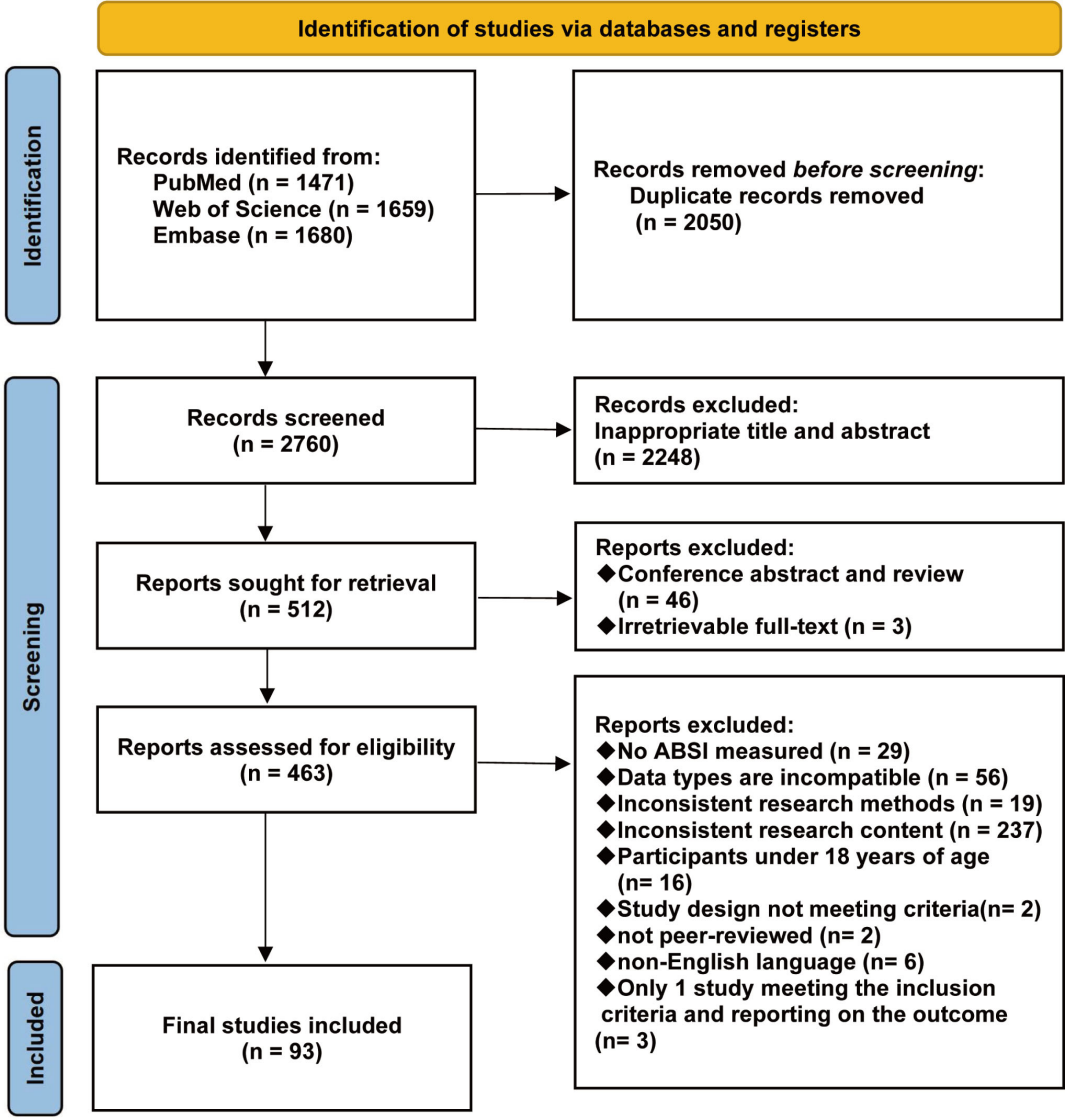
Study selection flow chart.

From the 4,810 records initially retrieved, 2,050 duplicates were removed. Screening of 2,760 titles and abstracts excluded 2,248 records. After further excluding 46 conference abstracts, guidelines, reviews, and 3 unavailable full texts, 463 articles entered full-text review, of which 370 were excluded based on the eligibility criteria. Specific reasons for exclusion were as follows: no BRI assessment (n = 29); incompatible data types (n = 56); inconsistent study methodologies (n = 19); study participants aged< 18 years (n = 16); non-cross-sectional or non-cohort study design (n = 2); non-peer-reviewed publications (n = 2); non-English publications (n = 6); only one relevant study available for three disease categories (n = 3); and were irrelevant to the study objectives (n = 237). A total of 93 eligible studies were included in the final analysis (23–115). The detailed screening process is presented in **Figure 1**.

### 3.2 Basic characteristics of the selected studies

**Figure 2:**
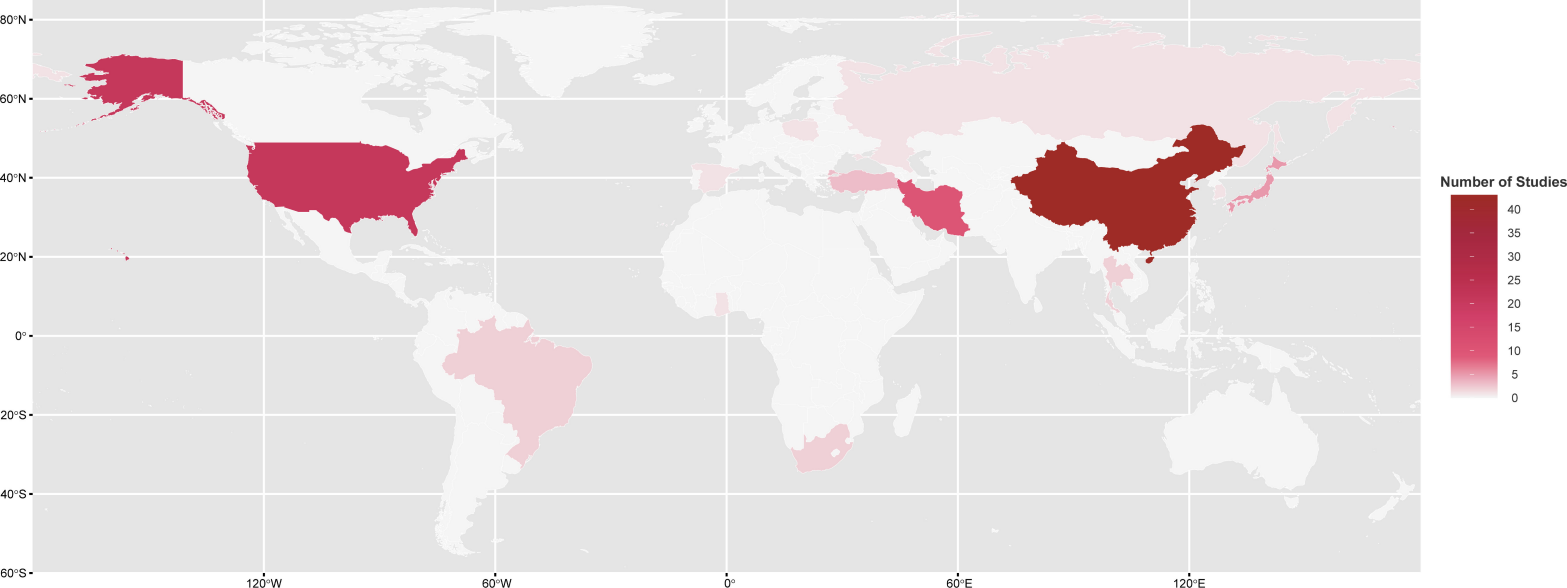
Geographic Distribution of Included Studies.

The baseline characteristics of 93 included studies are detailed in **Supplementary Table 1**. These studies covered 13 countries in total, predominantly China (n = 43) and the United States (n = 21), followed by Iran (n = 10) and Japan (n = 5), with the remaining studies distributed across other countries (**Figure 2**). Study designs included 55 cross-sectional studies (23, 27–31, 33, 35, 37–42, 44, 45, 50, 54–60, 62, 64–66, 71–73, 76, 80, 82, 83, 85–87, 90, 92, 93, 96, 99, 101–103, 105–111, 113, 114) and 38 cohort studies (32 prospective and 6 retrospective). The median sample size was 7,651 participants, encompassing a range from 128 to 121,888 across the studies.

In terms of study populations, 73 focused on the general population, and the remaining 20 (25, 28, 33, 39, 42, 46, 51–54, 62, 63, 67, 68, 80, 88, 98, 103, 104, 110) targeted specific disease cohorts (e.g., cardiometabolic syndrome, diabetes, and osteoporosis).

Among the 38 cohort studies, 36 reported follow-up data spanning 2.8 to 27 years, with a median follow-up period of 6.72 years. For covariate adjustment, 80 studies adjusted for major confounding factors, including demographic characteristics and health-related factors (e.g., smoking history, alcohol use history, family history, and medication use). Additionally, 6 studies (57, 80, 86, 92, 101, 110) did not adjust for covariates, 3 (55, 70, 96) adjusted only for age, another 3 (65, 73, 108) adjusted only for age and sex, and 1 adjusted only for occupational risk factors (106).

Primary outcomes and predictive anthropometric indices across all studies are presented in **Supplementary Table 2**. BMI was included in all 93 studies, while WC was reported in 69 studies, and waist-to-height ratio (WHtR) was reported in 51 studies. Study outcomes included metabolic diseases (n = 67) (23–25, 27, 28, 30–32, 34, 35, 40, 44, 45, 47–50, 53–66, 69, 72–74, 77, 78, 80–87, 89–97, 100–103, 105–109, 111, 113–115), CVDs (n = 14) (26, 36, 37, 40, 42, 47, 52, 67, 68, 71, 75, 76, 110, 112), CKD (n = 7) (29, 33, 39, 41, 43, 79, 99), and mortality (n = 7) (38, 46, 51, 70, 88, 98, 104), with some studies reporting multiple outcomes.

### 3.3 Meta-analysis of risk associations

#### 3.3.1 Metabolic risk factors

**Figure 3:**
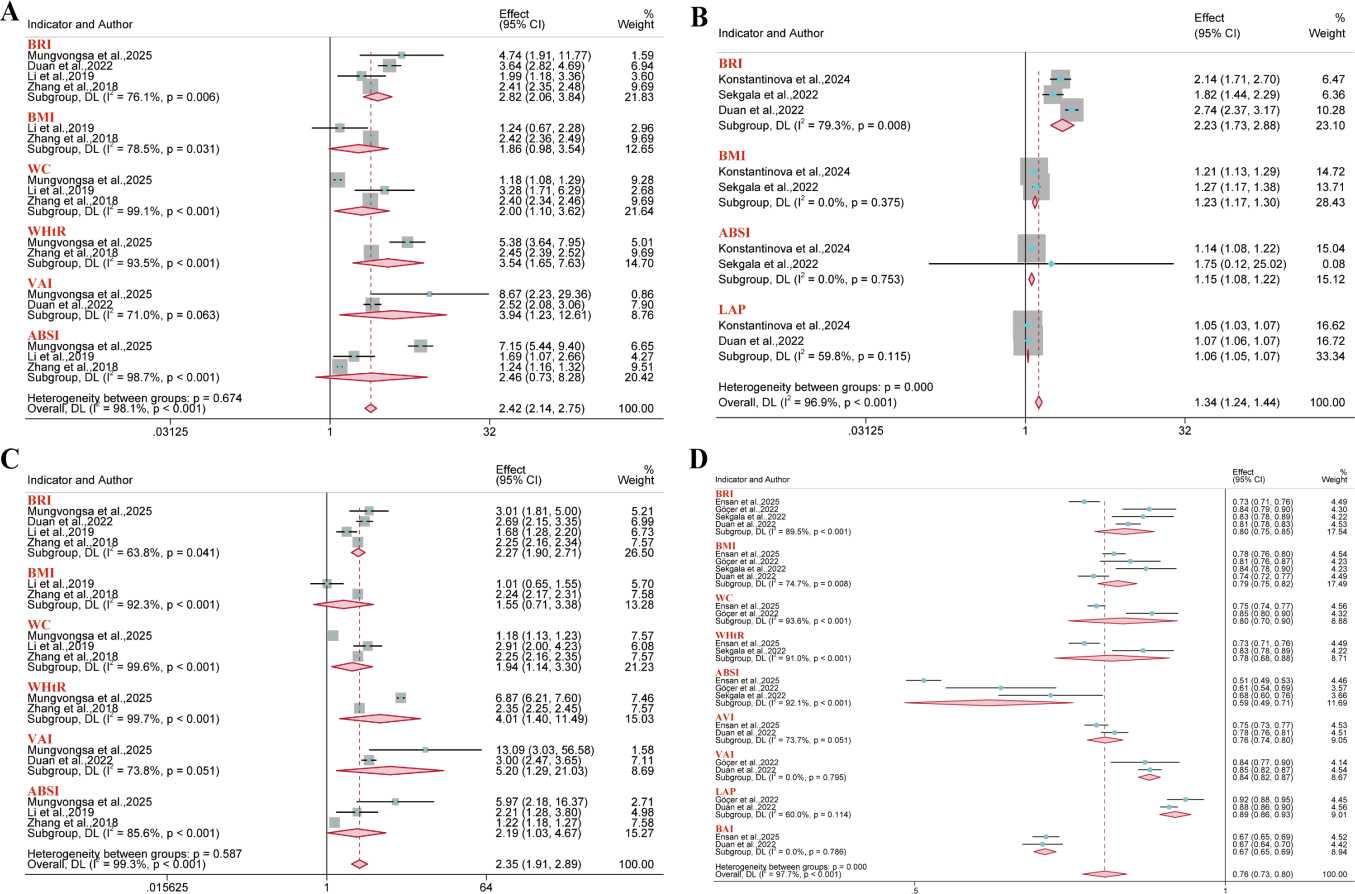
Meta-analysis of the links between multiple anthropometric indices and metabolic syndrome risk. **(A–C)** Forest plots of ORs in the overall, male, and female populations; **(D)** Pooled AUC values from the overall sample quantified the predictive performance of different anthropometric indices for metabolic syndrome.

**Figure 4:**
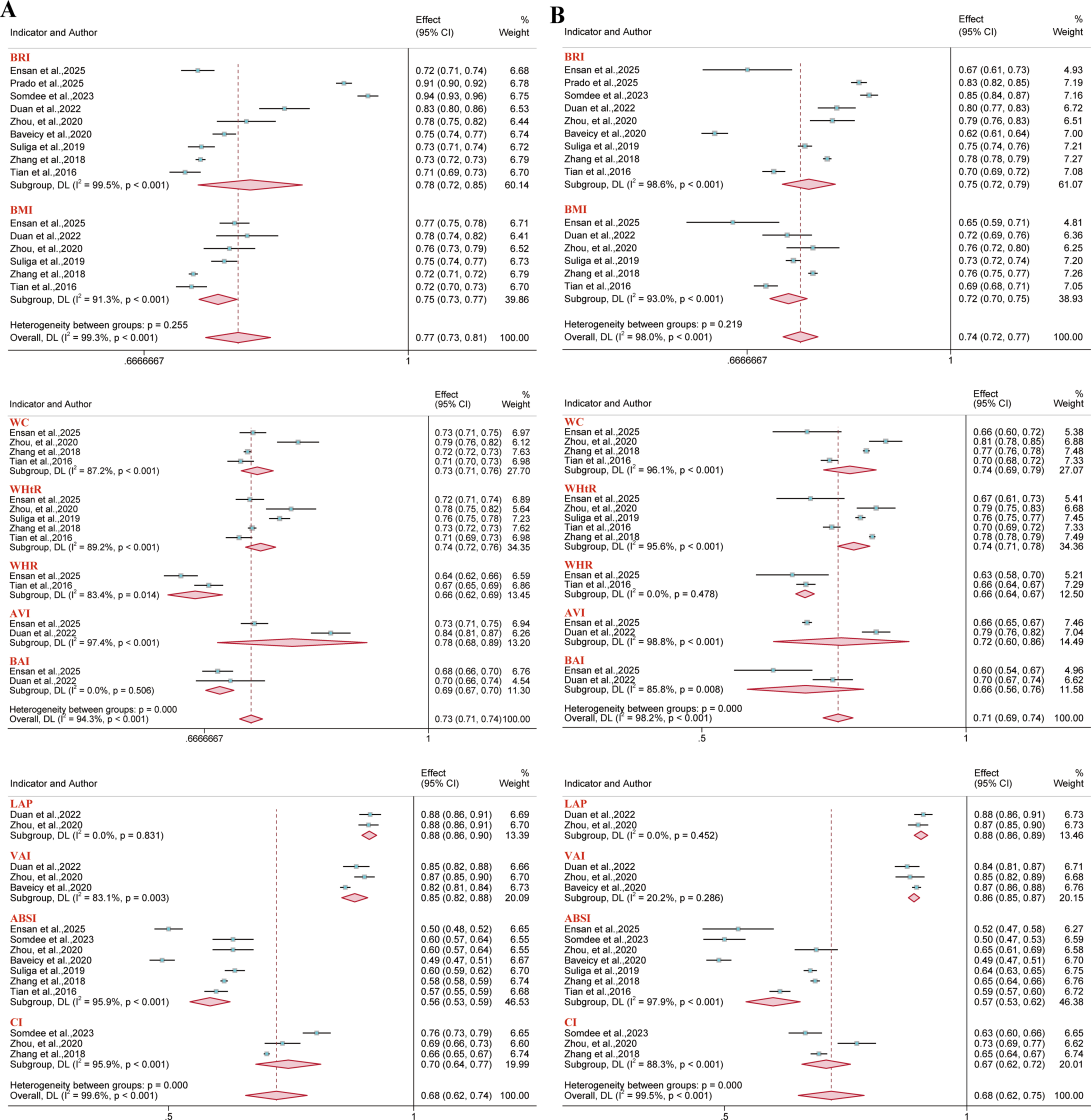
Pooled AUC values of different anthropometric indices for predicting metabolic syndrome risk. **(A)** Male population; **(B)** Female population.

A total of 14 reports were included for metabolic syndrome outcomes. Meta-analysis results demonstrated a significant positive association between BRI and metabolic syndrome risk in the overall population (OR = 2.23, 95% CI: 1.73–2.88; I² = 79.3%), males (OR = 2.82, 95% CI: 2.06–3.84; I² = 76.1%), and females (OR = 2.27, 95% CI: 1.90–2.71; I² = 63.8%). However, no significant association was identified between BMI and metabolic syndrome risk in either gender subgroup (**Figure 3**). Regarding the predictive performance of BRI, it exhibited good discriminative ability in the overall population (AUC = 0.80, 95% CI: 0.75–0.85; I² = 89.5%), males (AUC = 0.78, 95% CI: 0.72–0.85; I² = 99.5%), and females (AUC = 0.75, 95% CI: 0.72–0.79; I² = 98.6%). Furthermore, anthropometric indices like WC and WHtR were significantly associated with the risk of metabolic syndrome, indicating certain predictive value. Conversely, A Body Shape Index (ABSI) demonstrated marginal predictive value in both the overall and gender subgroups (**Figure 4**).

**Figure 5:**
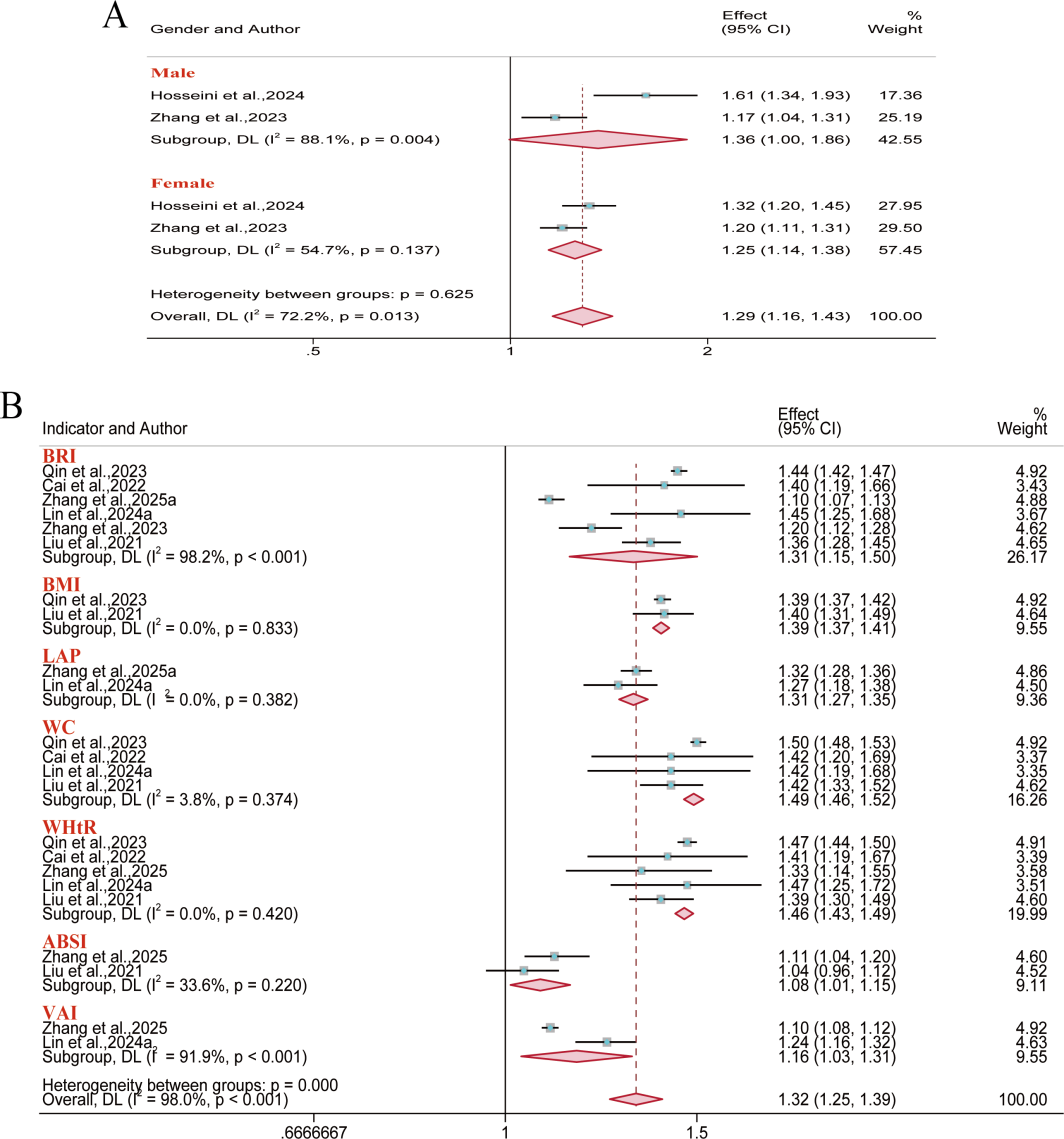
Meta-analysis of the association between different anthropometric indices and diabetes risk. **(A)** Gender subgroups; **(B)** Overall population.

**Figure 6:**
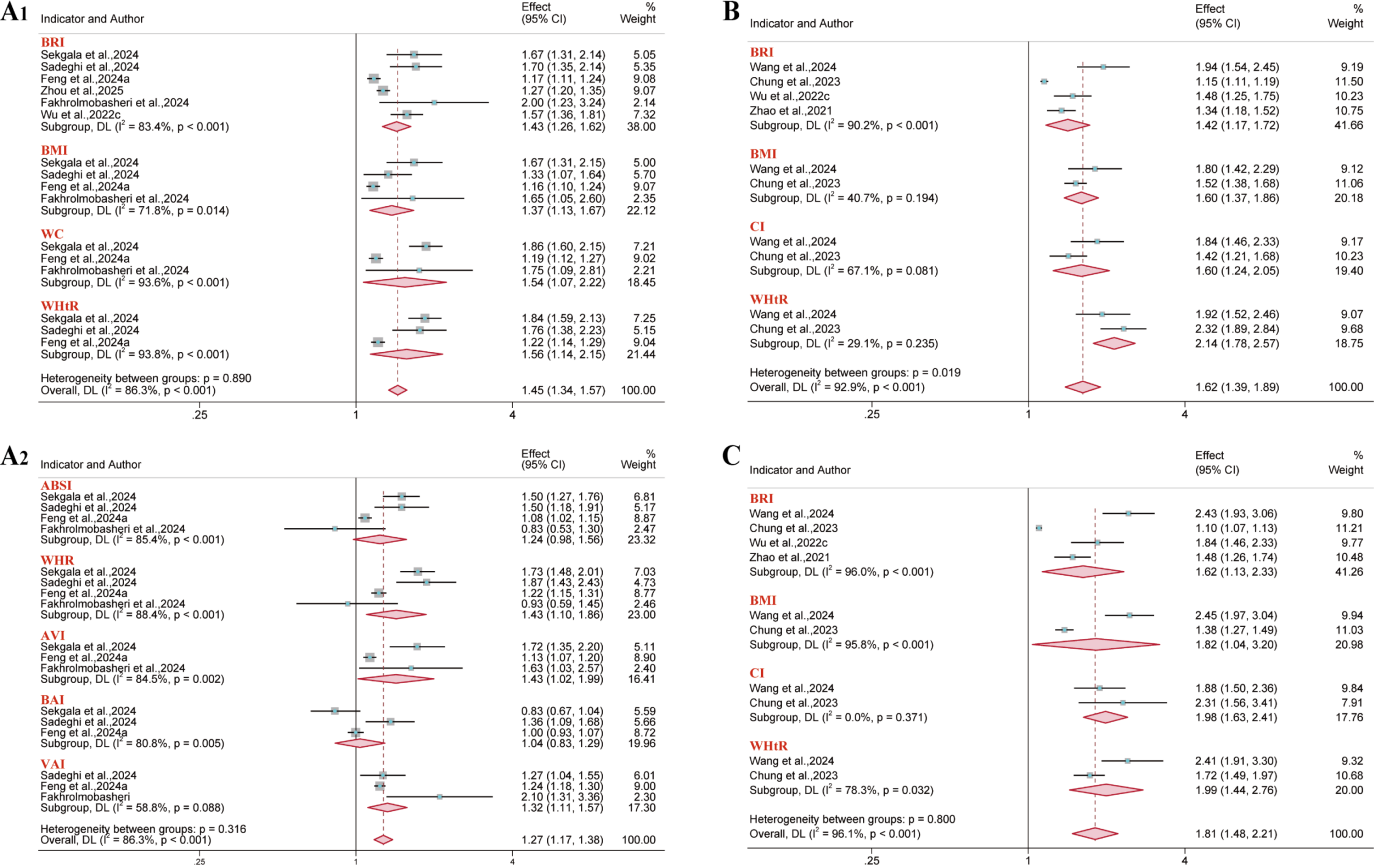
Meta-analysis of the association between different anthropometric indices and type 2 diabetes risk. **(A1–A2)** Overall population; **(B)** Males; **(C)** Females.

The analysis showed that outcomes related to diabetes and type 2 diabetes were reported in 12 and 11 reports, respectively. Results demonstrated that BRI correlated with heightened risks observed for diabetes (OR = 1.31, 95% CI: 1.15–1.50; I² = 98.2%) and type 2 diabetes (OR = 1.43, 95% CI: 1.26–1.62; I² = 83.4%) in the overall population. Our analysis indicated that the Body Adiposity Index (BAI) was not significantly associated with an elevated risk of type 2 diabetes across overall population (**Figures 5**, **6**). Regarding the predictive performance of the indices, BRI exhibited favorable predictive value in the female diabetes subgroup (AUC = 0.71, 95% CI: 0.70–0.73; I² = 11.7%), while the remaining indices demonstrated limited predictive value across all subgroups (AUC< 0.70) (**Supplementary Figure 1**-**S3**).

Four reports were included for dyslipidemia outcomes. The predictive value of all anthropometric indices proved to be limited (AUC< 0.70) in both the overall population and gender subgroups (**Supplementary Figure 4**-**S5**).

**Figure 7:**
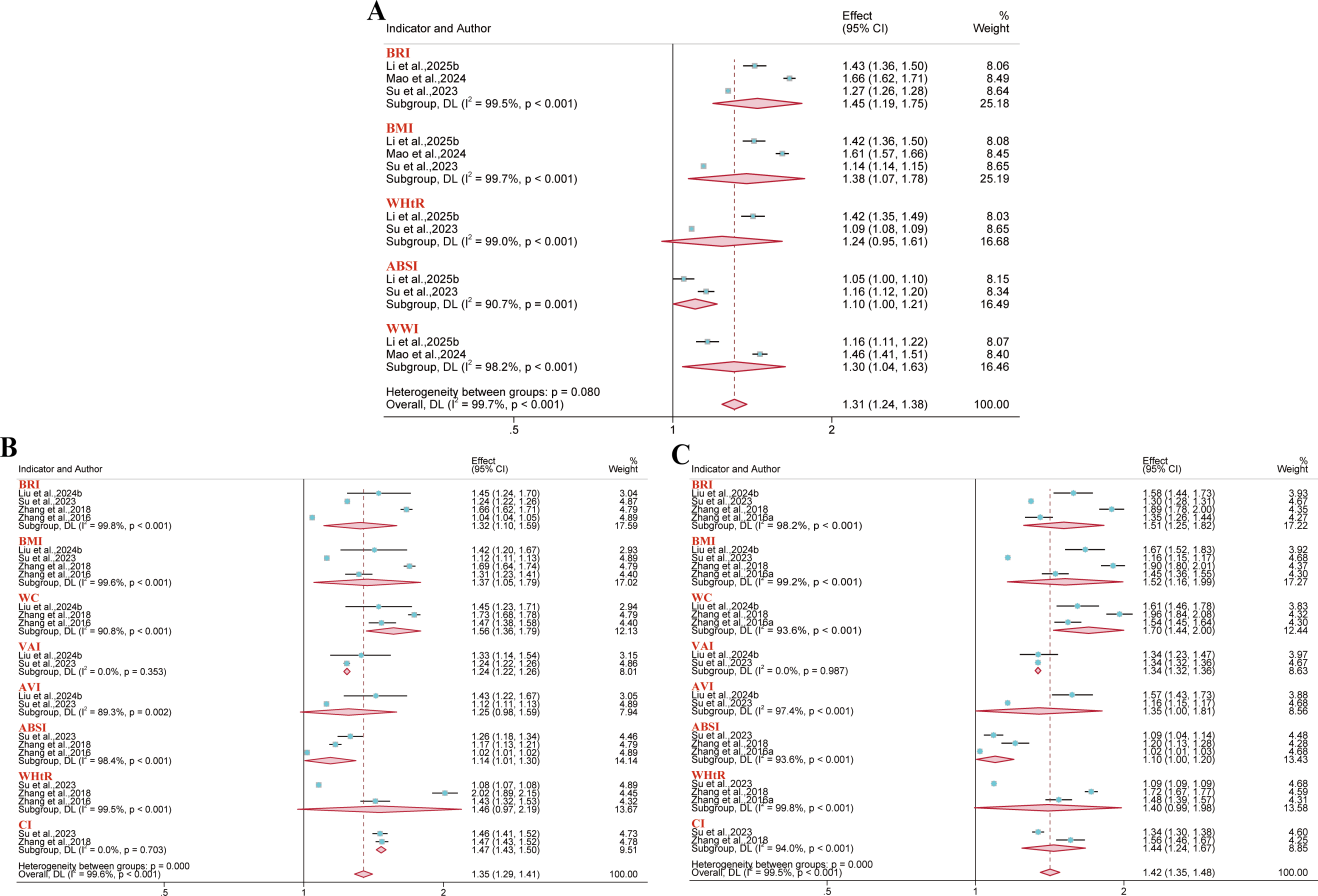
Meta-analysis of the association between different anthropometric indices and hyperuricemia risk. **(A)** Overall population; **(B)** Male population; **(C)** Female population.

**Figure 8:**
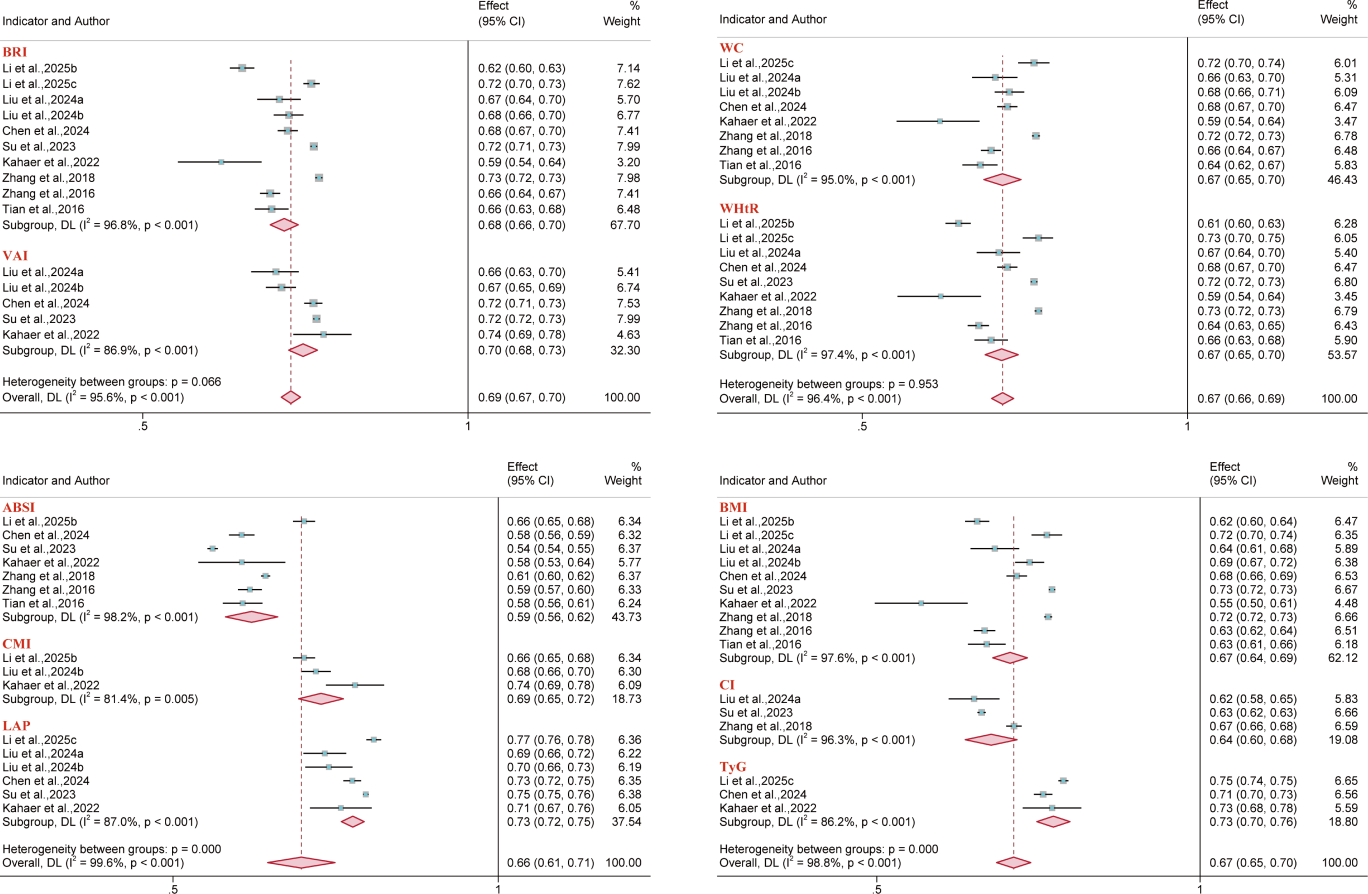
Predictive Performance of Anthropometric Indices for Hyperuricemia in Females: Pooled AUC Values.

For hyperuricemia outcomes, 13 reports were incorporated into the analysis. Results demonstrated a consistent positive association between BRI and hyperuricemia risk in the overall population (OR = 1.45, 95% CI: 1.19–1.75; I² = 99.5%). This association remained significant in both sex-stratified analyses: among males (OR = 1.32, 95% CI: 1.10–1.59; I² = 99.8%) and females (OR = 1.51, 95% CI: 1.25–1.82; I² = 98.2%). However, neither WHtR (in the overall population or gender subgroup) nor the abdominal volume index (AVI) (in males specifically) showed a significant association with hyperuricemia risk (**Figure 7**). Among females, both the lipid accumulation product (LAP) (AUC = 0.73; 95% CI: 0.72–0.75; I² = 87.0%) and the Triglyceride-glucose (TyG) index (AUC = 0.73; 95% CI: 0.70–0.76; I² = 86.2%) showed satisfactory predictive value (**Figure 8**). Conversely, the remaining anthropometric indices demonstrated limited predictive value (AUC< 0.70) (**Supplementary Figure 6**-**S7**).

**Figure 9:**
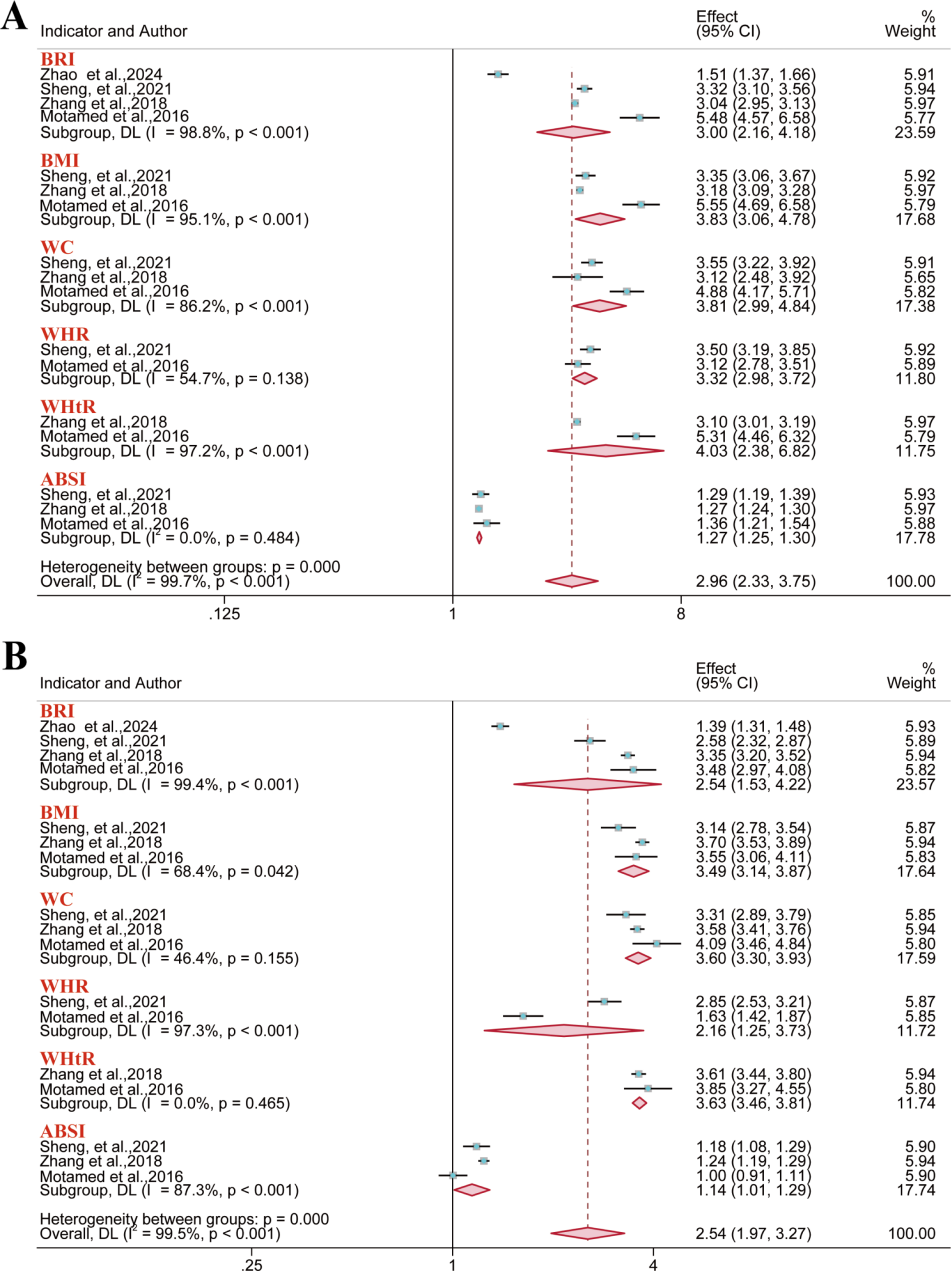
Meta-analysis examining anthropometric indices and the risk of NAFLD. **(A)** Male population; **(B)** Female population.

The outcomes of non-alcoholic fatty liver disease (NAFLD) and metabolic dysfunction-associated fatty liver disease (MAFLD) were included in five and four reports, respectively. In analyses stratified by gender, results demonstrated consistent positive associations with NAFLD risk for each anthropometric index (**Figure 9**). Moreover, regarding the predictive performance, ABSI demonstrated minimal predictive value in the NAFLD gender subgroups and the overall MAFLD population (AUC< 0.60). Visceral adiposity index (VAI) showed moderate predictive value in the NAFLD male subgroup (AUC = 0.69; 95% CI: 0.57–0.83; I² = 96.7%). The remaining anthropometric indices demonstrated good predictive value across all subgroups (AUC > 0.70) (**Supplementary Figure 8**-**S9**).

**Figure 10:**
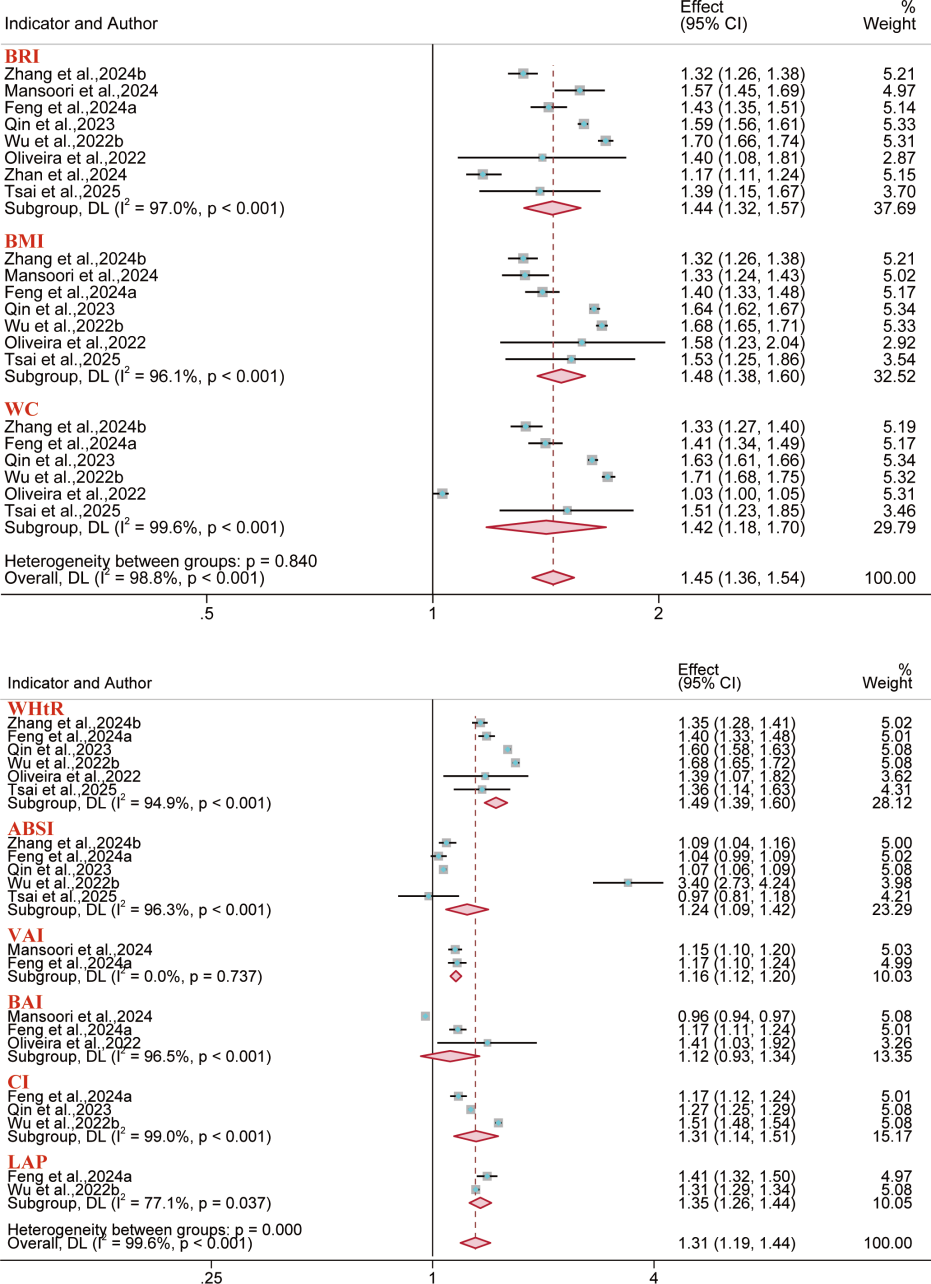
Meta-analysis examining anthropometric indices and the risk of hypertension in the overall population.

**Figure 11:**
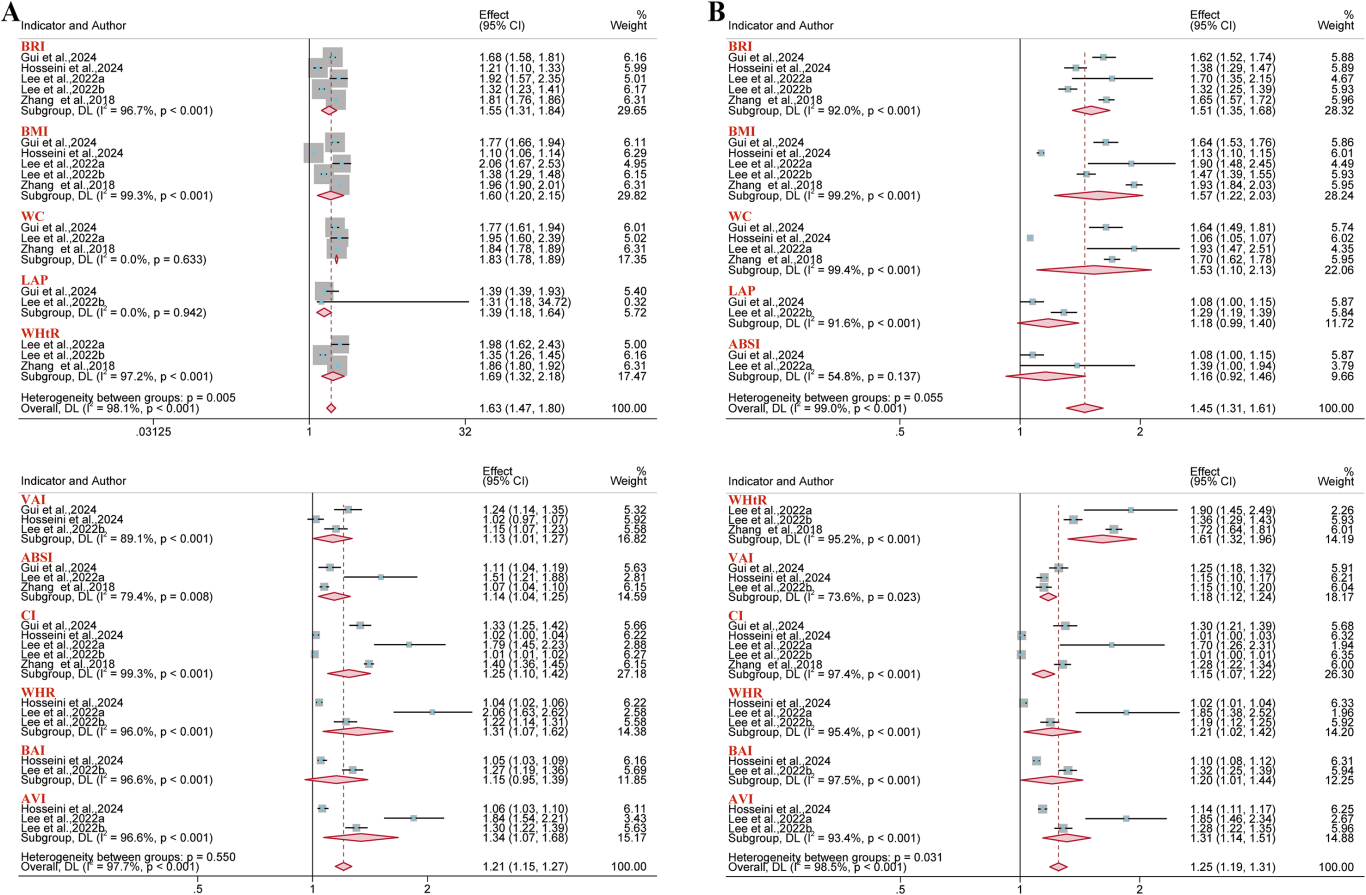
Meta-analysis examining anthropometric indices and the risk of hypertension. **(A)** Male population; **(B)** Female population.

Fifteen reports were included for hypertension outcomes. Results demonstrated that BRI was associated with an increased risk of hypertension in the overall population (OR = 1.44, 95% CI: 1.32–1.57; I² = 97.0%), males (OR = 1.55, 95% CI: 1.31–1.84; I² = 96.7%), and females (OR = 1.51, 95% CI: 1.35–1.68; I² = 92.0%) (**Figures 10**, **11**). However, BAI demonstrated no significant association with hypertension risk in the overall population or male subgroup, while LAP and ABSI also demonstrated no significant association in the female subgroup. The collective assessment of multiple anthropometric indices indicated a restricted predictive value for hypertension risk (AUC< 0.70) (**Supplementary Figure 10**-**S11**).

#### 3.3.2 CKD

**Figure 12:**
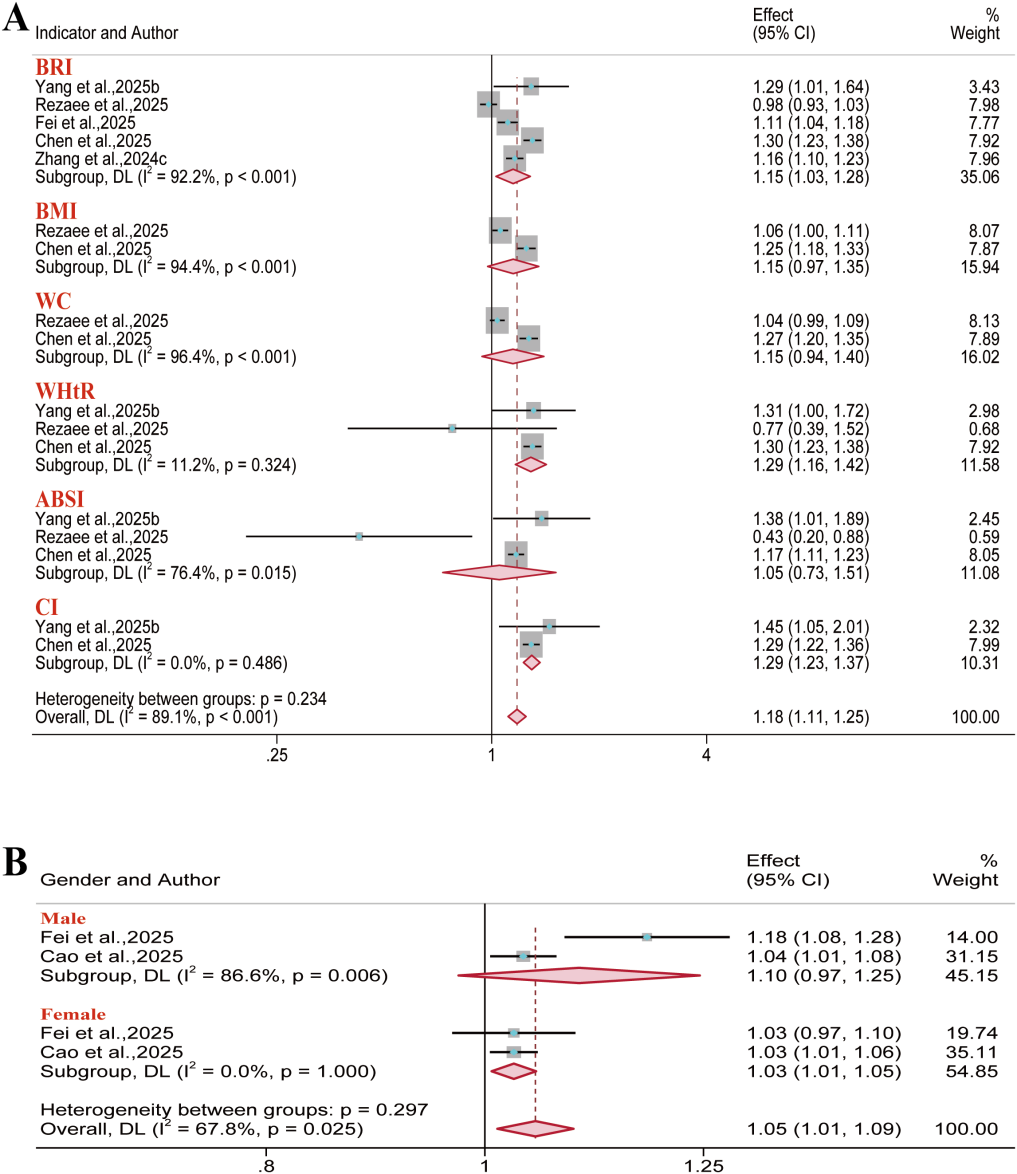
Meta-analysis of the association between different anthropometric indices and chronic kidney disease risk. **(A)** Overall population; **(B)** Gender subgroups.

A total of seven reports were included for chronic kidney disease outcomes. The meta-analysis indicated a significant positive association between BRI and CKD risk in the overall population (OR = 1.15, 95% CI: 1.03–1.28; I² = 92.2%) and the female subgroup (OR = 1.03, 95% CI: 1.01–1.05; I² = 0). However, no significant association was identified in the male subgroup (**Figure 12**). Furthermore, BMI, WC, and ABSI demonstrated no significant association with CKD risk in the overall population. Regarding predictive performance, all anthropometric indices exhibited limited discriminatory ability for CKD outcomes (AUC< 0.70) (**Supplementary Figure 12**).

#### 3.3.3 CVDs

**Figure 13:**
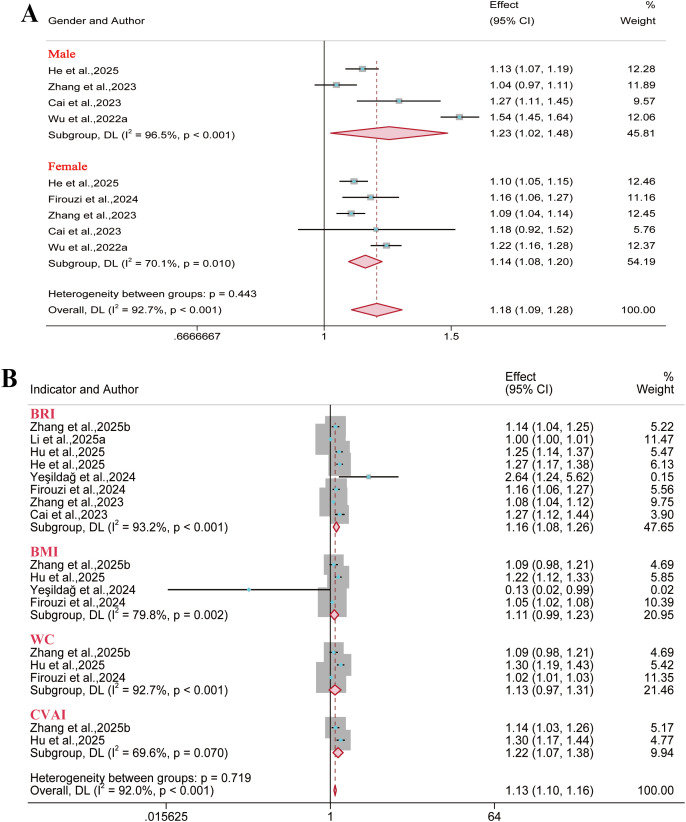
Meta-analysis examined the links between different anthropometric indices and CVD risk. **(A)** Gender subgroups; **(B)** Overall population.

**Figure 14:**
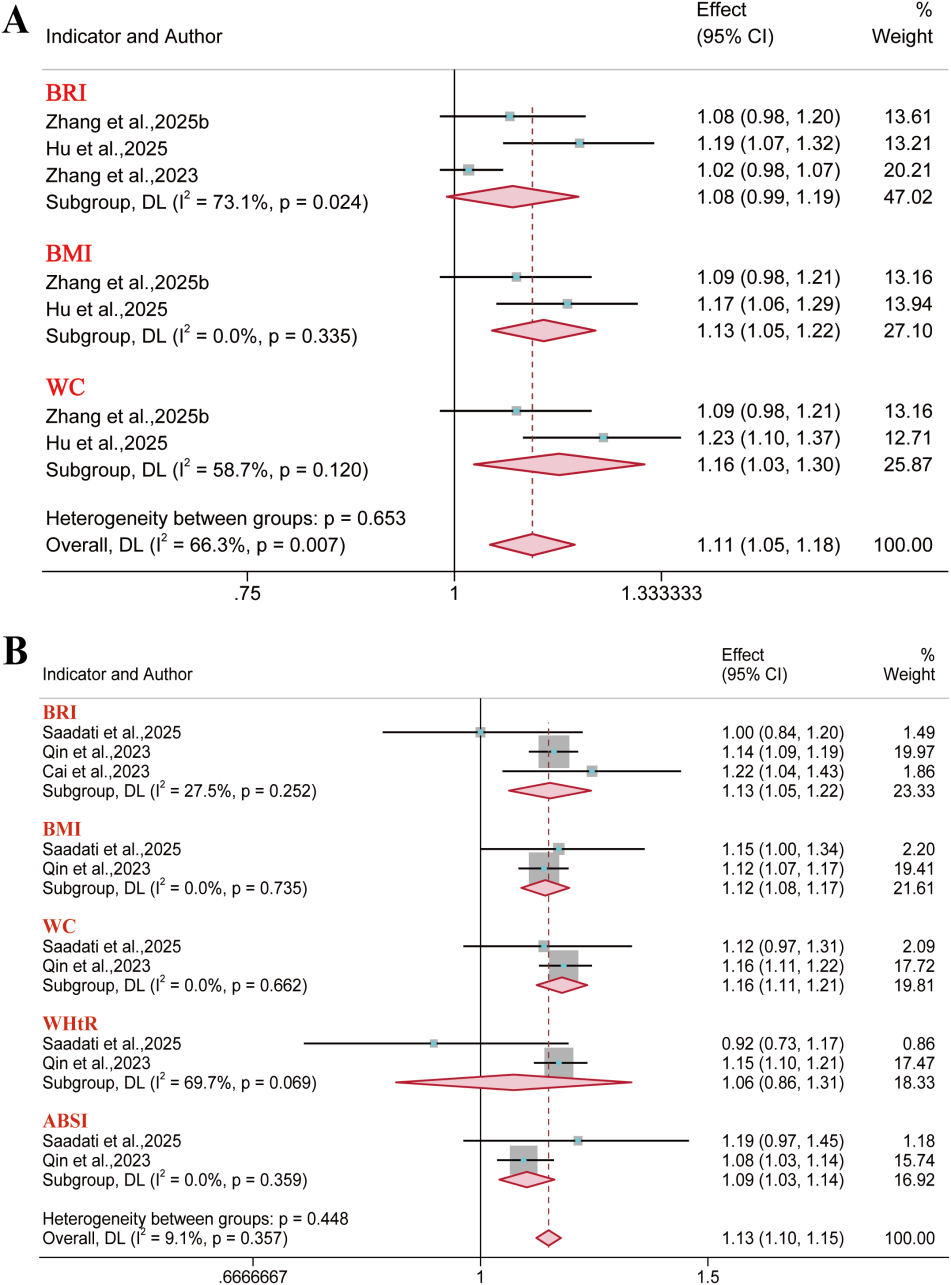
Meta-analysis examined the links between different anthropometric indices and CVD risk. **(A)** Heart disease; **(B)** Coronary heart disease.

**Figure 15:**
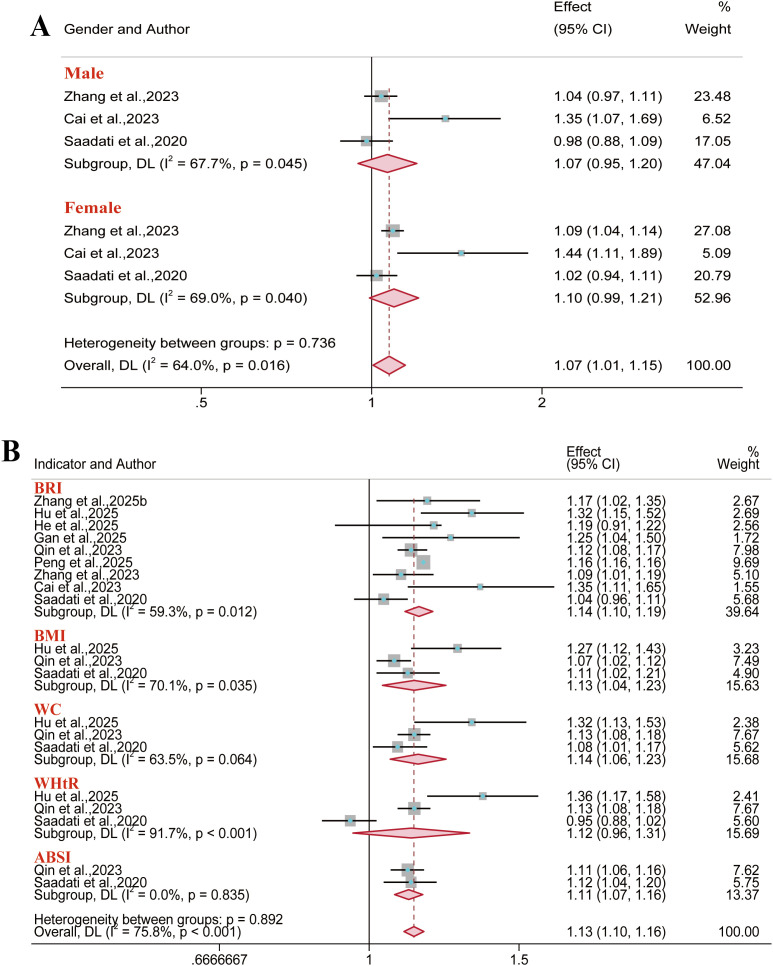
Meta-analysis examined the links between different anthropometric indices and stroke risk. **(A)** Gender subgroups; **(B)** Overall population.

Nine reports were included for CVD outcomes, while three, three, and nine reports covered its major subtypes (coronary heart disease, heart disease, and stroke), respectively. Results demonstrated that in the overall population, BRI was associated with an increased risk of CVD (OR = 1.16, 95% CI: 1.08–1.26; I² = 93.2%), coronary heart disease (OR = 1.13, 95% CI: 1.05–1.22; I² = 27.5%), and stroke (OR = 1.14, 95% CI: 1.10–1.19; I² = 59.3%). However, the analysis did not reveal a significant link to the risk of heart disease (OR = 1.08, 95% CI: 0.99–1.19; I² = 73.1%). Furthermore, in the overall population, no significant association was found between either BMI or WC and overall CVD risk. Similarly, WHtR showed no significant association with the risks of either stroke or coronary heart disease. (**Figure 13**–**15**).

#### 3.3.4 Mortality

**Figure 16:**
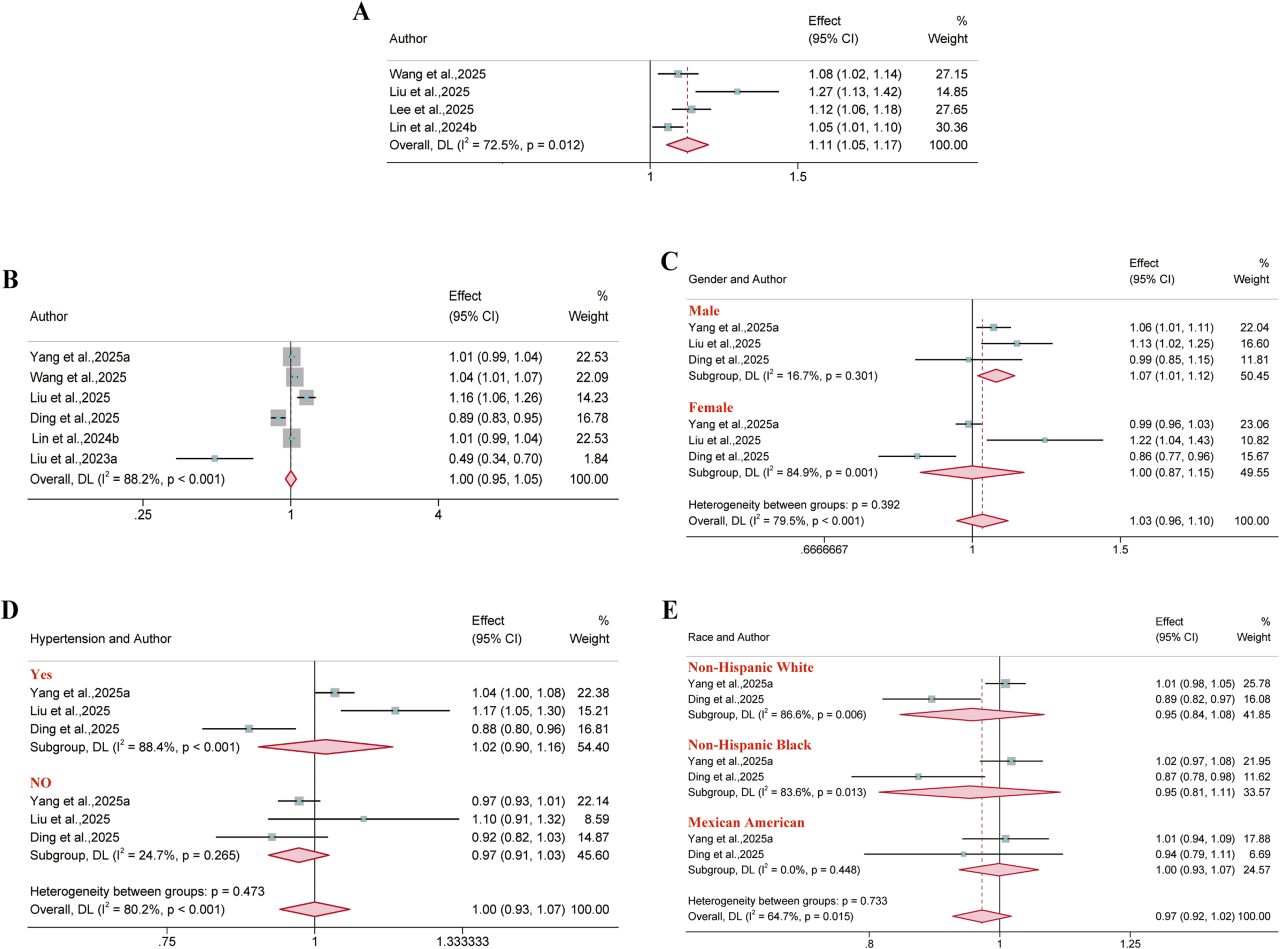
Meta-analysis of the association between BRI and mortality. **(A)** CVD mortality; **(B)** All-cause mortality in the overall population; **(C)** All-cause mortality in gender subgroups; **(D)** All-cause mortality in hypertensive population subgroups; **(E)** All-cause mortality in ethnic subgroups.

Six and four reports included all-cause mortality and CVD mortality outcomes, respectively. Results demonstrated that BRI was significantly associated with all-cause mortality risk, albeit only in the male subgroup (HR = 1.07, 95% CI: 1.01–1.12; I² = 16.7%). Furthermore, a significant positive association was observed between BRI and CVD mortality risk in the overall population. (HR = 1.11, 95% CI: 1.05–1.17; I² = 72.5%) (**Figure 16**).

### 3.4 Diagnostic accuracy meta-analysis

**Figure 17:**
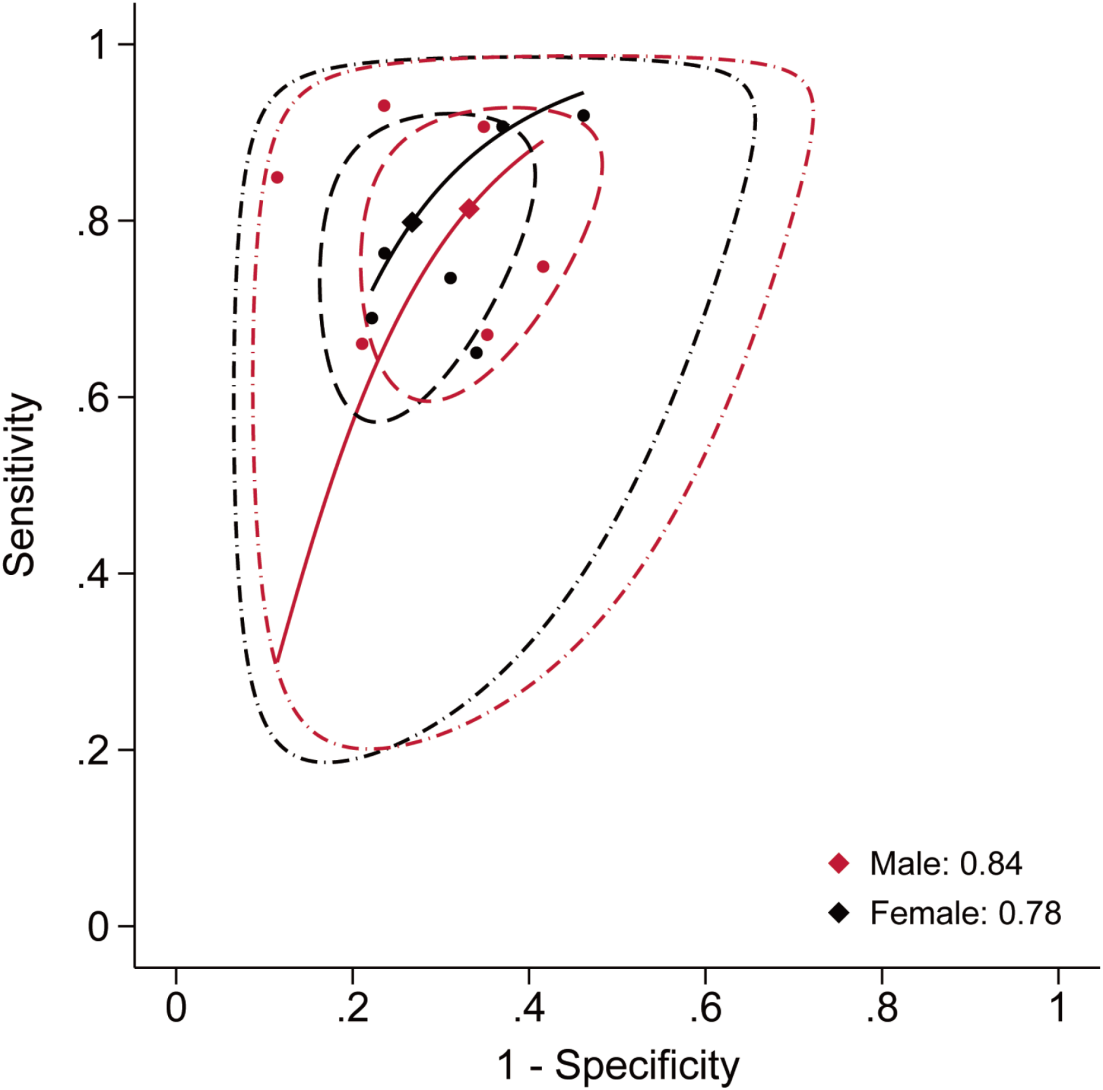
Predictive performance of BRI for metabolic syndrome in males and females.

**Figure 18:**
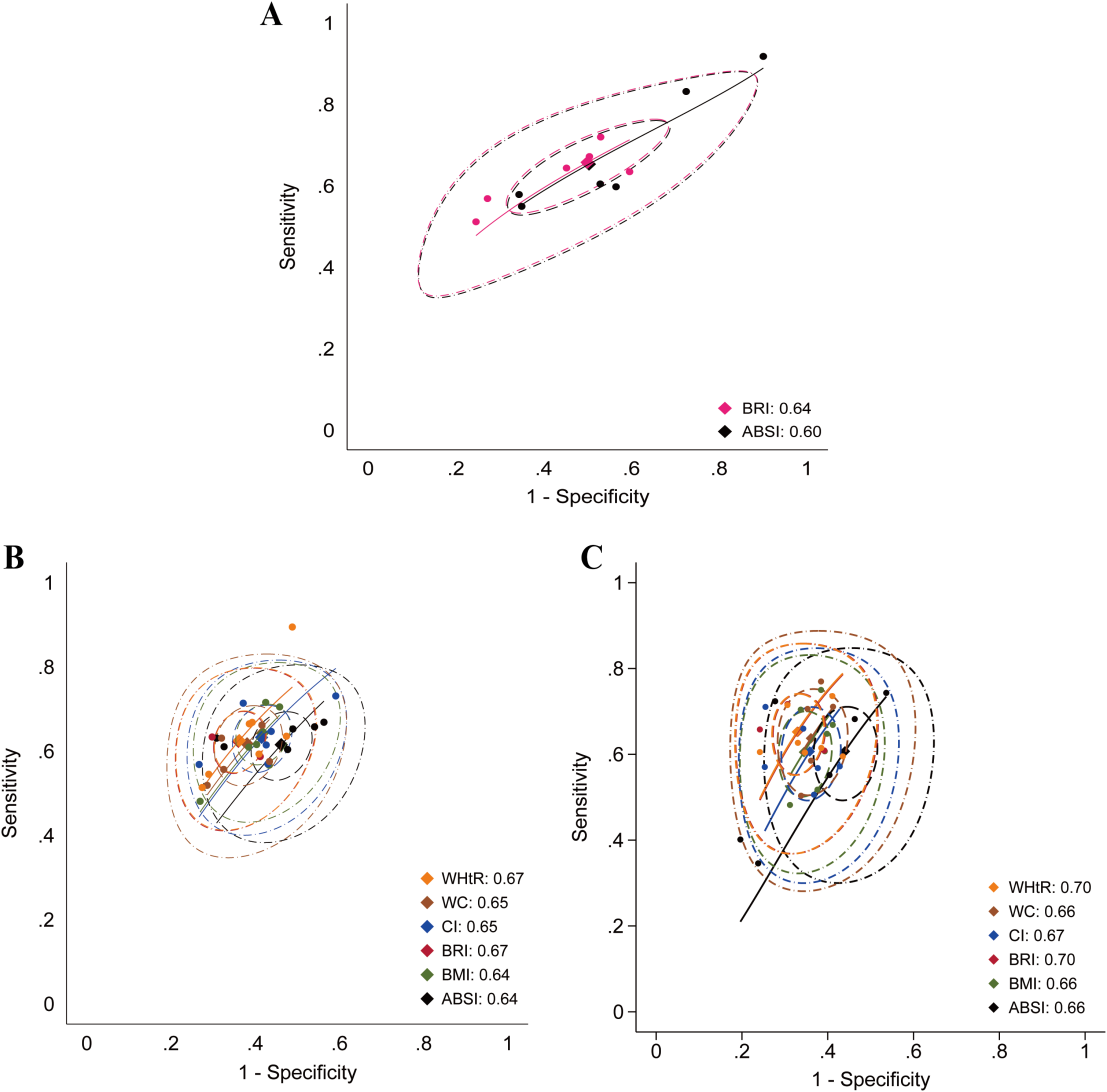
Predictive performance of different anthropometric indices for hypertension. **(A)** Overall population; **(B)** Male population; **(C)** Female population.

**Figure 19:**
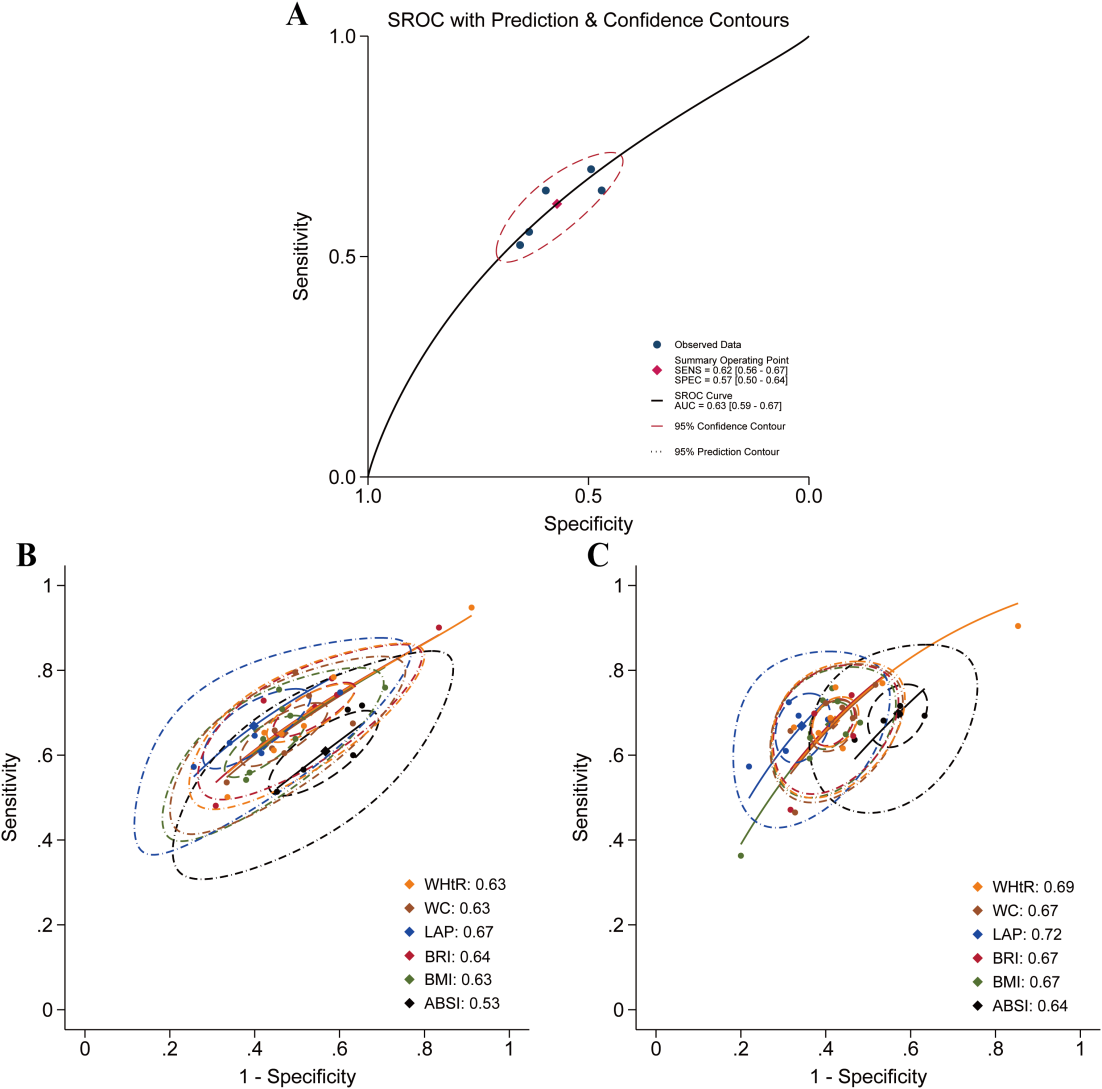
Predictive performance of different anthropometric indices for hyperuricemia. **(A)** Performance of BRI in the overall population; **(B)** Male population; **(C)** Female population.

A further analysis was conducted to assess the diagnostic accuracy of anthropometric indices. Studies focusing on hypertension (n = 13) (44, 54, 58–60, 63, 69, 73, 82, 84, 95, 96, 114), metabolic syndrome (n = 6) (62, 73, 87, 92, 96, 101), and hyperuricemia (n = 11) (31, 32, 60, 64, 73, 90, 93, 96, 97, 102, 113) provided suitable data to calculate diagnostic accuracy metrics. Based on these data, area under the summary receiver operating characteristic (AUC-SROC) curves were plotted (**Figure 17**–**19**). Meta-analysis results demonstrated that BRI exhibited good discriminatory ability in the male subgroup (AUC-SROC = 0.84, 95% CI: 0.80–0.87) and female subgroup (AUC-SROC = 0.78, 95% CI: 0.74–0.81). It also demonstrated certain discriminatory value in the female hypertension subgroup (AUC-SROC = 0.70, 95% CI: 0.66–0.74), but its discriminatory ability was limited in the overall hyperuricemic population (AUC-SROC = 0.63, 95% CI: 0.59–0.67). Furthermore, LAP exhibited good discriminatory ability in the female hyperuricemia subgroup (AUC-SROC = 0.72, 95% CI: 0.68–0.76), and WHtR also demonstrated some discriminatory value in the female hypertension subgroup (AUC-SROC = 0.70, 95% CI: 0.65–0.73). The remaining anthropometric indices exhibited low discriminatory ability in both hypertension and hyperuricemia (AUC-SROC< 0.70).

AUC-SROC results were consistent with pooled AUC values estimated via the inverse variance method. Further pooled diagnostic metrics (sensitivity, specificity, PLR, NLR, and dOR) are presented in **Supplementary Table 3**.

### 3.5 Sensitivity and subgroup analysis

The application of sensitivity analyses and Egger’s tests was restricted to meta - analyses with a minimum of three included studies to ensure the robustness of the conclusions. The results showed that most initial meta-analyses exhibited moderate to high heterogeneity. After excluding studies identified as sources of heterogeneity through sensitivity analysis, the heterogeneity generally decreased, with some dropping to zero. Even when heterogeneity remained high, the pooled results remained largely consistent before and after exclusion. In the meta-analyses examining the associations between different anthropometric indices and the risk of CKM-related outcomes, the pooled estimates for each outcome and the results of sensitivity analyses are comprehensively summarized in **Supplementary Table 4**.

**Figure 20:**
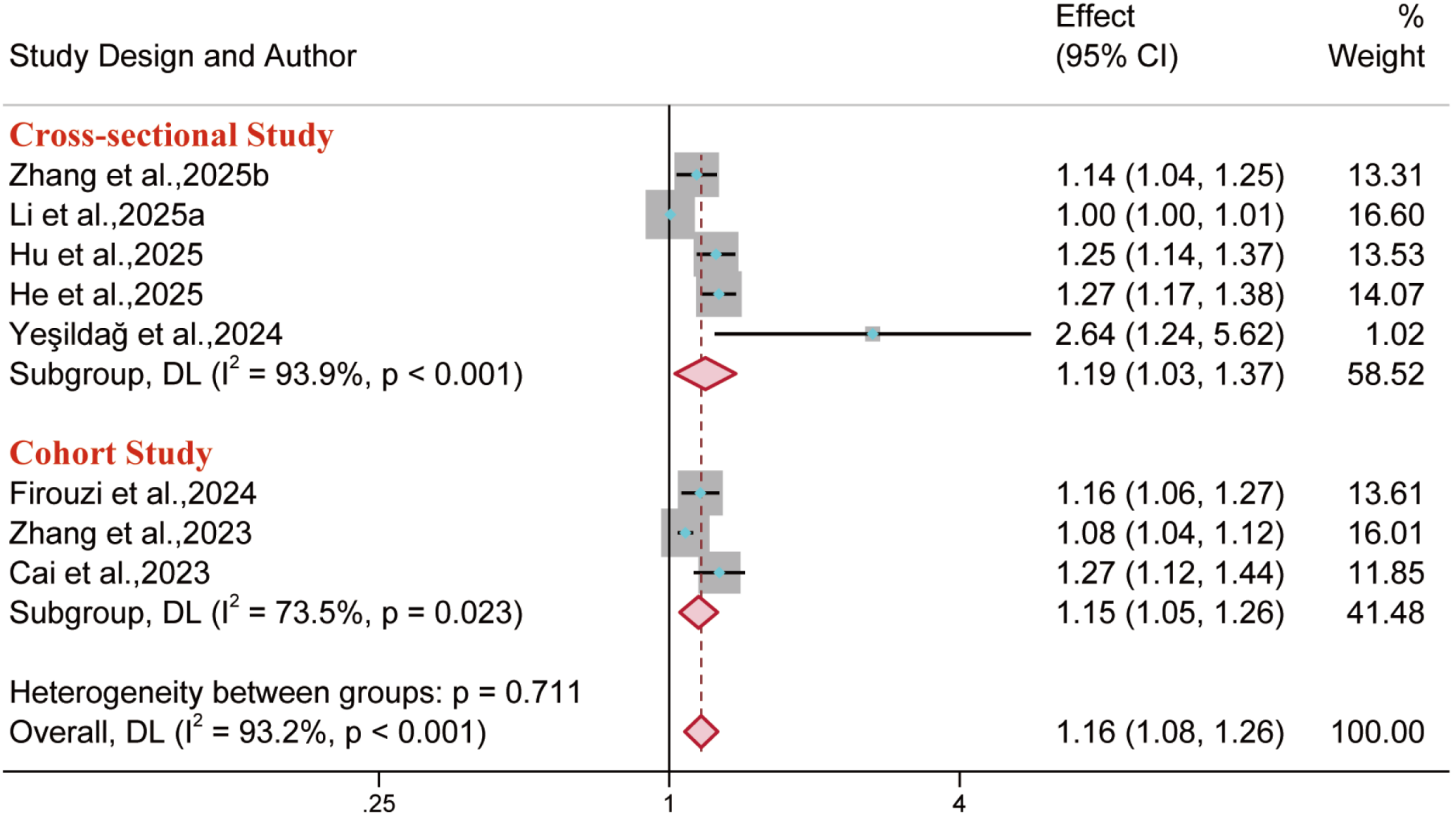
Meta-analysis evaluating the association between BRI and CVD risk according to study design.

Notably, when analyzing the link between BRI and CVD risk in the overall population, extremely high heterogeneity and significant publication bias were observed (I² = 93.2%; Egger’s test *p* = 0.001). Consequently, a subgroup analysis was conducted by study design type (**Figure 20**). The results demonstrated that the cross-sectional subgroup retained significant heterogeneity (I² = 93.9%), while the cohort subgroup exhibited moderate heterogeneity (I² = 73.5%).

Sensitivity analysis of the cross-sectional subgroup identified Li et al., 2025a (71) as the primary source of heterogeneity. Excluding this study yielded a pooled OR of 1.23 (OR = 1.23, 95% CI: 1.13–1.35), with significantly reduced heterogeneity (I² = 57.0%, *p* = 0.073). That study focused on individuals aged 65 years and over, potentially resulting in substantial disparities in population characteristics in comparison to other studies and thus becoming the primary source of heterogeneity. The three studies in the cohort subgroup exhibited heterogeneity due to inconsistencies in country, participant age range, and adjusted covariates. However, the Egger’s test result (*p* = 0.115) showed no evidence of significant publication bias, suggesting reasonable reliability of the findings.

Given that pooling data from studies with different designs may introduce bias, we performed subgroup analyses stratified by study design for the meta-analyses evaluating the associations between BRI and the risk of CVD, diabetes, and type 2 diabetes. The results showed that the positive associations between BRI and these outcomes remained stable, regardless of whether data were pooled with or without study design stratification. Detailed results are presented in **Supplementary Table 5** and **Supplementary Figure 13**. In addition, subgroup analyses stratified by sex were conducted for all eligible CKM-related outcomes. For the all-cause mortality outcome, additional subgroup analyses were performed based on race/ethnicity and hypertension status (**Supplementary Table 4**).

### 3.6 Study quality assessment and publication bias

The quality assessment results for all 93 studies are summarized in **Supplementary Table 6**. Scores ranged from 7 to 13 points, corresponding to a mean score of 9.31 out of 14, with no study attaining the maximum. The main reasons for this outcome are as follows: First, the included studies had a high proportion of cross-sectional designs (59.1%), which inherently have certain methodological limitations. Second, most studies failed to adequately justify their sample sizes. Third, during outcome assessment, a large number of studies did not explicitly report whether outcome assessors were blinded to the exposure status of participants. Another limitation was the lack of repeated outcome assessments during the study period.

**Figure 21:**
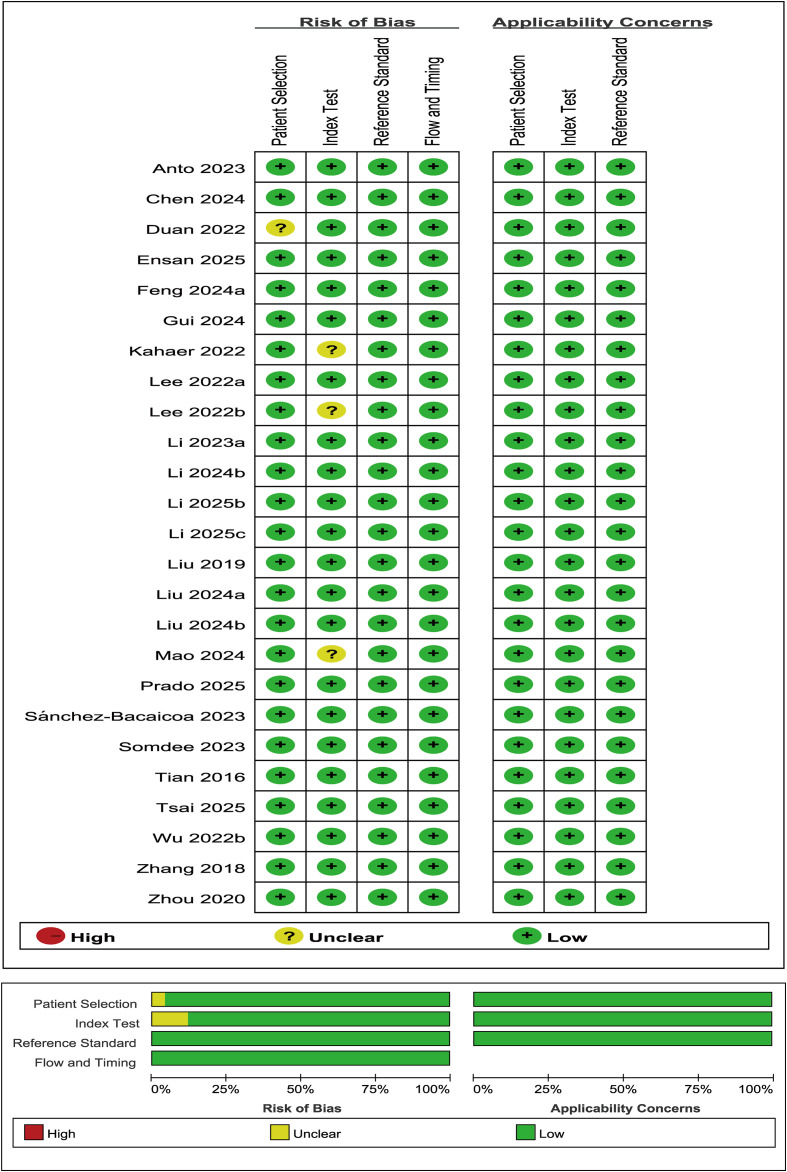
QUADAS-2 tool for quality assessment.

The QUADAS - 2 tool was applied to assess the quality of the 25 studies included within the diagnostic accuracy meta-analysis (**Figure 21**). Assessment results demonstrated that all studies had a low risk of bias in applicability. Regarding risk of bias, most domains had a low risk; however, some limitations were identified. One study (87) had an unclear risk of bias in patient selection, mainly due to unclear random sampling or consecutive enrolment methods. In addition, three studies (90, 95, 113) had an unclear risk of bias in index tests, primarily because they did not report whether test results were interpreted under blinded conditions and lacked pre-specified test thresholds.

Publication bias assessment revealed that, for the risk association meta-analyses, most pooled analyses showed no significant publication bias as determined by Egger’s test (*p* > 0.05). For the 13 analyses with potential publication bias, the results remained robust in sensitivity analyses after excluding studies that contributed to heterogeneity. For the diagnostic meta-analyses, none of the 25 included analyses showed significant publication bias as assessed by Deeks’ funnel plot asymmetry test (*p* > 0.1). Detailed results are presented in **Supplementary Table 3** and **Supplementary Figure 14**-**S16**.

## 4 Discussion

This study systematically synthesized the evidence on the association between BRI and CKM-related outcomes, including metabolic, renal, CVD, and mortality endpoints, and further assessed the diagnostic utility of BRI for specific metabolic risk factors. Our results showed a significant positive association between elevated BRI and the risk of multiple metabolic outcomes. This association was consistent in direction for renal, cardiovascular, and mortality outcomes, although the strength of the association varied across endpoints and by sex. Notably, the association was most pronounced for metabolic syndrome (pooled OR = 2.23 for the overall population), and was markedly weaker for outcomes including CKD (OR = 1.15) and CVD (OR = 1.16). These findings suggest that the body fat distribution pattern captured by BRI is implicated in multiple domains of CKM-related risk. However, its risk prediction value varies by outcome, and its potential clinical utility is most relevant to the early identification of metabolic risk.

In the context of early metabolic risk within the CKM framework, excess adiposity and adipose tissue dysfunction, particularly visceral and ectopic fat deposition, are widely recognized as the core pathophysiological basis (1, 2). For metabolic outcomes, we observed significant positive associations between elevated BRI and the risk of metabolic syndrome, diabetes, and hyperuricemia. Diagnostic accuracy analyses further showed that BRI exhibited favorable discriminatory performance for metabolic syndrome. Although this finding is based on a limited number of studies, it suggests the potential screening utility of BRI for the early detection of metabolic abnormalities. Derived from height and waist circumference, BRI captures trunk fat distribution and may reflect visceral adiposity more accurately than conventional anthropometric indices. This characteristic may explain its association with a wide range of metabolic risks and its relatively robust performance in identifying metabolic syndrome. Previous meta-analyses have also reported the favorable discriminatory performance of BRI for detecting metabolic syndrome, with a slight advantage over conventional anthropometric indices such as BMI (116). Other studies have confirmed its association with the risk of diabetes and CVD, further highlighting its potential utility in CKM risk assessment (78, 117). Taken together, these external findings corroborate our results and highlight the potential utility of BRI for early metabolic risk identification and assessment within the CKM framework.

In the advanced stages of CKM involving the renal and cardiovascular systems, more pronounced heterogeneity was observed in the associations between BRI and corresponding outcomes, with notable sex differences identified in these associations. The primary finding was that a link between BRI and CKD risk was detected in the overall population and in women, but no such association was found in men. This discrepancy may be attributable to gender-specific fat distribution patterns, hormonal environments, and organ susceptibility to metabolic stress (118). A large-scale study of Chinese individuals aged ≥40 years further supports this gender disparity, showing a stronger association between BRI and estimated glomerular filtration rate (eGFR) decline in women (119). Furthermore, women face greater barriers to accessing kidney transplant waiting lists compared with men, a discrepancy that is particularly evident among older and obese populations, thereby exacerbating existing gender disparities (120). Regarding cardiovascular outcomes, the present study demonstrated consistent associations between BRI and CVDs. However, no significant association with heart disease was observed, suggesting heterogeneity in the etiological composition and clinical phenotypes of different cardiovascular endpoints. Several previous prospective cohort studies in Chinese populations with CKM stages 0–3 have also identified BRI as a risk factor for CVD (121). Multicenter cohort studies provide additional evidence linking longitudinal changes in the BRI to cardiovascular risk (122). Recent studies also demonstrate that elevated BRI is associated with advanced CKM syndrome and possesses superior predictive value compared with conventional anthropometric indices (10). Taken together, the potential utility of BRI for multi-perspective renal and cardiovascular risk assessment is supported by this body of evidence. However, given the pronounced heterogeneity identified in several pooled analyses, further investigation is warranted to define its role in the comprehensive risk assessment of CKM-related outcomes.

Regarding mortality outcomes, our findings further highlight their complexity. The present study demonstrated a sex-specific link between BRI and all-cause mortality, which was significant only in men. In the overall population, BRI showed an association with cardiovascular mortality risk. This finding suggests that mortality, as the terminal outcome of CKM progression, is often influenced by both metabolic and non-metabolic factors. Therefore, the explanatory power of a single anthropometric index for mortality risk is inherently constrained (123, 124). Research conducted in Japan, along with several meta-analyses, has indicated a potential U-shaped relationship between BRI and both all-cause and CVD mortality (125). This finding indicates a more complexity in how BRI relate to mortality at the terminal outcome stage. From a broader perspective, a more comprehensive understanding of the interplay between metabolic dysregulation and chronic inflammation, along with the integration of multidimensional pathways spanning biological, social, and environmental factors, is required to modify the trajectory of adverse CKM-related clinical outcomes (126–128). These findings underscore the critical need for stratified risk assessment in the mid-to-late stages of CKM progression, taking into account gender differences and specific outcome types. However, given the multifactorial nature of mortality endpoints, caution should be exercised when interpreting the strength of the observed association and its corresponding clinical implications.

From a mechanistic perspective, the aberrant body fat distribution pattern captured by BRI may be implicated in the development of CKM-related outcomes via multiple pathophysiological pathways. Strong correlation between BRI and radiologically measured visceral adipose tissue has been confirmed in previous studies (9, 129). Unlike subcutaneous fat, higher metabolic activity is exhibited by visceral fat, by which CKM health can be affected through multiple pathophysiological mechanisms. The secretion of pro-inflammatory adipokines (e.g., tumor necrosis factor-α [TNF-α], interleukin-6 [IL-6]) is upregulated by visceral fat accumulation, with a chronic low-grade systemic inflammatory state induced (130). Meanwhile, insulin resistance is promoted by excessive free fatty acid release through lipotoxicity, and glucose and lipid homeostasis is subsequently disrupted (131). Furthermore, the renin-angiotensin-aldosterone system can be activated by visceral fat, which drives sodium and water retention and blood pressure elevation, contributing to the pathogenesis of hypertension and renal injury (132). Ectopic fat deposition in key organs including the liver, myocardium, and pancreas can also be induced by excess visceral fat, which is respectively associated with fatty liver disease, myocardial dysfunction, and impaired pancreatic β-cell function (6). Taken together, the core pathophysiological basis linking visceral fat-mediated metabolic dysregulation to renal damage and CVD is likely constituted by these mechanisms. This integrative framework explains the associations between BRI and the full spectrum of CKM-related outcomes observed in our study.

## 5 Limitations

By systematically synthesizing available evidence on BRI across the full spectrum of CKM-related outcomes, this study further highlights the central role of visceral adiposity and aberrant body fat distribution in CKM-related dysfunction and adverse clinical prognosis. Key strengths of this study include the inclusion of a large number of eligible studies with substantial sample sizes covering diverse multinational populations, as well as the systematic evaluation of BRI across multiple CKM-related outcomes from both risk association and diagnostic performance perspectives.

However, several limitations should be acknowledged. First, most of the included studies were cross-sectional in design, which precludes definitive causal inferences, as residual confounding and reverse causality are inherent to this study type. Although most included primary studies conducted multivariable adjustment, residual confounding inherent to observational study designs may still affect the strength of the observed associations and the clinical interpretation of our findings.

Second, substantial heterogeneity was observed in several meta-analyses, which likely arose from between-study variations in outcome definitions, covariate adjustment strategies, and baseline population characteristics. This finding indicates significant clinical and methodological diversity across studies, and thus the corresponding pooled effect estimates should be interpreted with caution. We performed sensitivity and subgroup analyses to explore sources of heterogeneity, and heterogeneity was attenuated in several analyses. However, substantial heterogeneity persisted in a subset of results, indicating that the stability and precision of the corresponding pooled estimates may be limited. For these results, emphasis should be placed on the direction of the association and overall trend. They should be interpreted as exploratory syntheses of available evidence, rather than precise quantifications of effect size.

Furthermore, the relatively modest pooled effect sizes for certain outcomes (e.g., CKD, CVD), combined with the inability to fully eliminate residual confounding, suggest that BRI may represent an epidemiological association rather than acting as a robust, independent predictor with immediate clinical utility. The robustness of pooled estimates for specific outcomes was further limited by the small number of eligible studies included in the corresponding analyses.

Importantly, most included primary studies evaluated individual CKM-related outcomes, rather than transitions between CKM syndrome stages. Therefore, our findings primarily provide evidence for the associations between BRI and multiple CKM-related outcomes, rather than direct validation of the association between BRI and CKM stage progression. High-quality prospective cohort studies are warranted to further define the long-term predictive value and clinical utility of BRI for CKM-related risk assessment.

## 6 Conclusions

Within the CKM framework, the body fat distribution pattern captured by BRI has been found to be associated with the risk of metabolic, renal, CVD, and mortality endpoints, with substantial heterogeneity observed across outcome categories and study populations. The central role of visceral adiposity in CKM-related outcomes is highlighted by these findings, which confirm that BRI, as a validated measure of aberrant body fat distribution, can provide incremental information for the identification and risk stratification of CKM-related risks. Further definition of the long-term predictive value and clinical utility of BRI across diverse populations and all stages of CKM is warranted through high-quality prospective cohort studies incorporating multidimensional risk integration models.

## Data Availability

The original contributions presented in the study are included in the article/**Supplementary Material**. Further inquiries can be directed to the corresponding author.
