# Supplementary material for: Risk association and diagnostic value of body roundness index for cardiovascular-kidney-metabolic-related outcomes: a systematic review and meta-analysis
Source: Front Endocrinol (Lausanne). 2026 Apr 14;17:1814762. doi: 10.3389/fendo.2026.1814762 (PMC13121168; doi:10.3389/fendo.2026.1814762)
Supplement: Supplementary file 1 [file DataSheet1.docx]

| [Search Strategy in Databases 2](#_Toc24465)  [Suppl. Table 1 Basic characteristics of the studies included in the review 3](#_Toc32015)  [Suppl. Table 2 Measures of the studies included in the meta-analysis. 16](#_Toc13599)  [Suppl. Table 3 Pooled accuracy parameters in the prediction of CKM Syndrome-related diseases. 48](#_Toc835)  [Suppl. Table 4 The association between different anthropometric indices and the risk of CKM Syndrome-related diseases. 49](#_Toc32266)  [Suppl. Table 5 The subgroup analysis by study design. 57](#_Toc17804)  [Suppl. Table 6 Quality assessment of studies included in the review 58](#_Toc20786)  [Suppl. Fig. S1. Pooled AUC values of different anthropometric indices for predicting diabetes risk. (A) Overall population; (B) Male population; (C) Female population. 63](#_Toc10926)  [Suppl. Fig. S2. Pooled AUC values of different anthropometric indices for predicting type 2 diabetes risk in the overall population. 63](#_Toc37)  [Suppl. Fig. S3. Pooled AUC values of different anthropometric indices for predicting type 2 diabetes risk. (A1-A2) Male population; (B1-B2) Female population. 64](#_Toc5229)  [Suppl. Fig. S4. Pooled AUC values of different anthropometric indices for predicting dyslipidemia risk in the overall population. 64](#_Toc10814)  [Suppl. Fig. S5. Pooled AUC values of different anthropometric indices for predicting dyslipidemia risk. (A) Male population; (B) Female population. 65](#_Toc32445)  [Suppl. Fig. S6. Pooled AUC values of different anthropometric indices for predicting hyperuricemia risk in the overall population. 65](#_Toc21920)  [Suppl. Fig. S7. Pooled AUC values of different anthropometric indices for predicting hyperuricemia risk in the male population. 66](#_Toc20804)  [Suppl. Fig. S8. Pooled AUC values of different anthropometric indices for predicting MAFLD risk. (A) Overall population; (B) Male population; (C) Female population. 66](#_Toc9502)  [Suppl. Fig. S9. Pooled AUC values of different anthropometric indices for predicting NAFLD risk. (A) Male population; (B) Female population. 67](#_Toc15918)  [Suppl. Fig. S10. Pooled AUC values of different anthropometric indices for predicting hypertension risk in the overall population. 67](#_Toc14929)  [Suppl. Fig. S11. Pooled AUC values of different anthropometric indices for predicting hypertension risk. (A) Male population; (B) Female population. 68](#_Toc8293)  [Suppl. Fig. S12. Pooled AUC values of different anthropometric indices for predicting chronic kidney disease risk in the overall population. 68](#_Toc3429)  [Suppl. Fig. S13. The subgroup analysis by study design. (A) Diabetes Mellitus; (B) Type 2 diabetes. 69](#_Toc7326)  [Suppl. Fig. S14. Publication bias assessment for included hypertension studies via Deek’ s Funnel Plot Asymmetry Test. 70](#_Toc4210)  [Suppl. Fig. S15. Publication bias assessment for included metabolic syndrome studies via Deek’s Funnel Plot Asymmetry Test. 70](#_Toc9196)  [Suppl. Fig. S16. Publication bias assessment for included hyperuricemia studies via Deek’s Funnel Plot Asymmetry Test. 71](#_Toc25166) Search Strategy in Databases | |
| --- | --- |
| databases | search strategy |
| Pubmed | (body roundness index[Title/Abstract]) OR (BRI[Title/Abstract]) |
| Web of Science | TS=(body roundness index OR BRI) |
| Embase | body roundness index':ti,ab,kw OR ‘BRI’:ti,ab,kw |

| Suppl. Table 1 Basic characteristics of the studies included in the review | | | | | | | | | | | |  |
| --- | --- | --- | --- | --- | --- | --- | --- | --- | --- | --- | --- | --- |
| Study  ID | Author (year) | Country | Study design | Data source | Sample size(% WoMale) | Disease status | Age range | Follow-up years | BRI determination | Statistical method | AdjustMalet  for  confounders | |
|  |  |  |  |  |  |  |  |  |  |  |  |  |
| 1 | Zhou et al.,2025 | China | Prospective Cohort Study | China Health and RetireMalet  Longitudinal Study | 10,785 (52.22%) | NA | ≥45 | 7.72 | Objectively | χ2 test;ANOVA; Kruskal–Wallis H test; univariable and multivariable Cox regression;likelihood ratio tests | age, gender, education,  smoking and drinking status sleep duration, physical activity, Dyslipidemia, FPG), TC, triglycerides (TG), HDL, LDL, HbA1c | |
| 2 | Zhang et al.,2025a | China | Prospective Cohort Study | Risk Evaluation of  cAncers in Chinese diabeTic Individuals: a lONgitudinal study | 6,575 (66.30%) | NA | ≥40 | 3.19 | Objectively | Mann–Whitney U  test;chi-square test;Multivariate Cox proportional hazards regression;Restricted cubic spline;Stratified analyses;Sensitivity analyses | sex, smoking habits, drinking habits, exercise, family Diabetes history and age,triglyceride,total cholesterol,high-density lipoprotein cholesterol,low-density lipoprotein cholesterol,systolic blood pressure,diastolic blood pressure,fasting blood glucose,2-h postprandial blood glucose,glycated haemoglobin | |
| 3 | Zhang et al.,2025b | China | Retrospective Cohort Study | China Health and  RetireMalet Longitudinal Study | 2,322 (65.70%) | Circadian  Syndrome | ≥45 | 9 | Objectively | multivariate logistic  regression models;smoothing function analysis | age, sex, education level, residence, marital status, smoking status, alcohol consumption, physical activity | |
| 4 | Yin et al.,2025 | China | Cross-sectional Study | a population-based cohort study conducted at Ningxia Medical University | 14,926 (60.20%) | NA | 35-74 | NA | Objectively | Chi-Square test; Pearson correlation; Receiver operating characteristic | age and sex | |
| 5 | Tsai et al.,2025 | China | Prospective Cohort Study | Cardiorespiratory Fitness and Health in Eastern  Armed Forces study | 2,448 (12%) | Physically Fit  Participants | 18-39 | 6 | Objectively | Multivariable Cox hazards  regression analysis; Area Under the Curve | age, sex, substance use,  physical activity levels, systolic blood pressure, diastolic blood pressure, total cholesterol, serum uric acid, blood urea nitrogen and serum creatinine; | |
| 6 | Radmehr et al.,2025 | Iran | Cross-sectional Study | Fasa Cohort  Study | 10,081 (54.97%) | NA | 35-70 | NA | Objectively | t-tests ; chi-square tests;binary logistic regression;ROC curve analysis | age, gender, physical activity, Hypertension,Diabetes,hyperlipidemia, energy intake, fat intake, vegetables and fruit intake | |
| 7 | Peng et al.,2025 | China | Multicenter Retrospective  Cohort Study | China Health and  RetireMalet Longitudinal Study | 7,651 (36.1%) | NA | ≥45 | 9 | Objectively | weighted Cox regression  model;one-way ANOVA;chi-square tests;Cox regression models;trend tests;linear regression model | age, level of education,  place of residence , marital status , gender, insurance，status, hypertension , hyperglycaemia, dyslipidaemia, Maletal illness, information on the types of therapy | |
| 8 | Mungvongsa et al.,2025 | Thailand | Cross-sectional Study | Originally designed | 505 (71.9%) | NA | 28-59 | NA | Objectively | chi-square test; Mann-Whitney U test; multivariate  logistic regression | age, comorbidities, smoking, drinking, and physical  activity | |
| 9 | Saadati et al.,2025 | the United States | Prospective Cohort Study | Atherosclerosis Risk  in Communities (ARIC) cohort study | 12,085 (55.04%) | NA | 45-64 | 27 | Objectively | Longitudinal targeted  maximum likelihood estimation model;super learner algorithms (Bayesian GLM, SL.xgboost, Generalized linear model) | age , gender, race, education level, occupation, marital status, family history of Cardiovascular Diseasess, and total calorie intake | |
| 10 | Li et al.,2025a | the United States | Cross-sectional Study | National Health and Nutrition Examination Survey | 3,584 (50.06%) | NA | ≥65 | NA | Objectively | Univariate and multivariate  regression analyses;mediation model | sex, race, education level,  ratio of family income to poverty, smoking history, alcohol consumption history | |
| 11 | Li et al.,2025b | China | Cross-sectional Study | the Hospital of Nanhai  Economic DevelopMalet Zone,Guangdong Province, China | 8,362 (54.99%) | NA | ≥65 | NA | Objectively | chi-squared test;t tests;Mann–Whitney U tests;logistic regression analysis;ROC curves | obesity-related index, age,  sex,Hypertension, Diabetes Mellitus, smoking status, drinking status,systolic blood pressure, diastolic blood pressure, fasting plasma glucose, white blood cell count, red blood cell count, hemoglobin, platelet count, alanine aminotransferase, aspartate aminotransferase, serum creatinine, low-density lipoprotein cholesterol, high-density lipoprotein cholesterol, triglyceride, total cholesterol | |
| 12 | Hu et al.,2025 | China | Prospective Cohort Study | China Health and RetireMalet Longitudinal Study | 4,458 (52.76%) | NA | ≥45 | 9 | Objectively | Wilcoxon rank test;Chi-square test;logistic regression; | age, sex, education level, marital status, residential area, smoking pack years, alcohol use, hypertension, diabetes, and hyperlipidemia | |
| 13 | Li et al.,2025c | China | Cross-sectional Study | Taihu Sanatorium  of Jiangsu Province | 14,834 (34.02%) | NA | ≥18 | NA | Objectively | chi-square test;  one-way analysis of variance;independent samples t-test;Mann-Whitney test;logistic regression analysis;ROC curve; | age, systolic blood pressure,   diastolic blood pressure, fasting plasma glucose smoking, alcohol consumption | |
| 14 | He et al.,2025 | the United States | Cross-sectional Study | National Health and Nutrition Examination Survey | 6,640 (53.57%) | Cardiometabolic  Syndrome | ≥20 | NA | Objectively | chi-square test; t-test;Multivariate logistic regression; | age, gender, and race,  education level, smoking status, diabetes, hypertension, Body mass index, Systolic blood pressure, diastolic blood pressure | |
| 15 | Gan et al.,2025 | the United States | Cross-sectional Study | National Health and Nutrition Examination Survey | 39,454 (50.60%) | NA | ≥20 | NA | Objectively | chi-square test;t-test; multivariate logistic regression;restricted cubic splines | Age, Sex, race,Marital  status, poverty income ratio, Education level, Body mass index, Alcohol, Smoking,Hypertension, Diabetes Mellitus, Hyperlipidemia | |
| 16 | Ensan et al.,2025 | Iran | Prospective Cohort Study | Mashhad Stroke and Heart  Atherosclerotic Disorder study | 7,561 (60.02%) | NA | 35 - 65 | 10 | Objectively | Kolmogorov–Smirnov test;t-test;Logistic regression models; ROC curve analysis;the Youden index | age, sex, job status, education,  marital status, physical activity levels (PAL), energy intake, depression, anxiety | |
| 17 | Prado et al.,2025 | Brazil | Cross-sectional Study | The 2013 National  Health Survey (PNS) is a national household-based survey carried out by the Oswaldo Cruz Foundation and the Ministry of Health in partnership with the Brazilian Institute of Geography and Statistics. | 6,221 (57.74%) | NA | 18-59 | NA | Objectively | Pearson’s chi-square  test;t-test;receiver operating characteristic (ROC) curve;area under the curve; | NA | |
| 18 | Yang et al.,2025a | the United States | Prospective Cohort Study | National Health and  Nutrition Examination Survey | 5,371 (60%) | Excluded individuals with  a prior diagnosis of myocardial infarction , stroke , congestive heart failure and cancer | ≥65 | NA | Objectively | multivariate Cox  regression models, restricted cubic spline, likelihood ratio test, Kaplan–Meier method | age, gender, race,  education level, household income and poverty ratio, alcohol drinking, Hypertension and Diabetes | |
| 19 | Wang et al.,2025 | the United States | Prospective Cohort Study | National Health and  Nutrition Examination Survey | 15,848 (47.78%) | Diabetes and  PreDiabetes | ≥20 | 7.74 | Objectively | Chi-square test ,  Kruskal-Wallis test,Multivariate Cox regression models,Restricted cubic splines | age, gender, race,education,  household income and poverty ratio, access to healthcare, health insurance, Healthy Eating Index, smoking, drinking, Hypertension, and Cardiovascular Diseases | |
| 20 | Liu et al.,2025 | the United States | Prospective Cohort Study | National Health and  Nutrition Examination Survey | 8,227 (48.51%) | Diabetes | ≥21 | 7.25 | Objectively | Multivariate Cox regression models,Restricted cubic splines | age, gender, race, education,  BMI, smoke, drinking, duration, medication, physical activity, duration, medication, Hypertension, hypercholesteremia and cancer | |
| 21 | Lee et al.,2025 | the United States | Prospective Cohort Study | Third National  Health and Nutrition Examination Survey | 6,746 ( 51.4%) | NA | 40–79 | 20.3 | Objectively | Student’s t-test,  Pearson correlation analysis, Univariate and multivariable Cox proportional hazard regressions | age | |
| 22 | Ding et al.,2025 | the United States | Prospective Cohort Study | National  Health and Nutrition Examination Survey | 1,596 (72.59%) | Osteoporotic | ≥50 | 6.25 | Objectively | survey-weighted  chi-squared tests,survey-weighted linear regression models, Kaplan–Meier analysis, survey-weighted Cox proportional hazards regression models,log-likelihood ratio test,Sensitivity analyses | age group, gender, race,  education level, family PIR, smoker, heavy drinker, calcium, 25-OHD, hypertension, dyslipidemia,Cardiovascular Diseases,Diabetes, liver disease, cancer, and kidney failure | |
| 23 | Yang et al.,2025b | China | Retrospective Cohort Study | China Health and  RetireMalet Longitudinal Study | 1620 (55.2%) | NA | ≥45 | 4 | Objectively | Multiple logistic  regression;Receiver-operating characteristic (ROC) | age, eGFR base value,  Hypertension, arthritis or rheumatism, education, and CRP | |
| 24 | Rezaee et al.,2025 | Iran | Prospective Cohort Study | Originally designed | 6935 (58.9%) | NA | 35-65 | 10 | Objectively | chi-square test;  t-test;logistic regression | age, sex, marriage, job,  education, and smoking | |
| 25 | Fei et al.,2025 | the United States | Cross-sectional Study | National  Health and Nutrition Examination Survey | 6,971 (48.49%) | Diabetes | ≥20 | NA | Objectively | weighted t-tests ,  weighted Wilcoxon rank-sum tests, weighted multivariable logistic regression | age, sex, and race/ethnicity, education level, poverty-to-income ratio (PIR), systolic blood pressure (SBP), diastolic blood pressure (DBP), glycated hemoglobin A1c (HbA1c), fasting plasma glucose (FPG), triglycerides (TG), total cholesterol (TC), albumin (ALB), alanine aminotransferase (ALT), alkaline phosphatase (ALP), total bilirubin (TBIL), hemoglobin (Hb), red blood cell count (RBC), white blood cell count (WBC), platelet count (PLT), triglyceride-glucose index (TyG), uric acid (UA), blood urea nitrogen (BUN), serum creatinine (SCr), albumin-to-creatinine ratio (ACR), estimated glomerular filtration rate (eGFR), and cancer history, medication use | |
| 26 | Chen et al.,2025 | the United States | Cross-sectional Study | National  Health and Nutrition Examination Survey | 24,162 (50.9%) | NA | ≥20 | NA | Objectively | multivariate linear  regression model, Restricted cubic spline (RCS) regression, Receiver-operating characteristic | age, sex, race, smoking status,  drinking status , Physical activity, Calorie intake | |
| 27 | Cao et al.,2025 | the United States | Cross-sectional Study | National  Health and Nutrition Examination Survey | 41,953 (51.59%) | NA | ≥20 | NA | Objectively | Chi-square tests,  linear regression models, weighted multivariate regression | age, gender, and race,education attainMalet, marital status, family PIR, smoking status, Hypertension, BG, TP, TC, TG, ALT, and AST | |
| 28 | Demirci et al.,2024 | Turkey | Cross-sectional Study | Originally designed | 578 ( 68.7%) | NA | ≥18 | NA | Objectively | chi-squared test; ROC; Mann–Whitney U test ; Student’s t-test;area under the curve;logistic regression analysis | age, education, and physical activity | |
| 29 | Gui et al.,2024 | China | Cross-sectional Study | China Health and  RetireMalet Longitudinal Study | 9,488 (54.11%) | NA | ≥45 | NA | Objectively | chi-square test; t-test;ROC analysis;logistic regression | age, educational levels,  marital status, current residence, smoking,alcohol drinking,taking activities, having regular exercises, chronic diseases, serum uric acid | |
| 30 | Zhao et al.,2024 | the United States | Cross-sectional Study | National Health and Nutrition Examination Survey | 4,210 （Raw data  on the  Male-to-Female ratio were not provided.） | NA | ≥20 | NA | Objectively | variance inflation factor； multivariate logistic regression models；smooth curve fittings； threshold effect analysis；smoothing function；sensitivity analyses | sex, age, family poverty income ratio, Diabetes, Hypertension, hyperlipidemia, estimated Glomerular Filtration Rate, alanine transaminase, aspartate aminotransferase, High-Density Lipoprotein cholesterol, MetS | |
| 31 | Zhang et al.,2024a | China | Cross-sectional Study | China Health and  RetireMalet Longitudinal Study | 9,488 (54.11%) | NA | ≥45 | NA | Objectively | Chi-square test; one-way ANOVA ;independent sample t-test;binary logistic regression;receiver operating characteristic curve (ROC);area under the curve (AUC) | age, educational levels,  marital status, current residence, current smoking, alcohol drinking, taking activities, having regular exercises, chronic diseases | |
| 32 | Zhang et al.,2024b | the United States | Cross-sectional Study | National Health and  Nutrition Examination Survey | 20,564 (49.06%) | NA | 18-80 | NA | Objectively | Kruskal–Wallis rank  sum test;Multiple logistic regression analysis;Fisher’s exact test ;Receiver operating characteristic (ROC) curves | Gender; Age; Race;  Education level; Monthly family income; Avg alcohol drinks/day—past 12 months; Marital status; Activities time (Minutes)/day; Cigarettes/day during past 30 days | |
| 33 | Zhan et al.,2024 | China | Prospective Cohort Study | Guizhou Population Health  Cohort Study | 5,230 (54.2%) | NA | 18-95 | 6.64 | Objectively | Wilcoxon rank-sum; Chi-square test;Cox proportional hazards model; accelerated failure time model | age, gender,ethnicity, residence, sleep duration, oil intake, salt intake, vegetable intake, fruit intake, smoking, harmful drinking, sedentary time and educational level;amily history of Hypertension, Diabetes, TC, TG, HDL-C, LDL-C | |
| 34 | Yeşildağ et al.,2024 | Turkey | Cross-sectional Study | Originally designed | 281 (46.6%) | Obstructive Sleep  Apnea Syndrome | ≥18 | NA | Objectively | Kolmogorov–Smirnov test;  Q-Q plot; box plot; skewness; kurtosischi-square test;independent t-test;One-Way Analysis of Variance (ANOVA);Pearson correlation test; | NA | |
| 35 | Wang et al.,2024 | China | Retrospective Cohort Study | China Health and  RetireMalet Longitudinal Study | 7,902 (53.96%) | NA | 45-64 | 4 | Objectively | t-test; chi-squared  test;binary logistic regression; receiver operating curve; | age, education, marital status,  current residence, current smoking, alcohol consumption, activity participation, regular exercise, and chronic disease | |
| 36 | Sekgala et al.,2024 | South Africa | Cross-sectional Study | first South African National  Health and Nutrition Examination Survey (SANHANES-1) | 2,623 (100%) | NA | ≥20 | NA | Objectively | Kolmogorov–Smirnov test; t test and Mann–Whitney U test;chi-square test;Logistic regression analysis; likelihood ratio test;receiver operating characteristics (ROC) analysis;Youden index | age, population group, residence and employMalets | |
| 37 | Sadeghi et al.,2024 | Iran | Cross-sectional Study | Isfahan Diabetes Prevention  Study | 2,082  (NA) | First-degree relatives  of diabetic patients | 30-70 | NA | Objectively | Student’s t test;;receiver operating characteristic (ROC) curve;logistic regression;Youden index | NA | |
| 38 | Mao et al.,2024 | the United States | Cross-sectional Study | National Health and  Nutrition Examination Survey | 29,310 (51.3%) | NA | ≥20 | NA | Objectively | multivariate logistic  regression;weighted smoothed curve;og-likelihood ratio | age,gender,race,marital  status , education,Diabetes,blood pressure,PIR,total water,total kcal,total sugar,total fat,smoked,physical activity,alcohol use,serum cholesterol,kidney stone,coronary artery disease,serum glucose,asthma,serum triglycerides, cancers | |
| 39 | Mansoori et al.,2024 | Iran | Cross-sectional Study | Ashhad Stroke and Heart  Atherosclerotic Disorder | 9,704 (60.04%) | NA | 35-65 | NA | Objectively | Chi-square and  Fisher’s exact tests;independent T test;Logistic regression; | age and gender | |
| 40 | Liu et al.,2024a | China | Prospective Cohort Study | China Health and  RetireMalet Longitudinal Study | 5,873 (54.55%) | NA | ≥45 | 4 | Objectively | t-test; chi-squared test; Logistic regression analyses;area under the curve;receiver operating characteristic (ROC) curve;Youden index | age, educational levels,  places of residence, drink history, smoke history, marital status, SBP,history of Hypertension, LDL, CRP, creatinine, BUN, FPG, TG, and SUA for BMI, WC, BRI, WHtR, and CI and for LAP, VAI, and CVAI, without TG | |
| 41 | Liu et al.,2024b | China | Prospective Cohort Study | Risk Evaluation of Cancers  in Chinese Diabetic Individuals: A Longitudinal Study | 4,112 (75.44%) | NA | ≥40 | NA | Objectively | Kolmogorov-Smirnov test;  U-tests;Binary logistic regression;multivariate logistic regression; 3-knots restricted cubic spline;receiver operating characteristic (ROC) curves;area under the curve | age, marital status, educational  level, a history of Hypertension, Diabetes, Coronary Heart Disease, smoking status, drinking status, creatinine, LDL, ALT, AST, and HOMA-IR | |
| 42 | Lin et al.,2024a | Japan | Prospective Cohort Study | NAfld in the Gifu Area, Longitudinal Analysis)study | 12,940 (49.5%) | NA | 18-79 | 6 | Objectively | Student's t, Mann–Whitney U and chi-squared tests;log-rank test;Cox proportional hazards models;Restricted cubic spline analysis;area under the receiver operating characteristic curve | age, sex, smoking status, alcohol consumption and exercise habits,AST, ALT, GGT and SBP | |
| 43 | Li et al.,2024b | the United States | Cross-sectional Study | National Health and  Nutrition Examination Survey | 23,478 (49.87%) | NA | ≥18 | NA | Objectively | Student’s t-test;  chi-square test ;non-parametric tests ; Fisher’s exact;multivariate logistic regression;area under the curve;Receiver operating characteristic (ROC) curve;decision curve analysis;DeLong’s test;sensitivity analysis | age, sex, race/ethnicity, education  level, PIR, smoking, drinking, MET, eGFR, antidiabetic medication, lipid-lowering medication | |
| 44 | Konstantinova et al.,2024 | Russia | Cross-sectional Study | Originally designed | 347 (0%) | NA | 27-63 | NA | Objectively | Student's tests ;  Mann‒Whitney tests; Kruskal‒Wallis tests ;Logistic regression | under harmful working  conditions | |
| 45 | Firouzi et al.,2024 | Iran | Prospective Cohort Study | Tehran Lipid and Glucose Study | 1,681 (100%) | NA | >20 | 16 | Objectively | Kolmogorov– Smirnov  test; ANOVA tests;Kruskal-Wallis tests;Chi-squared test ;Fisher exact test;Trajectory analysis;Cox proportional hazard models | age, Physical activity,  Smoking history, Maleopausal status, parity ,Family history of Cardiovascular Diseases, | |
| 46 | Feng et al.,2024a | China | Cross-sectional Study | The National Cohort  of Esophageal Cancer-Prospective Cohort Study of Esophageal Cancer and Precancerous Lesions based on High-Risk Population | 10,432 (56.36%) | NA | 40-69 | NA | Objectively | t-test ; χ^2^ test;area  under the curves;receiver operating characteristic ;DeLong test;Multivariable logistic regression | sex, age, marital status,  education, household annual income, smoking, alcohol consumption, vegetable and fruit intake, physical activity, family history of common chronic disease, history of medication for common chronic disease | |
| 47 | Feng et al.,2024b | China | Cross-sectional Study | Thyroid Disorders, Iodine Status and Diabetes Epidemiology (TIDE) study | 65,231 (52.18%) | NA | 18-80 | NA | Objectively | χ^2^ tests; Fisher's exact  tests;area under the curve (AUC);multivariate logistic regression analysis;restricted cubic splines | sex, age, education, income,  smoking status, urban or rural residence, ethnicity | |
| 48 | Hosseini et al.,2024 | Iran | Cross-sectional Study | Hoveyzeh Cohort  Study | 7,836  (59.2%) | at least one of the following three conditions: obesity, Type 2 Diabetes, and metabolic dysregulation | 35-70 | NA | Objectively | Kolmogorov-Smirnov test; t-test ; Mann-Whitney U test; Chi-squared test ;Linear regression analysis; | age, dietary intake (energy, fat, protein, and carbohydrate), and wrist circumference | |
| 49 | Fakhrolmobasheri et al., 2024 | Iran | Prospective Cohort Study | Isfahan Cohort Study | 128 (59.5%) | Prediabetic | >35 | 13 | Objectively | Pearson’s chi-square  test;independent sample T-test; Cox regression;receiver operating characteristics (ROC) curve | physical activity,  low HDL-c, FBG,age,weight, waist | |
| 50 | Chen et al.,2024 | China | Cross-sectional Study | Thyroid Disorders, Iodine  Status and Diabetes Epidemiology (TIDE) study | 69,842 (51.96%) | NA | 20-80 | NA | Objectively | chi-square tests;  Fisher exact tests;near regression models; area under the curve (AUC); multivariate logistic regression;linear regression | age, ethnicity, education,  household income, smoking, Diabetes; Hypertension | |
| 51 | Lin et al.,2024b | the United States | Prospective Cohort Study | National  Health and Nutrition Examination Survey | 15,570 (49.6%) | Hypertension | ≥20 | 8.0 | Objectively | Multivariate Cox proportional  hazards regression model, Weighted restricted cubic spline regression, Log-likelihood ratio test, single-line linear regression model | age, gender, race, education  level, cotinine, blood urea nitrogen, high density lipoprotein cholesterol, total cholesterol, triglyceride, alanine aminotransferase, aspartate transaminase, HbA1c, estimate glomerular filtration rate, family income to poverty, diastolic blood pressure, systolic blood pressure, Charlson Comorbidity Index, chronic obstructive pulmonary disease, Diabetes, arteriosclerotic Cardiovascular Diseases, heavy drinker, smoker, statins, antiplatelet agents, antihypertensive agents and cancer. | |
| 52 | Zhang et al.,2024c | the United States | Cross-sectional Study | National  Health and Nutrition Examination Survey | 29,062 (50.22%) | NA | ≥20 | NA | Objectively | chi-squared test,  weighted linear regression, Multivariate logistic regression, Receiver-operating characteristic (ROC) | age, sex, race, education level, marital status, PIR, smoking status, alcohol consumption, bmi, Hypertension, Diabetes, hyperlipidemia, and Cardiovascular Diseases | |
| 53 | Yu et al.,2022 | China | Cross-sectional Study | China Cardiometabolic  Disease and Cancer Cohort study | 8,866 (64.8%) | BMI≥18.5 kg/m² | ≥40 | NA | Objectively | t test; one-way  ANOVA; χ2 test; Receiver operating characteristic (ROC) curve; logistic regression models | current smoking, current drinking, physical  activity level, Diabetes, Hypertension, Cardiovascular Diseases history and BMI | |
| 54 | Zhang et al.,2023 | China | Prospective Cohort Study | China Health and RetireMalet  Longitudinal Study | 6,152 (52.3%) | NA | ≥45 | 8 | Objectively | Pearson's χ2 tests;  Fisher's exact tests;Cox | age, sex, residence, marital  status, smoking status, drinking status, BMI, SBP, DBP, TC, TG, HDL-C, LDL-C,hypertension,dyslipidemia, kidney disease | |
| 55 | Su et al.,2023 | China | Cross-sectional Study | The Taiwan Biobank | 121,888 (64.07%) | NA | 30-70 | NA | Objectively | Independent t-tests; chi-square test;Kolmogorov–Smirnov test; multivariable logistic regression;Receiver operating characteristic (ROC) curve | age, Diabetes Mellitus,  Hypertension, smoking history, systolic and diastolic BPs, hemoglobin, TGs, total cholesterol, HDL-c, LDL-c, and eGFR | |
| 56 | Liu et al.,2023a | China | Prospective Cohort Study | China Hainan  Centenarian Cohort Study | 1,002 (82.04%) | NA | ≥100 | 5.0 | Objectively | Cox proportional  hazards regression models, restricted cubic spline model, Kaplan–Meier survival curves and log-rank tests | age and sex, by stratifying the  baseline hazard functions by sex and age group (<105y, ≥105y), and included age, education status, marital status, residential type, smoking status, weekly exercise, SBP, HDL cholesterol and Comorbidities status, as regression variables | |
| 57 | Somdee et al.,2023 | Thailand | Cross-sectional Study | Originally designed | 2,520 (61.07%) | NA | 18-60 | NA | Objectively | Kolmogorov- Smirnov test;χ2 test;Mann-Whitney U test;receiver operating characteristic (ROC) curve; area under the curve (AUC) | NA | |
| 58 | Sánchez-Bacaicoa et al., 2023 | Spain | Cross-sectional Study | Originally designed | 1,094 (39.58%) | NA | ≥18 | NA | Objectively | χ2 test; Fisher’s  exact test;Student’s t-test;Mann-Whitney U test;Kolmogorov-Smirnov test; multiple linear regression;multivariate logistic regression;area under the curve;receiver operating characteristic | age ≥ 65 years, current  smokers, Hypertension presence, Diabetes presence, MetS presence, previous CV event, SBP ≥ 140 mmHg, PP ≥ 60 mmHg, TC ≥ 190 mg/dL, LDL ≥ 100 mg/dL, Triglyceride ≥ 200 mg/dL, FPG ≥ 126 mg/dL, antihypertensive, and antidiabetic drugs | |
| 59 | Qin et al.,2023 | China | Cross-sectional Study | China Patient- Centered Evaluative AssessMalet of Cardiac Events Million Persons Project | 101,973 (60.5%) | NA | 35–75 | NA | Objectively | Wilcoxon Rank-Sum Test; Wilcoxon Mann-Whitney test; Kruskal-Wallis H-test; chi-square tests;multicollinearity test;multivariate logistic regression;receiver operating characteristic;area under the curve; C-statistic ; net reclassification index | age, sex, occupation,  residence, marriage, educational status, household income, medical insurance, smoking, drinking, heart rate, total cholesterol, high-density lipoprotein cholesterol, antiplatelet drugs, statins, and lipid-lowering drugs | |
| 60 | Liu et al.,2023b | Japan | Retrospective Cohort Study | the “Dryad” website (https://datadryad.org/) | 15,252 (45.42%) | NA | 18-79 | 6.04 | Objectively | COX regression;Kaplan-Meier curve;smooth curve;piecewise linear regression;Receiver operating characteristic curve;area under the curve | age, sex, Habit of exercise,  ethanol consumption, smoking status, SBP, DBP, ALT, AST, HbA1c, FPG, GGT | |
| 61 | Li et al.,2023a | China | Prospective Cohort Study | China Health and RetireMalet Longitudinal Study | 4,423 (53.90%) | NA | ≥45 | 4 | Objectively | chi-square test; independent samples t-test;logistic regression;receiver operating characteristic curve; area under the curve; Youden index | age, educational levels,  marital status, live place, current smoking, alcohol drinking, activities, exercises, chronic diseases | |
| 62 | Wang et al.,2023 | China | Cross-sectional Study | Originally designed | 12,658 (52.98%) | NA | 18-75 | NA | Objectively | Kolmogorov−Smirnov normality tests; Mann−Whitney U test; χ2 test; ROC curves; Spearman’s rank correlation | NA | |
| 63 | Chung et al.,2023 | China | Prospective Cohort Study | The Taiwan Biobank | 24,346 (65.77%) | NA | 30-70 | 4 | Objectively | Kolmogorov- Smirnov;Levene test;t-test;Mann–Whitney U test;chi-square test;Multivariable logistic regression;Receiver operating characteristic (ROC) curve;areas under curves | age, Hypertension, smoking  and alcohol history, systolic and diastolic BPs, hemoglobin, triglyceride, total cholesterol, HDL-C, LDL-C, eGFR and uric acid,Malestruation status | |
| 64 | Cai et al.,2023 | China | Prospective Cohort Study | the Uygur Research on Obstructive Sleep Apnea in Hypertensive Patients Study | 2,265 (31.21%) | Hypertensive patients with  obstructive sleep apnea | ≥18 | 6.8 | Objectively | Kaplan-Meier method;  the log-rank test;Cox  regression; | age, sex, smoking status, drinking status, diastolic blood pressure, systolic blood pressure, Diabetes, eGFR,  total cholesterol, triglyceride, HDL-C, LDL-C, fasting plasma glucose, apnea hypopnea index,use of antidiabetic drugs, aspirins, statins, obstructive sleep apnea therapy, antihypertensive  drugs | |
| 65 | Anto et al.,2023 | Ghana | Cross-sectional Study | Originally designed | 1,288 (53.34%) | NA | 18-80 | NA | Objectively | Kolmogorov– Smirnov  test;chi-square test; Student’s t-test ; Mann–Whitney U test;logistic regression;receiver operating characteristic curve | age, marital status, alcohol  intake and exercise level of participants | |
| 66 | Göçer et al.,2022 | Turkey | Cross-sectional Study | Originally designed | 229 (84.7%) | NA | 18-65 | NA | Objectively | Kolmogorov-Smirnov/Shapiro-Wilk tests; t-test;e Mann-Whitney U-test; chi square test; Spearmans test; ROC curve | NA | |
| 67 | Wu et al.,2022a | China | Cross-sectional Study | Originally designed | 76,915 (40.18%) | NA | ≥18 | NA | Objectively | chi-square test; Multiple logistic regression; Receiver operating characteristic;area under the curve | age, Hypertension, Diabetes,  Hypertension and Diabetes family history, smoking, alcohol consumption | |
| 68 | Wu et al.,2022b | the United States | Cross-sectional Study | National Health and  Nutrition Examination Survey | 45,853 (49.92%) | NA | 18-79 | NA | Objectively | independent t-tests;  chi-square test; Mann–Whitney U test; Multivariable logistic regression;z-standardized;ROC curves;Restricted cubic spline analysis; | age, sex, race/ethnicity,  education, smoking, drinking, Diabetes, and eGFR | |
| 69 | Wu et al.,2022c | Japan | Retrospective Cohort Study | Dryad data  package:www. Datadryad.org | 15,310 (45.37%) | NA | ≥18 | 5.39 | Objectively | one-way ANOVA; Kruskal–Wallis;chi-square test;Survival estimates; Cox | age, gender,  ALT; AST; DBP; FBG; HbA1c hemoglobin A1c; HDL-C;SBP systolic blood pressure; TC total cholesterol; TG; ethanol consumption, smoking status, exercise, fatty liver | |
| 70 | Sekgala et al.,2022 | South Africa | Cross-sectional Study | Originally designed | 185 (0%) | NA | ≥20 | NA | Objectively | t-test; chi-square  test; ROC curve;area under the curve;logistic regression; | age, race, marital status,  driving experience in years, education, smoking, alcohol intake , physical activity | |
| 71 | Oliveira et al.,2022 | Brazil | Prospective Cohort Study | Originally designed | 498 (25.60%) | NA | ≥18 | 10 | Objectively | Kolmogorov– Smirnovtest;t‐test;chi‐squared test;logistic regression;Receiver Operational Characteristics (ROC) curves;area under the curve; | Age, Sex, SBP, HDL‐c  (high‐density lipoprotein) | |
| 72 | Li et al.,2022 | the United States | Cross-sectional Study | National  Health and Nutrition Examination Survey | 4195 (50.68%) | NA | ≥18 | NA | Objectively | Chi-square test;  Weighted multiple linear or logistic regression analyses; the area under receiver operating characteristic (ROC) curves | age, race/ethnicity, education level, marital status, uric acid, lipid lowering medication use and antihypertensive medication use | |
| 73 | Lee et al.,2022a | China | Cross-sectional Study | Originally designed | 2,801 (46.48%) | NA | 18-81 | NA | Objectively | ROC;multiple nonparametric Z-test;areas under the curve (AUC);multivariable logistic regression; | Age, rest heart rate, arteriosclerosis, lifestyle (smoking and exercise status), cardiorespiratory fitness | |
| 74 | Lee et al.,2022b | China | Prospective Cohort Study | Ministry of Health and  Welfare in Taiwan established the Taiwan Biobank | 21,466 (67.86%) | NA | 30-70 | 4 | Objectively | independent t-test; Chi-square test;multivariable logistic regression;Receiver operating characteristic (ROC) curves;areas under the ROC curves (AUCs) | age, Diabetes, smoking and  alcohol history, regular exercise habits, fasting glucose, hemoglobin, triglyceride, total cholesterol, HDL-cholesterol, LDL-cholesterol, eGFR, uric acid | |
| 75 | Kahaer et al.,2022 | China | Cross-sectional Study | Originally designed | 2,243 (27.95%) | NA | 20-69 | NA | Objectively | independent sample  t-test;Mann–Whitney U-test;Kruskal–Wallis H-test;Partial correlation analysis;Logistic analysis;ROC curve; | age, SBP, DBP, BUN, Cre, TC, LDL-C | |
| 76 | Duan et al.,2022 | China | Cross-sectional Study | Originally designed | 1,425 (58.74%) | NA | ≥18 | NA | Objectively | Kolmogorov– Smirnov test;Levene test ; one-way ANOVA;Student’s t-test, chi-square test; Mann‐Whitney U test;logistic regression;Receiver operating characteristic (ROC) analysis;DeLong test | systolic blood pressure,  diastolic blood pressure, total cholesterol and eGFR | |
| 77 | Cai et al.,2022 | China | Prospective Cohort Study | China Health and  RetireMalet Longitudinal Study | 1,815 (46.99%) | NA | ≥45 | 4 | Objectively | t test ; chi-square  test;Logistic regression;the area under the receiver operating characteristic curves (AUROC);Multivariable linear regression analysis; | age, sex, body mass index, history of smoking and  drinking, diseases tatus (Hypertension, Dyslipidemia, and Cardiovascular Diseases), systolic blood pressure, hemoglobin A1c, TC/high-density lipoprotein cholesterol, uric acid, C-reactive protein, walking speed at baseline | |
| 78 | Zhao et al.,2021 | Japan | Prospective Cohort Study | NAfld in the Gifu Area,  Longitudinal Analysis study | 15,464 (45.49%) | NA | 18-79 | 10 | Objectively | one-way ANOVA;  Kruskal–Wallis H test; χ2 test;Cox regression;eceiver operating characteristic (ROC) curve | age, alcohol intake, smoking  status, fatty liver, SBP, FPG, HbA1c, HDL-C, TG, and TC | |
| 79 | Sheng et al.,2021 | Japan | Cross-sectional Study | NAfld in the Gifu Area,  Longitudinal Analysis study | 14,281 (48.11%) | NA | ≥18 | NA | Objectively | Pearson χ2 test;Shapiro–Wilk test;nonparametric  test; Student’s t-test;Multiple logistic regression;ROC curve;Area under the curve (AUC) | age, habit of exercise, GGT,  TC, HDL-C, HbA1c, smoking status, drinking status and DBP | |
| 80 | Liu et al.,2021 | China | Prospective Cohort Study | Originally designed | 6,990 (40.4%) | Hypertensive | ≥18 | 3.1 | Objectively | Student's t-test;Kruskal-Wallis  rank-sum test;chi-square test; Cox regression;area under receiver operating characteristic (ROC) curves; | sex, age, smoking status,  drinking status,TG, TC, HDL, LDL, SBP, DBP | |
| 81 | Zhou et al.,2020 | China | Cross-sectional Study | Originally designed | 1,603 (40.7%) | Maintenance  Hemodialysis | ≥18 | NA | Objectively | one-way analysis of  variance (ANOVA) tests;Mann–Whitney U tests;Chi-square test;binary logistic regression;Receiver operating characteristic (ROC);areas under the ROC curves (AUCs) | age, sex, educational status,  history of smoking | |
| 82 | Saadati et al.,2020 | the United States | Prospective Cohort Study | Atherosclerosis Risk  in Communities | 12,725 (54.5%) | Diabetic or  Non‐diabetic | 45-75 | 27 | Objectively | χ ^2^ test; t‐test ; super learner approach;sensitivity analyses;multivariate logistic regression | demographic variables,  family history, behavioural variables and biologic variables plus waist circumference, hip circumference | |
| 83 | Baveicy et al.,2020 | Iran | Cross-sectional Study | Ravansar Non-Communicable Diseases cohort study | 8,790 (47.77%) | NA | 35-65 | NA | Objectively | Chi-square; Fisher  exact test;Kolmogorov–Smirnov test;t-test ; Mann–Whitney non-parametric test;logistic models;Hosmer and Lemeshow test | age, smoking status,  alcohol intake for Male;  smoking status, alcohol intake, Maleopause for Female | |
| 84 | Suliga et al.,2019 | Poland | Cross-sectional Study | Polish-Norwegian  Study project | 12,328 (66.79%) | NA | 37-66 | NA | Objectively | U Mann–Whitney  tests; Chi-squared tests;Multivariate logistic regression;Receiver Operating Characteristic;area under the curve | age, education, physical  activity, alcohol consumption (continuous variables), and smoking status | |
| 85 | Liu et al.,2019 | China | Cross-sectional Study | Originally designed | 75,560 (53.4%) | Non-obese | ≥18 | NA | Objectively | nonparametric test;  χ2 test;t-test;logistic regression analysis;Receiver operating characteristic (ROC) ;rea under the ROC curves (AUC) | age, sex, smoking status,  and medication (antihypertensive agents, statins, low-dose aspirin, and hypoglycemic agents) | |
| 86 | Li et al.,2019 | China | Cross-sectional Study | Originally designed | 1,442 (58.74%) | Obese and  Overweight | ≥35 | NA | Objectively | Student's t test; χ2 test;Logistic regression;receiver operating characteristic (AROC) curves | abdominal circumference,  age,family income, educational level, smoking, drinking, physical activity,SBP, DBP, UA, TG, TC, HDL-C, LDL-C, FPG | |
| 87 | Zhao, et al.,2018 | China | Cross-sectional Study | Originally designed | 15,078 (54.57%) | NA | 39-58 | NA | Objectively | Kolmogorov– Smirnov test;Kruskal–Wallis test;Spearman's rank test;Logistic regression;area under the receiver-operating characteristic curve (AUROC) | age | |
| 88 | Zhang et al.,2018 | China | Cross-sectional Study | Originally designed | 59,029 (38.85%) | NA | 18-80 | NA | Objectively | t-test; chi-square  test; Receiver Operating Characteristic (ROC) curve;area under the curve;logistic regression;Pearson and partial correlation analyses | age | |
| 89 | Yang et al.,2018 | China | Prospective Cohort Study | Dongfeng-Tongji (DFTJ)  cohort study | 9,962 (39.79%) | NA | ≥60 | 4.6 | Objectively | Cox proportional  hazard model;Receiver operating characteristic (ROC); areas under the ROC curves (AUC) | age, smoking, drinking,  physical activity, education level,Hypertension, hyperlipidemia (except VAI), FBG (except VAI) , family history of Type 2 Diabetes | |
| 90 | Choi et al.,2018 | South Korea | Prospective Cohort Study | Korean Genome and  Epidemiology Study on Atherosclerosis Risk of Rural Areas in the Korean General Population | 1,718 (63.33%) | NA | 39–72 | 2.8 | Objectively | chi-square test; two- sample t-test;logistic regression;area under the receiver-operating characteristic curve (AUC) | age, sex, alcohol  consumption (current), smoking status (current) and physical exercise | |
| 91 | Zhang et al.,2016a | China | Cross-sectional Study | Originally designed | 11,345 (53.7%) | NA | ≥35 | NA | Objectively | t-test, ANOVA,  non-parametric test; χ^2^-test;multiple logistic regression;area under curve (AUC) | age, race, family income,  education, smoking, alcohol status, Diabetes and Hypertension | |
| 92 | Motamed et al.,2016 | Iran | Cross-sectional Study | Originally designed | 4,872 (44.1%) | NA | 18-74 | NA | Objectively | univariate and multivariate regression analyses | age,TG, HDL, HOMA, and MAP | |
| 93 | Tian et al.,2016 | China | Cross-sectional Study | the 2009 wave of the China Health and Nutrition Survey | 8,126 (53.5%) | NA | 18-85 | NA | Objectively | t test; χ^2^ test; Receiver-operating characteristic (ROC) analyses; logistic regression models | age and gender | |
| Abbreviations: NA, not applicable; NAFLD, Non-alcoholic fatty liver disease; MetS, Metabolic Syndrome; CKD, Chronic Kidney Disease; AUC, Area Under the Curve; MAFLD, Metabolic dysfunction-associated fatty liver disease; ROC, Receiver operating characteristic; TG, triglyceride levels; HDL, high density lipoprotein levels; MAP, mean arterial pressure; FPG, fasting plasma glucose; TC, total cholesterol; HDL, high-density lipoprotein; LDL, low-density lipoprotein; HbA1c, hemoglobin A1c; PIR, poverty-to-income ratio; SBP, systolic blood pressure; DBP, diastolic blood pressure; ALB, albumin; ALT, alanine aminotransferase; ALP, alkaline phosphatase; TBIL, total bilirubin; Hb, hemoglobin; RBC, red blood cell count; WBC, white blood cell count; PLT, platelet count; TyG, triglyceride-glucose index; UA, uric acid; BUN, blood urea nitrogen; SCr, serum creatinine; ACR, albumin-to-creatinine ratio; eGFR, estimated glomerular filtration rate; HDL-C, high-density lipoprotein; LDL-C, low-density lipoprotein; CRP, C-reactive protein; BUN, blood urea nitrogen; SUA, serum uric acid; BMI, body mass index; WC, waist circumference; WHtR, waist-to-height ratio; BRI, body roundness index; CI, conicity index; LAP, lipid accumulation product index; VAI, visceral adiposity index; CVAI, Chinese visceral adiposity index; CAD, coronary arterial disease; CUN-BAE, Clínica Universidad de Navarra-Body Adiposity Estimator; CV, cardiovascular; PP, pulse pressure; GGT, gamma-glutamyl transferase; | | | | | | | | | | | |  |

| Suppl. Table 2 Measures of the studies included in the meta-analysis. | | | | | | | | | | | | | | |
| --- | --- | --- | --- | --- | --- | --- | --- | --- | --- | --- | --- | --- | --- | --- |
| Study  ID | Study outcome | Outcome assessment | BRI | BMI | WC | WHtR | Others | | | | | | | |
|  |  |  |  |  |  |  |  |  |  |  |  |  |  |  |
| 1 | Type 2 Diabetes | HR (95%CI) | 1.27 (1.20 , 1.35) |  |  |  |  |  |  |  |  |  |  |  |
| 2 | Diabetes Mellitus | HR (95%CI); AUC (95%CI) | HR 1.08  (1.06, 1.11)  AUC 0.692  (0.672,0.711) |  |  | HR 1.14  (1.06, 1.22)  AUC 0.668  (0.646,0.689) | VAI HR 1.15  (1.12, 1.18)  AUC 0.677  (0.656,0.698) | ABSI HR 1.16  (1.06, 1.29)  AUC 0.694  (0.674,0.713) | LAP HR 1.74 (1.63, 1.86)  AUC 0.752  (0.734,0.770) |  |  |  |  |  |
| 3 | CVD; Heart disease; Stroke | OR (95%CI) | CVD  1.14 (1.04,1.26) Heart disease 1.08 (0.98,1.20) Stroke 1.17 (1.02,1.35) | CVD  1.09 (0.98,1.21) | CVD  1.09 (0.98,1.21) |  | CVAI CVD  1.14 (1.03,1.25) |  |  |  |  |  |  |  |
| 4 | HTN; Diabetes Mellitus; Dyslipidemia | AUC (95%CI) | HTN 0.568  (0.556, 0.579) Diabetes 0.554  (0.533, 0.575) Dyslipidemia 0.597  (0.588, 0.607) | HTN 0.606  (0.595, 0.617) Diabetes 0.573  (0.552, 0.594) Dyslipidemia 0.612  (0.603, 0.621) |  | HTN 0.606 (0.595, 0.617) Diabetes 0.573 (0.552, 0.594) Dyslipidemia 0.625  (0.616,0.635) | ABSI HTN 0.532  (0.520, 0.544) Diabetes 0.530 (0.508, 0.552) Dyslipidemia 0.581  (0.572, 0.591) | BF HTN 0.600  (0.589,0.611) Diabetes 0.571  (0.550,0.593) Dyslipidemia 0.600  (0.590,0.609) | WHR HTN 0.587  (0.576, 0.599) Diabetes 0.566  (0.545, 0.588) Dyslipidemia 0.618  (0.609, 0.628) | BAI HTN 0.582  (0.571, 0.593)  Diabetes 0.544 (0.522, 0.566) Dyslipidemia 0.576  (0.567,0.586) | CI HTN  0.573  (0.561, 0.584) Diabetes 0.556 (0.535, 0.577) Dyslipidemia 0.615  (0.606, 0.625) |  |  |  |
| 5 | HTN | HR (95%CI) AUC (95%CI) | HR 1.501 (1.193, 1.887) AUC 0.663 (0.614, 0.711) | HR 1.147  (1.075, 1.224) AUC 0.672  (0.625, 0.719) | HR 1.051 (1.025, 1.077) AUC 0.666 (0.617, 0.715) | HR 2.168 (1.394, 3.370) AUC 0.663 (0.614 , 0.711) | ABSI HR 0.930  (0.540, 1.601) AUC 0.537  (0.490, 0.585) |  |  |  |  |  |  |  |
| 6 | NAFLD | OR (95%CI); AUC (95%CI) | OR 0.09 (0.03, 0.25)  AUC Male 0.75 (0.717,0.782) Female 0.702 (0.684, 0.72) | OR 1.14 (1.06,1.24)  AUC Male0.754 (0.722, 0.786) Female0.701  (0.683, 0.719) |  | OR 16.3  (4.27, 62.24)  AUC Male 0.75 (0.717,0.782) Female 0.702 (0.684, 0.72) | VAI OR 1.03 (1.01, 1.04)  AUC Male 0.621  (0.578, 0.663) Female 0.622 (0.602, 0.64) | ABSI OR 1.93 (0.02,150.05) AUC Male0.53 (0.489, 0.57) Female0.505  (0.485,0.526) | WHR OR 1.35 (1.12, 1.64)  AUC Male 0.727 ( 0.694, 0.76) Female 0.64 (0.621, 0.659) |  |  |  |  |  |
| 7 | Stroke | HR (95% CI) | 1.158  (1.158,1.15) |  |  |  |  |  |  |  |  |  |  |  |
| 8 | MetS | OR (95%CI) | Male 4.74  (1.91, 11.78) Female 3.01  (1.81, 5.00) | Male 15.80  (1.72, 144.85) | Male 1.18 (1.08, 1.29) Female 1.18 (1.13, 1.23) | Male 5.38  (1.15, 2.51) Female 6.87  (6.21, 7.59) | HC Male 2.15 (1.02, 4.52) Female 1.15 (1.05, 1.27) | VAI Male 8.67  (2.23, 29.36) Female 13.09  (3.03, 56.62) | ABSI Male 7.15 (5.44, 9.41) Female 5.97  (2.18, 16.39) |  |  |  |  |  |
| 9 | CHD | RR (95%CI) | 1.00 (0.84,1.20) Male 1.05 (0.85,1.30) Female 1.06 (0.75,1.48) | 1.15 (1.00,1.34)  Male 1.17 (0.97,1.40)  Female 1.19 (0.94,1.52) | 1.12 (0.97,1.31)  Male 1.16 (0.99,1.36)  Female 1.01 (0.73,1.73) | 0.92 (0.73,1.17) Male 0.74 (0.56,0.98) Female 1.78 (1.09,2.92) | ABSI 1.19 (0.97,1.45)  Male 1.23 (0.95,1.60)  Female 1.13 (0.83,1.55) | WHR 0.93 (0.74,1.16) Male 0.83 (0.59,1.19) Female1.00 (0.73,1.38) |  |  |  |  |  |  |
| 10 | CVD | OR (95%CI) | 1.005  (1.002 ,1.009) |  |  |  |  |  |  |  |  |  |  |  |
| 11 | Hyperuricemia | OR (95%CI); AUC (95%CI) | OR 1.204 (1.173, 1.236)  AUC 0.619  (0.608,0.631) Male 0.608 (0.590, 0.627) Female 0.618 (0.602, 0.634) | OR 1.106 (1.090, 1.121)  AUC 0.614  (0.602, 0.626) Male 0.604 (0.586,0.622) Female 0.620 (0.604, 0.636) |  | OR 1.056  (1.047,1.064) AUC 0.621  (0.609,0.633) Male 0.605  (0.587,0.623) Female 0.614 (0.597, 0.630) | ABSI OR 1.083  (1.002, 1.171) AUC 0.541 (0.529, 0.553) Male 0.538 (0.520, 0.557) Female 0.531 (0.514, 0.547) | CMI OR 1.606  (1.488,1.735) AUC 0.645 (0.633,0.656) Male 0.620 (0.602,0.638) Female 0.661  (0.646,0.677) | WWI OR 1.185  (1.120, 1.253) AUC 0.580  (0.568, 0.592) Male 0.565  (0.546, 0.583) Female 0.561  (0.545, 0.578) |  |  |  |  |  |
| 12 | Stroke; Heart disease; CVD | OR (95%CI) | Stroke 1.32  (1.15, 1.52) Heart disease 1.19  (1.07, 1.32) CVD 1.25  (1.14, 1.37) | Stroke 1.27 (1.12, 1.43) Heart disease 1.17 (1.06, 1.29) CVD 1.22 (1.12, 1.33) | Stroke 1.32 (1.13, 1.53) Heart disease 1.23 (1.10, 1.37) CVD 1.30 (1.18, 1.42) | Stroke 1.36  (1.17, 1.58) Heart disease 1.18  (1.06, 1.32) CVD 1.25  (1.14, 1.38) | CVAI Stroke 1.42 (1.21,1.66) Heart disease  1.21 (1.08, 1.36) CVD 1.30(1.17,1.44) | LAP Stroke  1.14  (1.01, 1.28)  Heart disease 1.01  (0.90, 1.12) CVD1.10  (1.01, 1.20) | TyG Stroke  1.25 (1.06, 1.48) Heart disease 1.02 (0.90, 1.16) CVD 1.11 (1.00, 1.24) | TyG-BMI Stroke 1.35  (1.18, 1.55) Heart disease  1.17  (1.05, 1.30) CVD1.26  (1.14, 1.38) | TyG-WC Stroke  1.41 (1.20, 1.66) Heart disease 1.21 (1.07, 1.36) CVD 1.32 (1.19,1.47) | TyG-WHtR Stroke 1.48 (1.25, 1.74) Heart disease 1.17 (1.04, 1.33) CVD 1.29 (1.16,1.44) |  |  |
| 13 | Hyperuricemia | AUC (95%CI) | Male 0.599  (0.587,0.612)  Female 0.716  (0.704,0.729) | Male 0.642  (0.630, 0.654)   Female 0.720  (0.697, 0.744) | Male 0.638  (0.626, 0.651)   Female 0.721  (0.697, 0.745) | Male 0.626  (0.614,0.638)  Female 0.728 (0.704,0.751) | LAP Male 0.694  (0.682, 0.705) Female 0.767  (0.755, 0.779) | TyG Male 0.661  (0.649,0.673) Female 0.746  (0.734, 0.75) |  |  |  |  |  |  |
| 14 | CVD; Stroke | OR (95%CI) | CVD 1.11 (1.07,1.15) Male:1.13 (1.07, 1.19) Female :1.10 (1.05, 1.15) Stroke 1.02  (0.96, 1.09) |  |  |  |  |  |  |  |  |  |  |  |
| 15 | Stroke | OR (95%CI) | 1.06  (1.01,1.11) |  |  |  |  |  |  |  |  |  |  |  |
| 16 | MetS; Dyslipidemia; Diabetes Mellitus; HTN | AUC (95%CI) | MetS 0.73 (0.7,0.75) Male: 0.724 (0.705,0.74) Female:  0.667 (0.61,0.724) Dyslipidemia 0.54  (0.51,0.57) Male: 0.539 (0.51, 0.56) Female:0.539 (0.51, 0.56) Diabetes Mellitus  0.68  (0.66, 0.69) Male: 0.665 (0.648,0.681) Female:0.686 (0.638,0.73) HTN 0.61 (0.6,0.63) Male: 0.609 (0.59, 0.62)  Female:  0.661 (0.607,0.71) | MetS  0.78 (0.76, 0.80) Male: 0.766 (0.748, 0.783) Female: 0.648 (0.587, 0.706) Dyslipidemia  0.53 (0.5, 0.56) Male:0.528 (0.5, 0.557) Female: 0.568 (0.476,0.657) Diabetes Mellitus  0.69 (0.67, 0.71) Male: 0.682(0.665, 0.698) Female: 0.657 (0.608, 0.704) HTN  0.61 (0.58, 0.63) Male: 0.601 (0.583, 0.62) Female:0.627 (0.573, 0.679) | MetS  0.75 (0.73, 0.76) Male:0.728 (0.71, 0.745) Female: 0.659 (0.598, 0.716) Dyslipidemia  0.53 (0.5, 0.56) Male: 0.529 (0.501, 0.557) Female:0.55 (0.458, 0.64) Diabetes Mellitus 0.67 (0.66, 0.69) Male: 0.664 (0.647, 0.68)  Female: 0.666 (0.617, 0.712) HTN  0.60 (0.58, 0.62) Male: 0.597 (0.579, 0.612) Female:0.649 (0.595, 0.7) | MetS  0.73  (0.7,0.75) Male:0.724 (0.705,0.742) Female:0.665 (0.604,0.722)  Dyslipidemia  0.54 (0.51,0.57) Male: 0.539 (0.51, 0.569) Female:0.534 (0.441,0.624) Diabetes Mellitus  0.68 (0.66,0.69) Male: 0.665 (0.648,0.681) Female:0.685 (0.637,0.731) HTN 0.61 (0.59,0.63) Male: 0.609 (0.59, 0.63) Female: 0.66 (0.606,0.710) | WHR MetS  0.66 (0.64, 0.68) Male: 0.639 (0.619, 0.658) Female: 0.634 (0.572, 0.692) Dyslipidemia  0.50 (0.47, 0.53) Male: 0.5 (0.472, 0.528) Female: 0.577 (0.485, 0.665) Diabetes Mellitus  0.65 (0.63, 0.0.66) Male:0.628 (0.61, 0.645) Female: 0.679 (0.631, 0.725) HTN  0.59 (0.57, 0.61) Male: 0.595 (0.576, 0.613) Female:0.624 (0.57, 0.677) | ABSI MetS  0.51 (0.49,0.53) Male: 0.5 (0.48, 0.52) Female: 0.52 (0.457,0.582) Dyslipidemia  0.52 (0.49,0.55) Male: 0.518 (0.489,0.546) Female:0.511 (0.419,0.602) Diabetes Mellitus  0.50 (0.49,0.53) Male: 0.501 (0.483,0.518) Female:0.543 (0.493,0.593) HTN  0.507 (0.487,0.528) Male: 0.524 (0.505,0.543) Female:0.534 (0.479,0.588) | AVI MetS 0.75 (0.73, 0.77) Male: 0.73 (0.712, 0.748)  Female: 0.659 (0.698, 0.716) Dyslipidemia  0.52 (0.5, 0.56) Male:0.53 (0.501, 0.558) Female: 0.546 (0.454, 0.636) Diabetes Mellitus  0.67 (0.64, 0.69) Male:0.663 (0.64, 0.68) Female: 0.665 (0.616, 0.711) HTN  0.60 (0.58, 0.62) Male:0.597 (0.578, 0.616) Female:0.648 (0.594, 0.699) | HC MetS  0.72 (0.7,0.74) Male: 0.712 (0.694,0.731) Female:0.601 (0.538,0.661) Dyslipidemia  0.54 (0.51,0.57) Male: 0.545 (0.516,0.573) Female:0.527 (0.435,0.618) Diabetes Mellitus 0.63 (0.61,0.65) Male:0.624 (0.607,0.641) Female:0.567 (0.516,0.616) HTN  0.57 (0.55,0.59) Male:0.561 (0.542, 0.58)  Female:0.586 (0.531, 0.64) | BAI MetS  0.67 (0.65, 0.69) Male: 0.683 (0.664, 0.702) Female: 0.603 (0.54, 0.663) Dyslipidemia 0.56 (0.53, 0.59) Male: 0.556 (0.527, 0.584)  Female: 0.509 (0.417, 0.6) Diabetes Mellitus  0.619 (0.6, 0.64) Male:0.612 (0.595, 0.629)  Female: 0.594 (0.544, 0.643) HTN  0.60 (0.58, 0.62) Male: 0.58 (0.561, 0.599) Female: 0.605 (0.551, 0.658) | WWI MetS  0.57 (0.55, 0.59) Male:0.587 (0.567, 0.607)  Female:0.603 (0.541, 0.663) Dyslipidemia  0.54 (0.51, 0.57) Male: 0.532 (0.504, 0.561) Female: 0.523 (0.431, 0.614) Diabetes Mellitus  0.58 (0.56, 0.59) Male: 0.571 (0.554, 0.589)  Female: 0.623 (0.573, 0.671) HTN  0.57 (0.55, 0.59) Male: 0.569 (0.55, 0.588) Female: 0.607 (0.552, 0.66) |  |  |
| 17 | MetS | AUC (95%CI) | Male: 0.906 (0.895,0.917) Female: 0.833  (0.820,0.846) |  |  |  |  |  |  |  |  |  |  |  |
| 18 | All-cause Mortality | HR (95%CI) | 1.01(0.99,1.04) Male:1.06  (1.01, 1.11) Female:0.99  (0.96, 1.03) HTN:Yes:1.04  (1.00, 1.08) No:0.97 (0.93,1.01) Race Non-Hispanic White:1.01  (0.98, 1.05) Non-Hispanic Black:1.02  (0.97,1.08) Mexican American:1.01  (0.94, 1.09) |  |  |  |  |  |  |  |  |  |  |  |
| 19 | All-cause and  Cardiovascular Mortality | HR (95%CI) | All-cause Mortality 1.04  (1.01,1.07) Cardiovascular Mortality 1.08  (1.02,1.14) |  |  |  |  |  |  |  |  |  |  |  |
| 20 | All-cause and  Cardiovascular Mortality | HR (95%CI) | All-cause Mortality 1.16  (1.07, 1.27) Gender Male:1.13  (1.02, 1.24) Female:1.22  (1.04, 1.43) HTN Yes:1.17  (1.06, 1.31) No:1.10 (0.91,1.32) Cardiovascular Mortality 1.27  (1.13, 1.42) Male:1.15  (0.99, 1.34) Female:1.56  (1.15, 2.10) HTN Yes:1.27  (1.05,1.55) No:1.20  (0.90, 1.61) | All-cause Mortality 1.03  (1.01, 1.06)   Cardiovascular Mortality 1.05  (1.00, 1.10) | All-cause Mortality  1.03 (1.00, 1.05)  Cardiovascular Mortality 1.01 (0.96, 1.07) |  | CMI All-cause Mortality 0.98  (0.87, 1.11)  Cardiovascular Mortality  0.86 (0.70, 1.05) | LAP All-cause Mortality 1.00  (0.99, 1.01)  Cardiovascular Mortality 1.01  (1.00, 1.03) | VAI All-cause Mortality 0.99 (0.95, 1.03)  Cardiovascular Mortality 0.95 (0.88, 1.02) | TyG All-cause Mortality 1.23  (0.91, 1.67)  Cardiovascular Mortality 0.75  (0.41, 1.37) |  |  |  |  |
| 21 | Cardiovascular Mortality | HR (95%CI) | 1.12  (1.06, 1.18) | 1.11  (1.06, 1.18) | 1.19 (1.13, 1.26) |  | ABSI 1.13  (1.07, 1.19) |  |  |  |  |  |  |  |
| 22 | All-cause Mortality | HR (95% CI) | 0.89  (0.83, 0.95) Male:0.99 (0.85, 1.14) Female:0.86 (0.76, 0.94) HTN: Yes:0.88  (0.80, 0.96) No:0.92 (0.82,1.02) Race Non-Hispanic White: 0.89  (0.82, 0.97) Non-Hispanic Black: 0.87  (0.78, 0.98) Mexican American: 0.94  (0.79, 1.11) |  |  |  |  |  |  |  |  |  |  |  |
| 23 | CKD | OR (95%CI) AUC (95%CI) | OR 1.29  (1.01, 1.64) AUC  0.599  (0.521, 0.676) | AUC  0.487  (0.406, 0.568) |  | OR 1.31  (1.00, 1.72) AUC 0.599  (0.521,0.676) | CVAI OR 1.29 (0.98, 1.69) AUC  0.600  (0.523, 0.676) | ABSI OR 1.38  (1.01, 1.89) AUC 0.678  (0.613,0.744) | CI OR 1.45 (1.05, 2.01) AUC  0.663  (0.593, 0.733) |  |  |  |  |  |
| 24 | CKD | OR (95%CI) | 0.989 (0.960,1.018) | 1.012 (1.001, 1.023) | 1.003  (0.999, 1.007) | 0.775  (0.395,1.521) | ABSI 0.425  (0.205, 0.882) | WHR  0.534 (0.276,1.034) |  |  |  |  |  |  |
| 25 | CKD | OR (95%CI) | Total 1.109 (1.04,1.183) Male 1.18  (1.09, 1.29) Female 1.03  (0.97, 1.10) |  |  |  |  |  |  |  |  |  |  |  |
| 26 | CKD | OR (95%CI) AUC (95%CI) | OR 1.30  (1.23, 1.38) AUC 0.610  (0.619,0.628) | OR 1.25 (1.18, 1.33) AUC 0.541  (0.550, 0.560) | OR 1.27 (1.20, 1.35) AUC 0.586  (0.595, 0.605) | OR 1.30  (1.23, 1.38) AUC 0.609  (0.618,0.628) | ABSI OR 1.17 (1.11, 1.23) AUC 0.655  (0.664, 0.673) | CI OR 1.29  (1.22, 1.36) AUC 0.657  (0.665,0.674) |  |  |  |  |  |  |
| 27 | CKD | OR (95%CI) | Male 1.04  (1.00, 1.07) Female 1.03  (1.00, 1.05) |  |  |  |  |  |  |  |  |  |  |  |
| 28 | MAFLD | AUC (95%CI) | 0.803  (0.767,0.840) | 0.792  (0.794, 0.830) |  | 0.803  (0.767,0.840) | WHR 0.671  ( 0.626, 0.716) | CI 0.687  (0.642,0.732) | ABSI 0.586  ( 0.538, 0.635) | AVI 0.805  (0.770,0.841) | TyG 0.675  (0.631, 0.718) | TyG-WC 0.826  (0.792, 0.860) | TyG-BMI 0.820  ( 0.785, 0.855 ) | HSI 0.783  (0.743, 0.822) |
| 29 | HTN | OR (95%CI) AUC (95%CI) | OR Male  1.58 (1.49,1.68) Female  1.39 (1.33,1.46) AUC Male  0.651 (0.635,0.668) Female  0.664 (0.649,0.679) | OR Male  1.17 (1.15,1.20) Female  1.13 (1.11,1.15)  AUC Male  0.635 (0.618,0.652) Female  0.614 (0.599,0.630) | OR Male  1.06 (1.05,1.07) Female 1.05 (1.04,1.06)  AUC Male  0.648 (0.631,0.665) Female  0.648 (0.633,0.664) | AUC Male  0.651 (0.635,0.668) Female  0.664 (0.649,0.679) | CVAI OR Male  1.01 (1.01,1.01) Female  1.01 (1.01,1.01) AUC Male  0.660 (0.643,0.676) Female  0.699 (0.685,0.713) | VAI OR Male 1.05 (1.03,1.07) Female  1.04 (1.03,1.05) AUC Male  0.582 (0.565,0.600) Female  0.604 (0.588,0.619) | ABSI OR Male  1.22 (1.07,1.38) Female  1.12 (1.00,1.24) AUC Male 0.566 (0.549,0.584) Female  0.589 (0.573,0.604) | LAP OR Male 1.01 (1.01,1.02) Female  1.01 (1.01,1.01) AUC Male0.632 (0.615,0.649) Female0.646 (0.631,0.662) | CI OR Male  36.36 (15.98,82.72) Female  13.72 (6.86,27.45) AUC Male 0.615 (0.598,0.632) Female0.631 (0.616,0.646) | TyG OR Male  1.53 (1.38,1.70) Female  1.53 (1.38,1.69)  AUC Male  0.586 (0.569,0.603) Female  0.610 (0.594,0.625) | TyG-BMI OR Male  1.02 (1.01,1.02) Female  1.01 (1.01,1.01)  AUC Male  0.640 (0.623,0.657) Female  0.636 (0.621,0.651) | TyG-WC OR Male  1.01 (1.00,1.01) Female  1.00 (1.00,1.01)  AUC Male  0.648 (0.631,0.665) Female 0.663 (0.648,0.678) |
| 30 | NAFLD | OR (95%CI) | 1.41 (1.34, 1.48) Male 1.51  (1.37,1.66) Female 1.39  (1.31,1.48) |  |  |  |  |  |  |  |  |  |  |  |
| 31 | Type 2 Diabetes | AUC (95%CI) | AUC Male: 0.632  (0.609,0.654)  Female:0.611(0.591,0.632) | AUC Male:0.613  (0.590,0.636)  Female:0.585  (0.564,0.606) | AUC Male:0.625  (0.602,0.648)  Female:0.610  ( 0.590,0.631) | AUC Male: 0.632  (0.609,0.654)  Female: 0.611  (0.591,0.632) | CVAI AUC Male:0.654(0.631,0.676)  Female:0.672  (0.652,0.691) | ABSI AUC Male: 0.571  (0.549,0.594)  Female: 0.561  (0.540,0.581) | VAI AUC Male: 0.636  (0.612,0.659)  Female:0.659  (0.639,0.679) | LAP AUC Male: 0.656  (0.633,0.679)  Female: 0.669  (0.649,0.689) | CI AUC Male: 0.609  (0.587,0.632)  Female: 0.589  (0.569,0.609) | TyG AUC Male:0.780  (0.761,0.799)  Female:0.782  (0.764,0.799) | TyG-WC AUC Male:0.700  (0.679,0.722 )  Female:0.686  ( 0.667,0.705) | TyG-WHtR AUC Male: 0.734  (0.713,0.754)  Female:0.723  (0.704,0.741) |
| 32 | HTN | OR (95%CI) | 1.32  (1.26, 1.38) | 1.32 (1.26, 1.38) | 1.33 (1.27, 1.40) | 1.35  (1.28, 1.41) | ABSI 1.09  (1.04, 1.16) |  |  |  |  |  |  |  |
| 33 | HTN | HR (95%CI) | 1.17  (1.11, 1.24)  Male:1.17  (1.08, 1.26) Female:1.17  (1.09, 1.27) |  |  |  |  |  |  |  |  |  |  |  |
| 34 | CVD;  CVD Risk | OR (95%CI) AUC (95%CI) | CVD;  OR 2.641  (1.241,5.617) AUC 0.656( 0.576, 0.737) CVD Risk OR 1.876  (0.805,4.373) AUC 0.475  (0.375,0.574) | CVD;  OR 0.742  (0.551, 0.999) AUC  0.640  ( 0.557, 0.723) CVD Risk OR 0.782  (0.561, 1.092) AUC  0.498  (0.398, 0.598) |  |  | WHR CVD;  OR 0.074  (0.001, 4.840) AUC  0.364  (0.282, 0.447) CVD Risk OR 0.009  (0.000, 1.227) AUC 0.361(0.278,0.44) | NC CVD;  OR 1.196  (1.033,1.383) AUC  0.605  ( 0.521,0.69) CVD Risk OR  1.320(1.090, 1.600) AUC 0.557  ( 0.454,0.66) |  |  |  |  |  |  |
| 35 | Type 2 Diabetes | OR (95%CI) AUC (95%CI) | OR Male:1.943(1.539, 2.455) Female:2.428  (1.929,3.056) AUC Male: 0.592  (0.558,0.627) Female: 0.641  (0.613,0.670) | OR Male:1.803  (1.422, 2.286) Female:2.450  (1.972, 3.043)  AUC Male: 0.572  (0.538,0.606) Female: 0.625  (0.596,0.655) | OR Male:1.995  (1.573, 2.530) Female:2.505  (1.993, 3.148)  AUC Male:0.583  (0.549,0.618) Female: 0.640  (0.612,0.669) | OR Male:1.921  (1.521,2.462) Female:2.409(1.914,3.032)  AUC Male: 0.592  ( 0.558,0.627) Female: 0.641  (0.613,0.670) | CVAI OR Male:1.991  (1.575,2.517) Female:2.717  (2.172, 3.399)  AUC Male: 0.593  (0.559,0.628) Female:0.663  ( 0.636,0.691) | ABSI OR Male:1.637  (1.277, 2.099) Female:1.415(1.117, 1.791)  AUC Male: 0.554  (0.521,0.587) Female: 0.559  ( 0.529,0.58) | VAI OR Male:1.838  (1.429, 2.363) Female:2.059  (1.656, 2.560)  AUC Male: 0.565  (0.531,0.599) Female: 0.608  (0.580,0.637) | LAP OR Male:1.972(1.557, 2.497) Female:2.662(2.110,3.358)  AUC Male: 0.583  ( 0.549,0.617) Female:0.643  (0.616,0.670) | CI OR Male:1.840  (1.455, 2.327) Female:1.881  (1.500,2.360)  AUC Male: 0.578  ( 0.544,0.612) Female: 0.601  ( 0.572,0.630) | TyG OR Male:1.804  (1.423, 2.287) Female:2.187(1.735, 2.757)  AUC Male: 0.576  (0.543,0.609) Female: 0.610  (0.582,0.639) | TyG-WHtR OR Male:2.249  (1.771, 2.857) Female:2.61  3(2.104, 3.244)  AUC Male: 0.600 (0.566,0.634) Female:0.664  ( 0.636,0.691) | TyG-BMI OR Male:2.071  (1.629, 2.633) Female:2.607  (2.096,3.242)  AUC Male: 0.586  ( 0.552,0.620 ) Female: 0.647  ( 0.619,0.675) |
| 36 | Type 2 Diabetes | OR (95%CI) AUC (95%CI) | OR 1.67  (1.31, 2.14)  AUC 0.72  (0.69, 0.75) | OR 1.67  (1.31, 2.15)  AUC  0.67  (0.64, 0.69) | OR 1.86 (1.60, 2.15)  AUC  0.71 (0.68, 0.74) | WHtR OR 1.84  (1.59, 2.13)  AUC 0.72  (0.69, 0.75) | ABSI OR 1.50  (1.27, 1.76)  AUC 0.61  (0.58, 0.64) | WHR OR 1.73  (1.48, 2.01)  AUC 0.69  (0.66, 0.72) | CI OR 1.45 (1.24, 1.71)  AUC  0.69  (0.66, 0.71) | AVI OR  1.72  (1.35, 2.20)  AUC 0.71  (0.68, 0.73) | BAI OR 0.83 (0.67, 1.04)  AUC 0.63 (0.60, 0.66) | CUN-BAE OR 3.72 (1.74, 7.97)   AUC 0.70 (0.68, 0.73) |  |  |
| 37 | Type 2 Diabetes | OR (95%CI) AUC (95%CI) | OR  1.70  (1.35, 2.14)  AUC Male: 0.648 (0.605, 0.689) Female: 0.685 (0.661, 0.709) | OR 1.33  (1.07, 1.64)  AUC Male:0.573 (0.530, 0.615) Female: 0.610 (0.585, 0.635) |  | OR 1.76  (1.38, 2.23)  AUC Male:0.648 (0.605, 0.689) Female:0.685 (0.661,0.709) | ABSI OR 1.50 (1.18, 1.91)  AUC Male: 0.599 (0.555, 0.641) Female: 0.669 (0.644, 0.692) | WHR OR 1.87  (1.43, 2.43)  AUC Male: 0.654 (0.612, 0.695) Female:0.687 (0.663,0.710) | BAI OR  1.36  (1.09, 1.68)  AUC Male: 0.590 (0.546, 0.632) Female:0.583 (0.557, 0.608) | VAI OR  1.27  (1.04, 1.55) AUC Male: 0.596 (0.552,0.640) Female: 0.658  (0.633,0.683) |  |  |  |  |
| 38 | Hyperuricemia | OR (95%CI) AUC (95%CI) | OR 1.26 (1.25,1.28) AUC 0.6692 (0.6617,0.677) | OR 1.08  (1.08, 1.09)  AUC 0.6635  (0.656,0.6711) |  |  | WWI OR 1.72  (1.64, 1.80) AUC 0.6222  (0.6144, 0.63) |  |  |  |  |  |  |  |
| 39 | HTN | OR (95%CI) | 1.276  (1.224, 1.330) | 1.063 (1.047,1.080) |  |  | VAI 1.029  (1.020, 1.038) | BAI 0.956  (0.945, 0.968) |  |  |  |  |  |  |
| 40 | Hyperuricemia | AUC (95%CI) | Male: 0.592 (0.558, 0.626)  Female:0.669 (0.638, 0.700) | Male: 0.586 (0.552, 0.619)  Female:0.644 (0.611, 0.678) | Male: 0.579 (0.545, 0.614)  Female:0.664 (0.632, 0.696) | Male: 0.592 (0.558,0.626)  Female:0.669 (0.638,0.700) | VAI Male: 0.596 (0.562, 0.630)  Female:0.664 (0.630, 0.697) | LAP Male: 0.612 (0.579, 0.645)  Female:0.691 (0.660, 0.723) | CI Male: 0.563 (0.529, 0.597)  Female:0.616 (0.583, 0.650) | CVAI Male: 0.593 (0.559,0.627)  Female:0.707 (0.676,0.737) |  |  |  |  |
| 41 | Hyperuricemia | OR (95%CI) AUC (95%CI) | OR Male:1.45 (1.24, 1.70) Female:1.58 (1.44, 1.73)  AUC Male:0.693 (0.658, 0.728) Female:0.683 (0.661, 0.704) | OR Male:1.42 (1.20, 1.67) Female:1.67 (1.52, 1.83)  AUC Male:0.683 (0.648, 0.719) Female:0.694 (0.673, 0.716) | OR Male:1.45  (1.23, 1.71) Female:1.61 (1.46, 1.78)  AUC Male:0.688 (0.653, 0.724) Female:0.684 (0.663, 0.706) |  | CMI OR Male:1.36  (1.17, 1.58) Female:1.41 (1.29, 1.55)  AUC Male:0.693 (0.658, 0.729) Female:0.678 (0.657, 0.700) | VAI OR Male:1.33 (1.14, 1.54) Female:1.34 (1.23, 1.47)  AUC Male:0.691 (0.655, 0.727) Female:0.672 (0.650, 0.693) | LAP OR Male:1.41 (1.20, 1.64) Female:1.55 (1.41, 1.70)  AUC Male:0.697 (0.662, 0.732 ) Female:0.689 (0.668, 0.710) | AVI OR Male: 1.43 (1.22, 1.67) Female:1.57 (1.43, 1.73)  AUC Male:0.688 (0.652, 0.723) Female:0.684 (0.663, 0.706) | OR Male:1.53  (1.30, 1.81) Female:2.05 (1.84, 2.29)  AUC Male:0.697 (0.662, 0.732) Female:0.713 (0.693, 0.733) |  |  |  |
| 42 | Diabetes Mellitus | HR 95% CI; AUC (95%CI) | HR 1.45 (1.25,1.68) AUC  0.675 (0.638,0.713) Male  0.645 (0.599,0.691) Female  0.693 (0.619,0.768) |  | HR 1.42 (1.19, 1.68) AUC  0.676  (0.639, 0.714) Male  0.611  (0.561, 0.660) Female  0.672  (0.599, 0.745) | HR 1.47  (1.25, 1.72) AUC  0.676 (0.639,0.713) Male  0.647 (0.601,0.692) Female  0.692 (0.618,0.766) | HR 1.53 (1.27, 1.85) AUC  0.733  (0.700, 0.767) Male 0.670  (0.625, 0.715) Female 0.759  (0.639, 0.826) | LAP HR 1.27  (1.18, 1.38) AUC  0.709 (0.670,0.747) Male 0.656  (0.607,0.705) Female 0.750  (0.677,0.824) | VAI HR 1.24 (1.16, 1.32) AUC  0.708  (0.669, 0.748) Male  0.666  (0.617, 0.716) Female 0.752  (0.681, 0.822) |  |  |  |  |  |
| 43 | HTN;  Hyperuricemia | AUC (95%CI) | HTN;  0.63  (0.61, 0.63)  Hyperuricemia 0.58  (0.57, 0.59) |  |  |  | LAP HTN;  0.59 (0.58, 0.60)  Hyperuricemia;  0.62 (0.61, 0.63) | VAI HTN;  0.54  (0.53, 0.55)  Hyperuricemia  0.60  (0.59, 0.61) | TyG HTN;  0.58 (0.57, 0.59)  Hyperuricemia;  0.58 (0.57, 0.60) | ABSI HTN;  0.64 (0.63, 0.64)  Hyperuricemia;  0.52  (0.51, 0.54) | CMI HTN;  0.55 (0.54, 0.56)  Hyperuricemia;  0.63 (0.62, 0.64) | AIP HTN;  0.53 (0.52, 0.54)  Hyperuricemia;  0.62 (0.61, 0.63) |  |  |
| 44 | MetS | OR (95%CI) | 2.145  (1.706, 2.696) | 1.208  (1.129, 1.292) |  |  | ABSI 1.145  (1.077, 1.217) | LAP 1.050  (1.034, 1.067) |  |  |  |  |  |  |
| 45 | CVD | HR (95%CI) | Total women 1.16 (1.06,1.27)  Menopausal women 1.12 (1.05,1.29)  Non-menopausal women 1.36 (1.00,1.86) | Total women 1.05 (1.02,1.08)  Menopausal women 1.05 (1.01,1.09)  Non-menopausal women 1.08 (0.97,1.19) | Total women 1.02 (1.01,1.03)  Menopausal women 1.02 (1.00,1.04)  Non-menopausal women 1.05 (1.01,1.11) |  | CI Total women 1.39 (1.04,1.85)  Menopausal women 1.25 (1.08,1.92)  Non-menopausal women 2.32 (1.10,4.36) |  |  |  |  |  |  |  |
| 46 | HTN; Dyslipidaemia; Type 2 Diabetes | OR (95%CI) AUC (95%CI) | HTN OR 1.427  (1.350, 1.509) AUC  0.635  (0.623, 0.646) Dyslipidaemia OR 1.645 (1.567, 1.728) AUC  0.641  (0.630, 0.652) Type 2 Diabetes OR 1.171  (1.105, 1.242) AUC  0.613  (0.597, 0.628) | HTN OR  1.400  (1.328, 1.475) AUC  0.619  (0.608, 0.631) Dyslipidaemia OR  1.602  (1.529, 1.679) AUC 0.655  (0.645, 0.666) Type 2 Diabetes OR 1.164  (1.097, 1.235) AUC  0.601  (0.585, 0.617) | HTN OR  1.413  (1.341, 1.489) AUC  0.615  (0.604, 0.627) Dyslipidaemia OR 1.660  (1.582, 1.741) AUC 0.650  (0.640, 0.661) Type 2 Diabetes OR 1.193  (1.121, 1.269) AUC  0.617  (0.601, 0.632) | HTN OR 1.402  (1.332, .476) AUC  0.635 (0.623,0.646) Dyslipidaemia OR  1.661 (1.585,1.740) AUC 0.641  (0.630, 0.652) Type 2 Diabetes OR 1.215  (1.143,1.291) AUC  0.613 (0.597,0.628) | BF HTN OR 1.735  (1.593, 1.890) AUC 0.605  (0.594, 0.617) Dyslipidaemia OR 2.165  (2.005, 2.338) AUC 0.542  (0.530, 0.553) Type 2 Diabetes OR 1.282  (1.163, 1.414) AUC 0.536  (0.519, 0.553) | CI HTN OR  1.175  (1.116, 1.238) AUC  0.586  (0.574, 0.597) Dyslipidaemia OR  1.308  (1.249,1.370) AUC 0.582  (0.571,0.593) Type 2 Diabetes OR  1.133  (1.067, 1.202) AUC  0.582  (0.566, 0.598) | ABSI HTN OR 1.038  (0.988 1.092) AUC  0.548  (0.537, 0.560) Dyslipidaemia OR  1.105  (1.059, 1.154) AUC  0.531 (0.519, 0.542) Type 2 Diabetes OR  1.084 (1.022, 1.150) AUC  0.547  (0.531, 0.563) | BAI HTN OR 1.171  (1.110, 1.236) AUC  0.547  (0.535, 0.558) Dyslipidaemia OR 1.252  (1.195, 1.312) AUC 0.533  (0.522, 0.545) Type 2 Diabetes OR 0.998 (0.931, 1.070) AUC 0.491  (0.474, 0.508) | WHR HTN OR 1.378  (1.297, 1.465) AUC  0.617  (0.606, 0.629) Dyslipidaemia OR 1.534 (1.455, 1.616) AUC 0.622  (0.611, 0.632) Type 2 Diabetes OR  1.224  (1.146, 1.308) AUC 0.639  (0.623, 0.654) | AVI HTN OR  1.458  (1.37, 1.546) AUC 0.614  (0.602, 0.625) Dyslipidaemia OR 1.649  (1.568, 1.736) AUC  0.650 (0.639, 0.660) Type 2 Diabetes OR  1.131  (1.068, 1.197) AUC 0.614  (0.599, 0.630) | LAP HTN OR 1.407  (1.319, 1.500) AUC  0.624  (0.613, 0.636) Dyslipidaemia OR 23.358  (20.498, 26.618)  Type 2 Diabetes OR 1.388  (1.320, 1.460) AUC  0.643  (0.627, 0.659) | VAI HTN OR  1.166  (1.101, 1.236) AUC  0.586  (0.574, 0.597)  Type 2 Diabetes OR 1.236  (1.176, 1.298) AUC  0.623  (0.607, 0.640) |
| 47 | HTN | AUC (95%CI) | 0.665  (0.660, 0.671) Male 0.637  (0.629, 0.645)  Female 0.682  (0.670, 0.691) | 0.652  (0.646, 0.658) Male  0.625 (0.617, 0.632)  Female 0.659  (0.650, 0.668) | 0.663  (0.658, 0.669) Male 0.620  (0.612, 0.628)  Female 0.668  (0.659, 0.677) |  | WHtR 0.665  (0.660, 0.671) Male 0.637  (0.629, 0.645)  Female 0.691  (0.682, 0.700) | ABSI 0.577  (0.571, 0.583) Male 0.546  (0.537,0.554)  Female 0.589  (0.579, 0.598) |  |  |  |  |  |  |
| 48 | MAFLD Diabetes Mellitus HTN  Dyslipidemia | OR (95%CI) | Male MAFLD  1.491 (1.332, 1.668) Diabetes  1.61  (1.34, 1.93) HTN 1.21 (1.10, 1.33) Dyslipidemia  1.30 (1.21, 1.40) Female MAFLD 1.377  (1.294, 1.466) Diabetes   1.32  (1.20, 1.45)  HTN  1.38  (1.29, 1.47) Dyslipidemia  1.18  (1.13, 1.23) | Male MAFLD 1.184 (1.135, 1.235) Diabetes   1.14  (1.06, 1.23) HTN 1.10 (1.06, 1.14)  Dyslipidemia 1.09  (1.06, 1.12) Female MAFLD 1.126 (1.099, 1.154)  Diabetes  1.08  (1.04, 1.12) HTN  1.13  (1.10, 1.15)  Dyslipidemia  1.05  (1.03, 1.07) | Male MAFLD 1.067 (1.050, 1.085) Diabetes  1.08 (1.05, 1.11) HTN  1.03 (1.01, 1.04)   Dyslipidemia 1.04 (1.03, 1.05) Female MAFLD  1.060 (1.048, 1.072) Diabetes   1.06 (1.04, 1.08)  HTN 1.060 (1.05, 1.07) Dyslipidemia 1.03 (1.02, 1.04) | WHtR Male MAFLD 0.021  (0.008, 0.057)  Diabetes   0.007 (0.01, 0.05) HTN  0.17  (0.08, 0.35)  Dyslipidemia  0.12 (0.08, 0.19) Female MAFLD 0.018  (0.008, 0.038) Diabetes  0.02 (0.01, 0.08) HTN  0.02  (0.01, 0.04) Dyslipidemia 0.15  (0.10, 0.23) | WHR Male MAFLD 1.114  (1.081, 1.147) Diabetes  1.20  (1.14, 1.27) HTN 1.04 (1.02, 1.06)  Dyslipidemia 1.07 (1.06, 1.09) Female MAFLD  1.083  (1.065, 1.101) Diabetes   1.12  (1.09, 1.15) HTN  1.02  (1.01, 1.04) Dyslipidemia 1.06  (1.05, 1.07) | BAI Male MAFLD 1.117  (1.082,1.153) Diabetes  1.15  (1.09, 1.21) HTN  1.05  (1.03, 1.09)  Dyslipidemia  1.07 (1.05, 1.10) Female MAFLD  1.099  (1.079, 1.121) Diabetes  1.08 (1.05, 1.11) HTN 1.10 (1.08, 1.12) Dyslipidemia 1.05  (1.04, 1.06) | VAI Male MAFLD 1.065  (1.020, 1.111) Diabetes   1.13  (1.07, 1.19) HTN  1.02  (0.97, 1.07)   Dyslipidemia  14.35  (11.57, 17.79) Female MAFLD  1.150  (1.105, 1.196) Diabetes   1.20  (1.14, 1.26) HTN 1.15 (1.10, 1.17)  Dyslipidemia  7.41  (6.44, 8.52) | WWI Male MAFLD 1.818  (1.399, 2.364) Diabetes  3.81  (2.40, 6.04) HTN  1.27  (1.03, 1.56)  Dyslipidemia  1.61  (1.40, 1.86) Female MAFLD 1.760 (1.486, 2.083) Diabetes   2.28 (1.73, 3.01) HTN  1.76 (1.49, 2.08) Dyslipidemia  1.51  (1.35, 1.68) | CI Male MAFLD 1.057  (1.033, 1.082) Diabetes   1.13 (1.08, 1.18) HTN 1.02 (1.00, 1.04)  Dyslipidemia  1.04 (1.03, 1.05) Female MAFLD 1.052 (1.036, 1.068) Diabetes   1.09 (1.06, 1.11) HTN 1.01 (1.00, 1.03) Dyslipidemia 1.04 (1.0, 1.05) | AVI Male MAFLD 1.153  (1.110, 1.198) Diabetes   1.19 (1.11, 1.26) HTN  1.06 (1.03, 1.10)  Dyslipidemia 1.09 (1.07, 1.12) Female MAFLD 1.140  (1.112, 1.169)  Diabetes  1.13 (1.09, 1.18) HTN 1.14 (1.11, 1.17) Dyslipidemia 1.07 (1.05, 1.09) |  |  |
| 49 | Type 2 Diabetes | HR (95%CI) AUC (95%CI) | HR 2 (1.23, 3.24)  AUC 0.615 (0.536, 0.694) | HR 1.65  (1.048, 2.602)  AUC 0.615  (0.537, 0.693) | HR 1.75 (1.09, 2.81)  AUC 0.623  (0.545, 0.701) |  | AVI HR 1.63  (1.03, 2.57)  AUC 0.623  (0.545, 0.701) | VAI HR 2.10  (1.31, 3.36)  AUC 0.616  (0.539, 0.693) | LAP HR 2.18 (1.36, 3.50)  AUC 0.627  (0.550, 0.704) | WHR HR 0.93  (0.59, 1.45) | ABSI HR 0.83 (0.53, 1.30) |  |  |  |
| 50 | Hyperuricemia | AUC (95%CI) | 0.6204 (0.6145,0.626)  Male 0.6027 (0.5956,0.610)  Female 0.6812 (0.6666,0.696) | 0.655  (0.6491,0.6609)  Male 0.6186 (0.6115, 0.6257)  Female 0.675  (0.6601,0.6898) | 0.6892 (0.6835, 0.6948)  Male 0.6164 (0.6093, 0.6235)  Female 0.6813  (0.6666, 0.6959) | 0.6204 (0.6145, 0.6264)  Male 0.6027  (0.5956,0.610)  Female 0.6812  (0.6666,0.696) | ABSI 0.5606 (0.5547, 0.5666)  Male 0.5189 (0.5118, 0.5261)  Female 0.5788  (0.5632,0.5944) | LAP 0.6835 (0.6775, 0.6895)  Male 0.6571 (0.6501,0.664)  Female 0.7326 (0.7188,0.746) | VAI 0.6573  (0.6513, 0.6633)  Male 0.6517  (0.6448, 0.6585)  Female 0.7193  (0.7054, 0.7331) | TyG 0.6774 (0.6716,0.683)  Male 0.6326 (0.6256,0.640)  Female 0.7143 (0.7002,0.728) | WTI:waist circumference-triglyceride index  0.7015  (0.6958, 0.7073)  Male 0.652  (0.6451, 0.659) Female 0.7259  (0.7121, 0.7397) | WWI 0.5417  (0.5357, 0.5477)  Male 0.5461  (0.539, 0.5533)  Female 0.6258  (0.6105, 0.6411) |  |  |
| 51 | All-cause and  Cardiovascular Mortality | HR (95%CI) | All-cause Mortality 1.01  (0.99, 1.04)  Cardiovascular Mortality 1.05  (1.01, 1.10) |  |  |  |  |  |  |  |  |  |  |  |
| 52 | CKD | OR (95%CI) AUC (95%CI) | OR 1.16  (1.10, 1.23) AUC 0.625  (0.616, 0.633) | AUC 0.553  (0.544, 0.562) | AUC 0.599  (0.590, 0.607) |  | ABSI AUC 0.619  (0.610, 0.627) |  |  |  |  |  |  |  |
| 53 | CKD | AUC (95%CI) | 0.595  (0.550, 0.640) |  | 0.571  (0.525, 0.617) | WHtR 0.595  (0.550, 0.640) | ABSI 0.596  (0.552, 0.639) | VAI 0.569  (0.525, 0.612) | LAP 0.572  (0.528, 0.616) |  |  |  |  |  |
| 54 | Diabetes Mellitus; Heart disease; Stroke; CVD | HR (95%CI) | Diabetes:1.20 (1.12,1.28) Male:1.17 (1.04,1.31) Female:1.20 (1.11,1.31) Heart disease:  1.02 (0.98,1.07) Male:1.0 (0.92,1.09) Female:1.03 (0.97,1.09) Stroke:1.09 (1.01,1.19) Male:1.06 (0.93,1.20) Female:1.11 (1.0,1.24)  CVD:1.08 (1.04,1.12) Male:1.04 (0.97,1.11) Female:1.09 (1.04,1.14) |  |  |  |  |  |  |  |  |  |  |  |
| 55 | Hyperuricemia | OR (95%CI) AUC (95%CI) | OR: 1.272 (1.261, 1.284) Male 1.241  (1.224, 1.258)  Female 1.296  (1.281, 1.312) AUC: 0.702 (0.699, 0.706) Male 0.640  (0.634, 0.645) Female 0.720  (0.715, 0.725) | OR: 1.143 (1.138, 1.148) Male 1.121  (1.113, 1.129) Female 1.163  (1.155, 1.170) AUC: 0.716 (0.713, 0.720) Male 0.655  (0.649, 0.660) Female 0.728  (0.723, 0.733) |  | WHtR OR: 1.085 (1.081, 1.088) Male 1.077  (1.072, 1.082) Female 1.090  (1.085, 1.094) AUC: 0.680 (0.677, 0.684) Male 0.633 (0.628, 0.639) Female 0.721 (0.716, 0.726) | WHR OR: 1.057 (1.054, 1.060) Male 1.058  (1.053, 1.063) Female 1.054 (1.050, 1.058) AUC: 0.680 (0.676, 0.683) Male 0.599  (0.594, 0.605) Female 0.676  (0.671, 0.982) | CI OR: 1.389 (1.358, 1.420) Male 1.460  (1.406, 1.516) Female 1.340  (1.303, 1.378) AUC: 0.612 (0.608, 0.615) Male 0.574  (0.568, 0.579) Female 0.626  (0.620, 0.631) | BAI  OR: 1.106 (1.101, 1.111) Male 1.095  (1.087, 1.103) Female 1.113  (1.107, 1.120) AUC: 0.543 (0.539, 0.547) Male 0.605 (0.600, 0.611) Female 0.673 (0.668, 0.679) | AVI OR: 1.140 (1.135, 1.146) Male 1.119  (1.111, 1.128) Female 1.162  (1.154, 1.170) AUC: 0.712 (0.709, 0.716) Male 0.642 (0.637, 0.648) Female 0.713  (0.708, 0.718) | ABSI OR: 1.156 (1.115, 1.198) Male 1.260  (1.183, 1.341) Female 1.088  (1.040, 1.138) AUC: 0.529 (0.525, 0.533) Male 0.510 (0.504, 0.516) Female 0.544  (0.538, 0.550) | LAP OR: 1.013  (1.012, 1.014) Male 1.010  (1.009, 1.011) Female 1.018  (1.017, 1.019) AUC: 0.725 (0.722, 0.729) Male 0.669 (0.664, 0.675) Female 0.754  (0.750, 0.759) | VAI OR: 1.306  (1.293, 1.3190) Male 1.238  (1.219, 1.258) Female 1.339  (1.321, 1.357)  AUC: 0.686 (0.682, 0.690) Male 0.645  (0.640, 0.651) Female 0.724  (0.719, 0.729) |  |
| 56 | All-cause Mortality | HR (95%CI) | 0.49  (0.34, 0.70) | 0.70  (0.61, 0.81) |  |  |  |  |  |  |  |  |  |  |
| 57 | MetS | AUC (95%CI) | Male  0.94  (0.92, 0.95)  Female 0.85  (0.83, 0.86) |  |  |  | ABSI Male 0.60  (0.57, 0.64)  Female 0.50  (0.47, 0.53) | CI Male  0.76  (0.73, 0.79)  Female 0.63  (0.60, 0.66) | NC Male  0.78  (0.75, 0.81)   Female 0.72 (0.69, 0.74) |  |  |  |  |  |
| 58 | Hyperuricemia | AUC (95%CI) | 0.624  (0.585, 0.664) | 0.600  (0.560, 0.640) |  | 0.624  (0.582, 0.664) | ABSI  0.579  (0.539, 0.619) | WHR 0.582  (0.542, 0.622) | AVI 0.621  (0.582, 0.660) | BAI 0.555  (0.513, 0.598) | CI 0.61  1 (0.571, 0.650) | WWI 0.503  (0.459, 0.547) | CUN-BAE 0.570  (0.529, 0.612) |  |
| 59 | HTN; Diabetes Mellitus; CHD; Stroke | OR (95%CI) AUC (95%CI) | HTN OR 1.59  (1.56, 1.61)  Diabetes Mellitus OR 1.44  (1.42, 1.47)  CHD OR 1.14  (1.09, 1.19)  Stroke OR 1.12  (1.08, 1.17) | HTN OR 1.64  (1.62, 1.67)  Diabetes Mellitus OR 1.39 (1.37, 1.42)  CHD OR 1.12 (1.07, 1.17)  Stroke OR 1.07  (1.02, 1.12) | HTN OR 1.63  (1.61, 1.66)  Diabetes Mellitus OR 1.50  (1.48, 1.53)  CHD OR 1.16  (1.11, 1.22)  Stroke OR 1.13  (1.08, 1.18) | HTN OR 1.60  (1.58, 1.63)  Diabetes Mellitus OR 1.47  (1.44, 1.50)  CHD OR 1.15 (1.10, 1.21)  Stroke OR 1.13  (1.08, 1.18) | ABSI HTN OR 1.07  (1.06, 1.09)  Diabetes Mellitus OR 1.18  (1.15, 1.20)  CHD OR 1.08  (1.03, 1.14)  Stroke OR 1.11  (1.06, 1.16) | CI HTN OR 1.27  (1.25, 1.29)  Diabetes Mellitus OR 1.33  (1.30, 1.36)  CHD OR 1.12  (1.07, 1.18)  Stroke OR 1.14  (1.09, 1.19) |  |  |  |  |  |  |
| 60 | Diabetes Mellitus | AUC (95%CI) | Male 0.7543  (0.698, 0.8106)  Female 0.7118 (0.6801,0.744) | Male 0.7539  (0.6999,0.8080)  Female 0.6802  (0.6469,0.7136) | Male 0.7287  (0.6714, 0.786)  Female 0.6961  (0.6624, 0.7298) | Male 0.7543  (0.698, 0.8106)  Female 0.7118 (0.6801, 0.7435) | ABSI Male 0.5821  (0.5196,0.6447) Female 0.5943  (0.5608,0.6277) | VAI Male 0.783  (0.733,0.8328) Female 0.6962 (0.6634,0.729) | LAP Male 0.7922  (0.7396, 0.8447) Female 0.713  (0.6806, 0.7454) |  |  |  |  |  |
| 61 | HTN | AUC (95%CI) | AUC Male 0.593 (0.563,0.623) Female 0.609 (0.579,0.638) | AUC Male 0.565 (0.534,0.597) Female 0.575 (0.545,0.606) | AUC Male  0.583 (0.552,0.613) Female  0.598 (0.569,0.628) | AUC Male  0.593 (0.563,0.623) Female  0.609 (0.579,0.638) | VAI AUC Male 0.548 ( 0.517,0.579 ) Female  0.559 (0.529,0.589) | ABSI AUC Male 0.569 (0.540,0.599) Female  0.563 (0.533,0.594) | LAP AUC Male  0.579 (0.548,0.61) Female  0.594 (0.565,0.624) | CI AUC Male  0.586 (0.556,0.616) Female 0.589 (0.559,0.619) | TyG AUC Male  0.572 (0.541,0.602) Female  0.568 (0.538,0.598) | TyG‑BMI AUC Male  0.582 (0.551,0.613 ) Female  0.589 (0.559,0.619) | TyG‑WC AUC Male  0.592 (0.561,0.622) Female  0.608 (0.578,0.637) | CVAI AUC Male 0.590 (0.559,0.621) Female 0.633 (0.604,0.662 ) |
| 62 | MAFLD | AUC (95%CI) | Total 0.78  (0.774, 0.788) Male 0.73  (0.721, 0.744) Female 0.83  (0.827, 0.845) | Total 0.89  (0.883, 0.894) Male 0.84  (0.835, 0.853) Female 0.89  (0.881, 0.897) | Total 0.84  (0.832, 0.845) Male 0.77  (0.758, 0.780) Female 0.84  (0.835, 0.853) | Total 0.85  (0.839, 0.852) Male 0.78 (0.765, 0.786) Female 0.86  (0.847, 0.864) | ABSI Total 0.50  (0.495, 0.513) Male 0.59  (0.578, 0.603) Female 0.50  (0.492, 0.516) | LAP Total 0.87  (0.863, 0.874)  Male 0.81  (0.798, 0.818) Female 0.88  (0.874, 0.890) | VAI Total 0.79  (0.782, 0.796) Male 0.75  (0.740, 0.762) Female 0.82  (0.807, 0.826) |  |  |  |  |  |
| 63 | Type 2 Diabetes | OR (95%CI) AUC (95%CI) | OR Male 1.263 (1.193, 1.337) Female 1.171 (1.123, 1.221)  AUC Male 0.677 (0.653,0.701) Female 0.708 (0.689,0.727) | OR Male 1.140 (1.105, 1.175) Female 1.099  (1.073, 1.125)  AUC Male 0.660 (0.635,0.685) Female 0.700 (0.680,0.720) |  | OR Male 1.088 (1.066,1.110) Female 1.056 (1.041,1.070)  AUC Male 0.684 (0.661,0.708) Female 0.710 (0.691,0.729) | WHR OR Male 1.082 (1.061,1.104) Female 1.051 (1.037,1.065)  AUC Male 0.683 (0.659,0.707) Female 0.693 (0.673,0.713) | CI  OR Male 1.425 (1.208,1.682) Female 1.233 (1.118,1.359)  AUC Male 0.630 (0.605,0.655) Female 0.630 (0.609,0.651) | BAI OR Male 1.082 (1.048,1.117) Female 1.043 (1.022,1.066)  AUC Male 0.626 (0.600,0.652) Female 0.642 (0.620,0.663) | AVI  OR Male 1.124 (1.089,1.159) Female 1.095 (1.068,1.123)  AUC Male 0.661 (0.637,0.685) Female 0.698 (0.679,0.717) | LAP OR Male 8.951 (5.454,14.689) Female 8.687 (5.782,13.052)   AUC Male 0.692 (0.668,0.716) Female 0.744 (0.725,0.762) | VAI OR Male 9.104 (6.141,13.497) Female 6.629 (4.864,9.035)  AUC Male 0.671 (0.647,0.696) Female 0.710 (0.690,0.729) |  |  |
| 64 | CVD; CHD; Stroke | HR (95%CI) | CVD 1.27  (1.12, 1.43) Male 1.27(1.11,1.45) Female 1.30  (1.08, 1.56) CHD 1.22  (1.04, 1.43) Male 1.23  (1.04, 1.45) Female 1.18  (0.92, 1.52) Stroke 1.35  (1.11, 1.65) Male 1.35  (1.07, 1.69) Female 1.44  (1.11, 1.89) |  |  |  |  |  |  |  |  |  |  |  |
| 65 | HTN | AUC (95%CI) | Male 0.724 (0.623,0.824)   Female 0.703 (0.628,0.777) | Male 0.672 (0.567,0.777)   Female 0.667 (0.588,0.744) |  | Male 0.724 (0.623,0.824)   Female 0.703 (0.628,0.777) | WHR Male 0.645 (0.540,0.750)   Female 0.668 (0.587,0.749) | ABSI Male 0.610 (0.483,0.738)  Female 0.605 (0.517,0.693) | AVI Male 0.688 (0.585,0.791)   Female 0.728 (0.658,0.799) | CI Male 0.657 (0.559,0.789)   Female 0.674 (0.593,0.755) |  |  |  |  |
| 66 | MetS | AUC (95%CI) | 0.844  (0.794, 0.895) | 0.809  (0.755, 0.864) | 0.849  (0.800, 0.899) |  | VAI 0.836  (0.773, 0.901) | ABSI 0.606  (0.532, 0.681) | LAP 0.915  (0.878, 0.953) |  |  |  |  |  |
| 67 | CVD | OR (95%CI) | Male 1.54 (1.45,1.64)  Female 1.22 (1.16,1.28) | Male 1.61 (1.51,1.72)  Female 1.26 (1.21,1.33) | Male 1.62 (1.52,1.74)  Female 1.27 (1.21,1.3) | Male 1.61 (1.51,1.72)  Female 1.25 (1.19,1.31) | WHR Male 1.56 (1.46,1.67)  Female 1.24 (1.18,1.29) | AVI Male 1.55 (1.46,1.64) Female 1.24 (1.18,1.29) | CI Male 1.36 (1.27,1.45)  Female 1.14 (1.09,1.2) | ABSI Male 1.14 (1.07,1.22) Female 1.06 (1.00,1.11) | BAI Male 1.28 (1.20,1.36)  Female 1.10 (1.05,1.16) |  |  |  |
| 68 | HTN | OR (95%CI) AUC (95%CI) | OR 1.7 (1.66, 1.74)  AUC 0.696  (0.691,0.701) | OR 1.68  (1.65, 1.71)  AUC 0.656  (0.651,0.661) | OR 1.71 (1.68, 1.75)  AUC 0.684(0.678,0.69) | OR 1.68  (1.65, 1.72)  AUC 0.651  (0.646,0.656) | CI OR 1.51  (1.48, 1.54)  AUC 0.71  (0.706,0.715) | ABSI OR 3.4  (2.73, 4.24)  AUC 0.66  (0.655,0.665) | LAP OR 1.31 (1.29, 1.34)  AUC 0.63 (0.625,0.635) |  |  |  |  |  |
| 69 | Type 2 Diabetes | HR (95%CI) AUC (95%CI) | HR 1.570  (1.360, 1.811) Male 1.480  (1.254, 1.747) Female 1.845  (1.461, 2.330) AUC Male 0.7061 (0.6740,0.738) Female 0.7354 (0.6755,0.795) | AUC Male 0.6732  (0.6395,0.7069) Female 0.7347  (0.6774,0.7920) | AUC Male 0.6898  (0.6556, 0.7240) Female 0.7069  (0.6465, 0.7673) |  | ABSI AUC Male 0.6371 (0.6017, 0.6724) Female 0.6941 (0.6381, 0.7501) |  |  |  |  |  |  |  |
| 70 | MetS | OR (95%CI) AUC (95%CI) | OR 1.819 (1.442,2.294)  AUC 0.832 (0.775,0.889) | OR 1.269  (1.165, 1.382)  AUC 0.838  (0.782, 0.895) |  | OR 0.030 (0.004,0.232)  AUC 0.832 (0.775,0.889) | BF OR 1.220  (1.136, 1.309)  AUC 0.848 (0.794, 0.902) | ABSI OR 1.754 (0.123,25.036)  AUC  0.677 (0.599,0.756) | CUN-BAE OR 1.210 (1.134,1.292)  AUC 0.846 (0.791,0.901) | CI OR 1.754 (0.123, 25.036)  AUC 0.762 (0.694, 0.831) |  |  |  |  |
| 71 | HTN | OR (95%CI) | 1.40 (1.08,1.81) | 1.58 (1.23,2.04) | 1.03 (1.00,1.05) | 1.39 (1.07,1.82) | BAI 1.41  (1.03, 1.92) |  |  |  |  |  |  |  |
| 72 | MAFLD | OR (95%CI) AUC (95%CI) | OR 2.22  (2.00, 2.47) Male 5.03  (4.02, 6.30) Female 2.74  (2.38, 3.16)  AUC 0.799  (0.786, 0.812) Male 0.825 (0.808, 0.843)  Female 0.802  (0.784, 0.821) | OR 3.44  (3.03, 3.90) AUC 0.791  (0.778, 0.805) Male 0.806  (0.787, 0.824) Female 0.790  (0.771, 0.809) |  | OR 3.64  (3.20, 4.14) Male 5.06  (4.07, 6.29)  Female 3.03  (2.61, 3.52)  AUC  0.799  (0.786, 0.812)  Male 0.825  (0.808, 0.843) Female 0.802  (0.784, 0.821) | LAP OR 3.79  (3.25, 4.40) Male 3.89  (3.14, 4.83)  Female 3.68  (3.00, 4.52)  AUC  0.813  (0.800, 0.826) Male 0.818  (0.800, 0.835) Female 0.808  (0.79, 0.826) | AVI OR 3.54  (3.12, 4.03)  Male 4.36  (3.55, 5.34) Female 3.10  (2.65, 3.62)  AUC 0.810  (0.797, 0.822) Male 0.821  (0.804, 0.839) Female 0.797  (0.778, 0.815) | HSI OR 4.12 (3.61, 4.71) Male 5.37 (4.33, 6.67) Female 3.50 (2.98, 4.11)  AUC 0.808 (0.796, 0.821) Male 0.823  (0.806, 0.841)  Female 0.809 (0.791, 0.827) | WHR OR 2.77  (2.44, 3.14) AUC 0.750  (0.736, 0.765)  Male 0.779  (0.759, 0.798) Female 0.719  (0.698, 0.741) | CMI OR 2.83 (2.43, 3.30) AUC 0.770 (0.756, 0.784) Male 0.764 (0.743, 0.784) Female 0.766  (0.745, 0.786) | TyG OR 2.22 (2.00, 2.47) AUC 0.742  (0.727, 0.756) Male 0.739  (0.717, 0.760) Female 0.733  (0.712, 0.755) | CI OR  2.58 (2.29, 2.91) AUC 0.752  (0.737, 0.766)  Male  0.772  (0.752, 0.792)  Female 0.73  (0.709, 0.752) | BAI OR 2.65 (2.35, 3.00)  AUC  0.678  (0.662, 0.694)  Male  0.773  (0.753, 0.793) Female 0.758  (0.738, 0.779) |
| 73 | HTN | OR (95%CI) AUC (95%CI) | OR Male 1.92 (1.57,2.35)  Female 1.70 (1.35,2.15) AUC Male 0.72 (0.68, 0.75) Female 0.78 (0.73,0.82) | OR Male 2.06 (1.67,2.53)  Female 1.90  (1.48, 2.45) AUC Male 0.68 (0.64,0.71) Female 0.75 (0.71,0.80) | OR Male 1.95 (1.60, 2.39)  Female 1.93 (1.47, 2.51) AUC Male 0.71 (0.67, 0.74) Female 0.77 (0.73, 0.82) | OR Male 1.98  (1.62, 2.43) Female 1.90 (1.45,2.49) AUC Male 0.70  (0.66, 0.73) Female 0.76  (0.71, 0.80) | BF OR Male 1.92  (1.50, 2.45)  Female 1.75  (1.25, 2.45) AUC Male 0.68  (0.65, 0.72) Female 0.72  (0.67, 0.77) | HC OR Male 1.85  (1.53, 2.24) Female 1.91  (1.48, 2.47) AUC Male 0.67  (0.63, 0.71) Female 0.72 (0.68,0.77) | WHR OR Male 2.06 (1.63, 2.62)  Female 1.85 (1.38, 2.52) AUC Male 0.73 (0.70, 0.76) Female 0.79 (0.75, 0.83) | ABSI OR Male 1.51 (1.21,1.88)  Female 1.39  (1.00, 1.94) AUC Male 0.70  (0.67, 0.74) Female 0.75 (0.70,0.80) | AVI OR Male 1.84 (1.54, 2.21)  Female 1.85 (1.46, 2.34) AUC Male 0.71 (0.67, 0.74) Female 0.77 (0.73, 0.81) | CI OR Male 1.79 (1.45, 2.23)  Female 1.70 (1.26, 2.31) AUC Male 0.72 (0.69, 0.76) Female 0.78 (0.73, 0.82) |  |  |
| 74 | HTN | OR (95%CI) AUC (95%CI) | OR Male 1.190 (1.141,1.241) Female 1.166 (1.131,1.201) AUC Male  0.621 (0.605,0.637) Female 0.652 (0.639,0.666) | OR Male 1.107 (1.083,1.131) Female 1.119 (1.101,1.137) AUC Male  0.597 (0.581,0.614) Female 0.645 (0.632,0.658) |  | OR Male 1.062 (1.048,1.077) Female 1.053 (1.043,1.062) AUC Male 0.632 (0.616,0.648) Female 0.662 (0.649,0.674) | WHR OR Male 1.038 (1.024,1.051) Female 1.027 (1.018,1.035) AUC Male 0.618 (0.602,0.634) Female  0.637 (0.624,0.651) | CI  OR Male 1.237 (1.112,1.375)  Female  1.095 (1.028,1.166) AUC Male  0.600 (0.584,0.617) Female 0.599 (0.585,0.613) | BAI  OR Male 1.084 (1.061,1.107) Female 1.078 (1.062,1.093) AUC Male 0.605 (0.588,0.621) Female 0.623 (0.610,0.637) | AVI OR Male 1.092 (1.068,1.117) Female 1.090 (1.071,1.110) AUC Male 0.604 (0.588,0.620)  Female  0.638 (0.624,0.651) | LAP OR Male 1.008 (1.005,1.111) Female 1.010 (1.007,1.013) AUC Male 0.610 (0.594,0.626) Female 0.657 (0.644,0.670) | VAI OR Male 1.084 (1.041,1.128) Female 1.095 (1.062,1.128) AUC Male 0.582 (0.566,0.599)  Female 0.624 (0.610,0.637) |  |  |
| 75 | Hyperuricemia | AUC (95%CI) | Male 0.513 (0.484,0.541)   Female 0.591 (0.539,0.644) | Male 0.515 (0.487,0.544)  Female 0.55 (0.495,0.604) | Male 0.507 (0.478,0.536)   Female  0.59 (0.538,0.642) | Male 0.513 (0.484,0.541)   Female 0.591 (0.539,0.644) | ABSI Male 0.514 (0.485,0.543)   Female 0.579 (0.525,0.632) | TyG Male 0.586 (0.557,0.614)  Female 0.728 (0.682,0.773) | CMI Male 0.569 (0.541,0.597)   Female  0.737 (0.691,0.782) | VAI Male 0.568 (0.54,0.596)  Female 0.735 (0.689,0.78) | LAP  Male 0.578 (0.55,0.606)  Female 0.715 (0.668,0.762) | AIP Male 0.569 (0.54,0.597)  Female 0.734 (0.688,0.779) |  |  |
| 76 | MetS | OR (95%CI) AUC (95%CI) | OR 2.742 (2.374,3.167) Male 3.637 (2.819,4.693) Femle 2.218 (1.857,2.650) AUC 0.805 (0.783,0.828) Male 0.827 (0.795,0.859) Female 0.797 (0.767,0.827) | AUC 0.745 (0.719,0.771) Male 0.781 (0.744,0.818) Female 0.722 (0.687,0.758) |  |  | LAP OR 1.065 (1.057,1.074) Male 1.054 (1.044,1.065) Femle 1.080 (1.066,1.094) AUC 0.882 (0.864,0.899) Male 0.884 (0.857,0.911) Female 0.883 (0.860,0.906) | AVI OR 1.37 (1.308,1.435) Male 1.557 (1.430,1.696) Femle 1.375 (1.283,1.474) AUC 0.781 (0.757,0.805) Male 0.838 (0.808,0.868) Female 0.794 (0.764,0.823) | BAI OR 1.15 (1.113,1.189) Male 1.263 (1.176,1.358) Femle 1.160 (1.104,1.219) AUC 0.665 (0.635,0.695) Male 0.699 (0.655,0.742) Female 0.705 (0.668,0.742) | TyG OR 21.464 (14.726,31.29) Male 14.283 (8.598,23.726) Femle 34.145 (19.087,61.08) AUC 0.854 (0.833,0.874) Male 0.841 (0.809,0.873) Female 0.866 (0.840,0.892) | VAI OR 2.804 (2.444,3.216) Male 2.52 (2.076,3.060) Femle 3.001 (2.475,3.664) AUC 0.845 (0.823,0.867) Male 0.850 (0.819,0.882) Female 0.843 (0.814,0.873) | CVAI OR 1.041 (1.036,1.046) Male 1.049 (1.040,1.058) Femle 1.053 (1.043,1.063) AUC 0.845 (0.825,0.865) Male 0.868 (0.841,0.895)  Female 0.854 (0.828,0.88) |  |  |
| 77 | Diabetes Mellitus | OR (95%CI) | OR 1.40 (1.19,1.66) |  | OR 1.42 (1.20,1.69) | OR 1.41 (1.19,1.67) |  |  |  |  |  |  |  |  |
| 78 | Type 2 Diabetes | HR (95%CI) | HR Male 1.34  (1.18, 1.52) Female 1.48  (1.26, 1.74) AUC Male 0.715 (0.684,0.746) Female 0.758 (0.703,0.814) | AUC Male 0.684  (0.651, 0.717) Female 0.757  (0.704, 0.811) | AUC Male 0.700  (0.666, 0.733) Female 0.733  (0.676, 0.789) |  |  |  |  |  |  |  |  |  |
| 79 | NAFLD | OR (95%CI) AUC (95%CI) | OR Male 3.32  (2.80, 3.22) Female 2.58  (2.32, 2.88)  AUC Male 0.8156 (0.8054,0.826) Female  0.8790 (0.8647, 0.893) | OR Male 3.35  (3.06, 3.67) Female 3.14  (2.79, 3.55)   AUC Male  0.8160 (0.8055,0.8264) Female  0.8799 (0.8648,0.8950) | OR Male 3.55 (3.22, 3.92) Female 3.31 (2.89, 3.79)  AUC Male  0.8102  (0.7998, 0.8207) Female  0.8695  (0.8545, 0.8844) | OR Male 3.50  (3.19, 3.85) Female  2.85  (2.53, 3.20)  AUC Male 0.8156  (0.805,0.8257) Female 0.8790 (0.8647,0.893) | TG: triglyceride OR Male 1.34  (1.25, 1.43) Female  1.86  (1.60, 2.18)  AUC Male  0.7367 (0.7242,0.7492) Female  0.8049 (0.7852,0.8246) | TyG OR Male 1.79 (1.63,1.95) Female 2.43 (2.06,2.86)  AUC Male  0.7458 (0.7336,0.758) Female 0.8186 (0.7998,0.837) | TyG-BMI OR Male 4.31 (3.88, 4.79) Female 4.62 (3.97, 5.38)  AUC Male 0.8428 (0.8331,0.8525) Female 0.9084 (0.8964,0.9204) | TyG-WC OR Male 4.47 (4.00,5.00) Female  5.56 (4.65,6.63)  AUC Male  0.8356 (0.8257,0.845) Female 0.9045 (0.8926,0.916) | TyG-WHtR OR Male 4.37 (3.92, 4.87) Female 4.53 (3.87, 5.29)  AUC Male 0.8372 (0.8274,0.8469) Female 0.9071 (0.8954,0.9188) | LAP OR Male 2.24 (2.07, 2.43) Female 3.41 (2.91, 4.00)  AUC Male  0.8227 (0.8126,0.8328) Female  0.8968 (0.8845,0.9092) | VAI OR Male 1.33 (1.24, 1.43) Female  1.61 (.40, 1.86)  AUC Male  0.7565 (0.7445, 0.7684) Female  0.8281 (0.8098,0.8464) | ABSI OR Male 1.29 (1.19, 1.39) Female 1.18 (1.08, 1.30)   AUC Male  0.5795 (0.5653,0.5936) Female  0.6171 (0.5916,0.6426) |
| 80 | Diabetes Mellitus | HR (95%CI) | HR 1.36 (1.28, 1.45)  AUC 0.630  (0.610,0.651) Male 0.613  (0.582, 0.643) Female 0.638  (0.611, 0.666) | HR 1.40  (1.31, 1.49)   AUC 0.620  (0.599,0.641) | HR 1.42 (1.33, 1.52)   AUC 0.623  (0.602,0.643) | HR 1.39  (1.30, 1.49)  AUC 0.630  (0.610,0.651) | CI  HR 1.18  (1.10, 1.27)  AUC 0.574  (0.553,0.595) | HC HR 1.30 (1.22, 1.38)  AUC 0.595  (0.574,0.616) | WHR HR 1.16 (1.12, 1.21)  AUC 0.574  (0.553,0.595) | ABSI HR 1.04 (0.96, 1.12)   AUC 0.531  (0.510,0.552) | AVI HR 1.40 (1.31, 1.49)   AUC 0.623  (0.602,0.644) | BAI  HR 1.27 (1.18, 1.37)   AUC 0.597  (0.576,0.618) | WWI HR 1.17 (1.08, 1.27)   AUC 0.578  (0.557,0.598) | WHHR HR 1.17 (1.10, 1.24)   AUC 0.582  (0.561,0.602) |
| 81 | MetS | AUC (95%CI) | Male 0.78 (0.74,0.81) Female 0.79 (0.75,0.82) | Male 0.76 (0.73,0.79) Female 0.76 (0.72,0.80) | Male 0.79 (0.76,0.82) Female 0.81 (0.77,0.84) | Male 0.78 (0.74,0.81) Female 0.79 (0.75,0.83) | CI,conicity index Male 0.69 (0.66,0.73) Female 0.73 (0.69,0.77) | ABSI Male 0.60 (0.57,0.64) Female 0.65 (0.61,0.69) | LAP Male 0.88 (0.86,0.91) Female 0.87 (0.85,0.9) | VAI Male 0.87 (0.84,0.89) Female  0.85 (0.81,0.88) |  |  |  |  |
| 82 | Stroke | RR (95%CI) | RR 1.04  (0.96, 1.11) Male 0.98  (0.88, 1.09) Female 1.15 (1.03, 1.27) Diabetic 0.94  (0.74, 1.19) Non‐diabetic 1.02  (0.94, 1.11) | RR 1.11  (1.02, 1.21) Male 1.06 (0.92, 1.22) Female 1.08  (0.94, 1.24) Diabetic 1.04  (0.90, 1.20) Non‐diabetic 1.11  (1.00, 1.24) | RR 1.08 (1.01, 1.17) Male 1.22 (1.09, 1.36) Female 1.02 (0.92, 1.12) Diabetic 1.03 (0.86, 1.24) Non‐diabetic 1.06 (0.97, 1.15) | RR 0.95  (0.88, 1.02) Male 0.89 (0.78, 1.02) Female 0.98  (0.88, 1.09) Diabetic 0.94  (0.76, 1.16) Non‐diabetic 0.92  (0.84, 1.01) | WHR RR 1.09  (1.01, 1.18) Male 1.14  (0.97, 1.33) Female 1.07  (0.96, 1.19) Diabetic 1.15  (0.87, 1.52) Non‐diabetic 1.04  (0.96, 1.14) | ABSI RR 1.12  (1.04, 1.20) Male 1.17  (1.04, 1.30) Female 1.15  (1.03, 1.28) Diabetic 1.15  (0.96, 1.38) Non‐diabetic 1.10  (1.02, 1.20) |  |  |  |  |  |  |
| 83 | MetS | AUC (95%CI) | Male  0.754 (0.741,0.768) Female 0.625 (0.609,0.642) |  |  |  | ABSI Male  0.491 (0.474,0.509) Female 0.492 (0.474,0.509) | VAI  Male  0.824 (0.812,0.836) Female 0.866 (0.855,0.877) |  |  |  |  |  |  |
| 84 | MetS | AUC (95%CI) | Male 0.728 (0.713,0.743) Female 0.748 (0.737,0.758) | Male 0.754 (0.739,0.769) Female 0.731 (0.720,0.742) |  | WHtR Male 0.764 (0.749,0.778) Female 0.758 (0.748,0.768) | BF Male  0.738 (0.722,0.753) Female 0.722 (0.711,0.733) | ABSI Male 0.603 (0.586,0.620) Female 0.639 (0.627,0.651) | CUN BAE Male 0.760 (0.746,0.775) Female 0.742 (0.732,0.753) |  |  |  |  |  |
| 85 | HTN | AUC (95%CI) | 0.657 (0.650,0.663) |  |  |  | ABSI 0.615 (0.608,0.621) | CI 0.541 (0.534,0.547) |  |  |  |  |  |  |
| 86 | MetS | OR (95%CI) | Male 1.986  (1.175, 3.356) Female  1.679  (1.283, 2.197) | Male 1.239  (0.674, 2.276) Female 1.008  (0.654, 1.556) | Male 3.276  (1.706, 6.293) Female  2.908  (2.000, 4.228) |  | ABSI Male 1.686  (0.958, 2.381) Female 2.206  (0.976, 2.897) | WHR Male 0.797  (0.403, 1.577) Female  0.604  (0.381, 0.957) |  |  |  |  |  |  |
| 87 | Diabetes Mellitus | AUC (95%CI) | Male 0.662  (0.638, 0.686) Female 0.726  (0.707, 0.745) | Male 0.619  (0.594, 0.643) Female 0.645  (0.623, 0.667) |  |  | ABSI Male 0.649  (0.623, 0.675) Female 0.698  (0.678, 0.718) |  |  |  |  |  |  |  |
| 88 | HTN; Hyperuricemia; NAFLD; MetS | OR (95%CI) AUC (95%CI) | HTN OR Male 1.807 (1.756,1.860) Female 1.646 (1.572,1.723) AUC Male 0.690 (0.685,0.695) Female 0.769 (0.761,0.778) Hyperuricemia OR Male 1.665 (1.617,1.714) Female 1.889 (1.782,2.002) AUC Male 0.638 (0.633,0.643) Female 0.728 (0.722,0.733) NAFLD OR Male 3.039 (2.950,3.13) Female 3.353 (3.196,3.519) AUC Male 0.757 (0.752,0.761) Female 0.832 (0.827,0.837) MetS OR Male 2.41 (2.346,2.477) Female 2.25 (2.160,2.344) AUC Male 0.727 (0.721,0.732) Female 0.782 (0.776,0.789) | HTN OR Male 1.956(1.899,2.014) Female 1.930 (1.839,2.026) AUC Male 0.667 (0.662,0.672) Female 0.738 (0.728,0.748) Hyperuricemia OR Male 1.690 (1.642,1.740) Female 1.901 (1.797,2.010) AUC Male 0.649 (0.644,0.654) Female 0.722 (0.716,0.728) NAFLD OR Male 3.181 (3.087,3.277) Female 3.705 (3.532,3.887) AUC Male 0.770 (0.766,0.775) Female 0.837 (0.833,0.842) MetS OR Male 2.423 (2.359,2.489) Female 2.241 (2.157,2.329) AUC Male 0.719 (0.714,0.724) Female 0.760 (0.753,0.767) | HTN OR Male 1.837 (1.783,1.892) Female 1.70 (1.622,1.781) AUC Male 0.690 (0.685,0.695) Female 0.752 (0.743,0.762) Hyperuricemia OR Male 1.730 (1.679,1.783) Female 1.960 (1.843,2.083) AUC Male 0.638 (0.633,0.643) Female 0.724 (0.718,0.730) NAFLD OR Male 3.117 (2.025,3.21) Female 3.579 (3.407,3.76) AUC Male 0.752 (0.752,0.761) Female 0.832 (0.827,0.836) MetS OR Male 2.399 (2.335,2.464) Female 2.254 (2.165, 2.347) AUC Male 0.721 (0.716,0.726) Female 0.770 (0.763,0.777) | HTN OR Male 1.860 (1.805,1.917) Female 1.721 (1.640,1.807) AUC Male 0.690 (0.685,0.695) Female 0.769 (0.761,0.778) Hyperuricemia OR Male 2.015 (1.891,2.147) Female 1.717 (1.666,1.77) AUC Male 0.638 (0.633,0.643) Female 0.728 (0.722,0.733) NAFLD OR Male 3.101 (3.010,3.195) Female 3.613 (3.436,3.80) AUC Male 0.752 (0.752,0.761) Female 0.832 (0.827,0.837) MetS OR Male 2.455 (2.389,2.523) Female 2.348 (2.251,2.448) AUC Male 0.727 (0.721,0.732) Female 0.782 (0.776,0.789) | ABSI HTN OR Male 1.073 (1.043,1.104) Female: NA AUC Male 0.586 (0.581,0.591) Female 0.648 (0.638,0.659) Hyperuricemia OR Male 1.170 (1.135,1.206) Female 1.204 (1.129,1.285) AUC Male 0.527 (0.527,0.538) Female 0.609 (0.603,0.615) NAFLD  OR Male 1.268 (1.240,1.297) Female 1.241 (1.195,1.29) AUC Male 0.527 (0.566,0.577) Female 0.643 (0.636,0.649) MetS OR Male 1.238 (1.120,1.267) Female 1.224 (1.180,1.269) AUC Male 0.585 (0.579,0.591) Female 0.648 (0.640,0.657) | CI HTN OR Male 1.405 (1.364,1.446) Female 1.279 (1.218,1.343) AUC Male 0.643 (0.638,0.648) Female 0.711 (0.701,0.721) Hyperuricemia OR Male 1.474 (1.428,1.521) Female 1.559 (1.458,1.667) AUC Male 0.588 (0.583,0.593) Female 0.671 (0.655,0.677) NAFLD OR Male 1.961 (1.911,2.011) Female 1.937 (1.856,2.022) AUC Male 0.666 (0.661,0.671) Female 0.738 (0.732,0.744) MetS OR Male 1.757 (1.713,1.801) Female 1.638 (1.575,1.703) AUC Male 0.660 (0.655,0.666) Female 0.655 (0.639,0.670) |  |  |  |  |  |  |
| 89 | Type 2 Diabetes | AUC (95%CI) | Male  0.629 (0.600,0.658) Female 0.609 (0.574,0.644) | Male  0.655 (0.626,0.684) Female  0.635 (0.602,0.667) | Male 0.629 (0.600,0.659) Female 0.616 (0.581,0.651) | Male  0.629 (0.600,0.658) Female  0.609 (0.574,0.644) | VAI Male  0.609 (0.578,0.639) Female  0.582 (0.548,0.617) | ABSI Male 0.507 (0.477,0.538) Female  0.503 (0.465,0.540) |  |  |  |  |  |  |
| 90 | HTN | AUC (95%CI) | 0.662 (0.625,0.700) | 0.623 (0.582,0.664) | 0.672 (0.634,0.711) | 0.662  (0.625,0.700) | ABSI 0.627 (0.587,0.667) | WHR 0.648 (0.608,0.688) |  |  |  |  |  |  |
| 91 | Hyperuricemia | OR (95%CI) AUC (95%CI) | OR Male 1.058 (1.048,1.066) Female  1.459 (1.355,1.571) AUC Male 0.602 (0.586,0.618) Female 0.656 (0.642,0.670) | OR Male 1.082 (1.061,1.103) Female 1.108 (1.089,1.127) AUC Male 0.627 (0.611,0.643) Female 0.630 (0.616,0.645) | OR Male 1.042 (1.035,1.050) Female  1.047 (1.040,1.054) AUC Male 0.647 (0.631,0.663) Female 0.658  (0.644, 0.672) | OR Male 1.061 (1.048,1.074) Female 1.067 (1.056,1.078) AUC Male 0.631 (0.615,0.646) Female 0.641 (0.626,0.655) | ABSI OR Male 1.034 (1.023,1.046) Female 1.027 (1.016,1.039) AUC Male 0.578  (0.562, 0.594) Female 0.589  (0.574, 0.604) |  |  |  |  |  |  |  |
| 92 | NAFLD | OR (95%CI) | Male 5.484  (4.572, 6.577) Female 3.482  (2.973, 4.077) | Male 5.554  (4.686, 6.582) Female 3.549  (3.064, 4.111) | Male 4.881  (4.173, 5.709) Female 4.092  (3.461, 4.838) | Male 5.309  (4.463, 6.317) Female 3.854  (3.265, 4.549) | ABSI Male 1.363  (1.208, 1.539) Female 1.003  (0.909, 1.107) | WHR Male 3.123  (2.777, 3.512) Female 1.628  (1.417, 1.870) |  |  |  |  |  |  |
| 93 | HTN; Diabetes Mellitus; Dyslipidemia; Hyperuricemia; MetS | AUC (95%CI) | Male HTN 0.668 (0.65,0.687) Diabetes 0.708 (0.679,0.736) MetS 0.71 (0.693,0.727) Dyslipidemia 0.674 (0.656,0.691) Hyperuricemia 0.637 (0.616,0.658) Female HTN 0.714 (0.698,0.73) Diabetes Mellitus 0.702 (0.671,0.733) MetS 0.703 (0.687,0.719) Dyslipidemia 0.676 (0.659,0.692) Hyperuricemia 0.658 (0.635,0.682) | Male HTN 0.639 (0.62,0.658) Diabetes 0.663 (0.633,0.693) MetS 0.717 (0.7,0.734) Dyslipidemia 0.69 (0.673,0.708) Hyperuricemia 0.648 (0.626,0.669) Female HTN 0.667 (0.649,0.686) Diabetes Mellitus 0.661 (0.629,0.694) MetS 0.692 (0.675,0.708) Dyslipidemia 0.66 (0.643,0.678) Hyperuricemia 0.633 (0.607,0.658) | Male HTN 0.662(0.643,0.68) Diabetes 0.697 (0.669,0.726) MetS 0.712 (0.695,0.729) Dyslipidemia 0.683 (0.665,0.7) Hyperuricemia 0.64 (0.619,0.662) Female HTN 0.698 (0.681.0.715) Diabetes Mellitus 0.697 (0.665,0.728) MetS 0.699 (0.683,0.715) Dyslipidemia 0.671 (0.654,0.688) Hyperuricemia 0.643 (0.619.0.667) | Male HTN 0.668 (0.65,0.687) Diabetes 0.708 (0.679,0.736) MetS 0.71 (0.693,0.727) Dyslipidemia 0.674 (0.656.0.691) Hyperuricemia 0.637 (0.616,0.658) Female HTN 0.714 (0.698,0.73) Diabetes Mellitus 0.702 (0.671,0.733) MetS 0.703 (0.687,0.719) Dyslipidemia 0.676 (0.659,0.692) Hyperuricemia 0.658  (0.635,0.682) | WHR Male HTN 0.638 (0.619,0.657) Diabetes 0.685 (0.657,0.713) MetS 0.672 (0.654,0.689) Dyslipidemia 0.654 (0.636,0.672) Hyperuricemia 0.617 (0.596,0.638) Female HTN 0.656 (0.639,0.674) Diabetes Mellitus 0.677 (0.646,0.707) MetS 0.657 (0.641,0.674) Dyslipidemia 0.649 (0.632,0.666) Hyperuricemia 0.626 (0.602,0.65) | ABSI Male HTN 0.597 (0.578,0.616) Diabetes 0.635 (0.605,0.666) MetS 0.572 (0.553,0.591) Dyslipidemia 0.551 (0.532,0.57) Hyperuricemia 0.541 (0.518,0.563) Female HTN 0.628 (0.61,0.646) Diabetes Mellitus 0.631 (0.599,0.664) MetS 0.586 (0.569,0.604) Dyslipidemia 0.584 (0.566,0.602) Hyperuricemia 0.58 (0.556,0.605) |  |  |  |  |  |  |
| Abbreviations: NA, not applicable; NAFLD, Non-alcoholic fatty liver disease; MetS, Metabolic Syndrome; CKD, Chronic Kidney Disease; MAFLD, Metabolic dysfunction-associated fatty liver disease; CVD, Cardiovascular Diseases; CHD, Coronary Heart Disease; HTN, Hypertension; OR, odds ratio; RR, relative risk; HR, hazard ratios; AUC, Area Under the Curve; 95% CI, 95% confidence interval; BMI, body mass index; WC, waist circumference; WHtR, waist-to-height ratio; BRI, body roundness index; CI, conicity index; LAP, lipid accumulation product index; VAI, visceral adiposity index; CVAI, Chinese visceral adiposity index; CUN-BAE, Clínica Universidad de Navarra-Body Adiposity Estimator; ABSI, A body shape index; BF, Body Fat Ratio; WHR, Waist-to-Hip Ratio; BAI, Body Adiposity Index; HC, hip circumference; CMI, cardiometabolic index; WWI, weight-adjusted-waist; AVI, Abdominal Volume Index; TyG, Triglyceride-glucose; HSI, hepatic steatosis index; TG/HDL-C, triglyceride to high-density lipoprotein cholesterol ratio; TyG-BMI,TyG related to BMI; TyG-WC,TyG related to WC; TyG-WHtR,TyG related to WHtR; NC, Neck Circumference; AIP, atherogenic index of plasma; WHHR, waist-hip-height ratio; | | | | | | | | | | | | | | |

| Suppl. Table 3 Pooled accuracy parameters in the prediction of CKM Syndrome-related diseases. | | | | | | | | | |
| --- | --- | --- | --- | --- | --- | --- | --- | --- | --- |
| Subgroup | Indicator | N | Sensitivity | Specificity | PLR | NLR | dOR | AUC-SROC | Publication Bias (p-Value) |
| **Metabolic Syndrome** | | | | | | | | | |
| Male | BRI | 6 | 0.82(0.71, 0.89) | 0.73(0.64, 0.81) | 3.1(2.1, 4.4) | 0.25(0.15, 0.42) | 12(5, 28) | 0.84(0.80, 0.87) | 0.13 |
| Female | BRI | 6 | 0.80(0.69, 0.88) | 0.68(0.61, 0.74) | 2.5(2.2, 2.9) | 0.30(0.20, 0.44) | 8(6, 13) | 0.78(0.74, 0.81) | 0.47 |
| **Hypertension** | | | | | | | | | |
| Total | BRI | 6 | 0.63(0.58, 0.68) | 0.57(0.46, 0.68) | 1.5(1.2, 1.8) | 0.64(0.57, 0.72) | 2(2, 3) | 0.64(0.60, 0.68) | 0.8 |
|  | ABSI | 6 | 0.70(0.55, 0.82) | 0.41(0.24, 0.60) | 1.2(1.0, 1.4) | 0.73(0.63, 0.84) | 2(1, 2) | 0.60(0.56, 0.64) | 0.62 |
| Male | BRI | 7 | 0.64(0.55, 0.73) | 0.63(0.56, 0.69) | 1.7(1.5, 2.0) | 0.57(0.47, 0.69) | 3(2, 4) | 0.67(0.63, 0.71) | 0.96 |
|  | BMI | 7 | 0.61(0.55, 0.66) | 0.60(0.55, 0.65) | 1.5(1.4, 1.6) | 0.66(0.60, 0.72) | 2(2, 3) | 0.64(0.59, 0.68) | 0.28 |
|  | WC | 5 | 0.59(0.54, 0.64) | 0.65(0.59, 0.69) | 1.7(1.5, 1.9) | 0.64(0.58, 0.69) | 3(2, 3) | 0.65(0.61, 0.69) | 0.68 |
|  | WHtR | 7 | 0.65(0.56, 0.73) | 0.61(0.55, 0.67) | 1.7(1.5, 1.9) | 0.57(0.48, 0.69) | 3(2, 4) | 0.67(0.62, 0.71) | 0.79 |
|  | ABSI | 6 | 0.63(0.61, 0.66) | 0.55(0.47, 0.63) | 1.4(1.2, 1.7) | 0.66(0.58, 0.76) | 2(2, 3) | 0.64(0.60, 0.68) | 0.16 |
|  | CI | 6 | 0.64(0.59, 0.69) | 0.58(0.50, 0.66) | 1.5(1.3, 1.8) | 0.62(0.54, 0.70) | 2(2, 3) | 0.65(0.61, 0.69) | 0.78 |
| Female | BRI | 7 | 0.65(0.61, 0.70) | 0.65(0.60, 0.70) | 1.9(1.6, 2.2) | 0.53(0.46, 0.62) | 4(3, 5) | 0.70(0.66, 0.74) | 0.61 |
|  | BMI | 7 | 0.63(0.56, 0.69) | 0.63(0.60, 0.65) | 1.7(1.5, 1.9) | 0.59(0.50, 0.70) | 3(2, 4) | 0.66(0.62, 0.70) | 0.55 |
|  | WC | 5 | 0.66(0.57, 0.73) | 0.63(0.61, 0.65) | 1.8(1.6, 2.0) | 0.54(0.44, 0.67) | 3(2, 4) | 0.66(0.62, 0.70) | 0.59 |
|  | WHtR | 7 | 0.65(0.60, 0.69) | 0.65(0.60, 0.70) | 1.9(1.6, 2.2) | 0.54(0.47, 0.62) | 3(3, 5) | 0.70(0.65, 0.73) | 0.51 |
|  | ABSI | 6 | 0.58(0.45, 0.70) | 0.66(0.55, 0.75) | 1.7(1.4, 2.0) | 0.64(0.52, 0.79) | 3(2, 4) | 0.66(0.62, 0.70) | 0.56 |
|  | CI | 6 | 0.60(0.55, 0.65) | 0.66(0.61, 0.71) | 1.8(1.4, 2.2) | 0.60(0.50, 0.72) | 3(2, 4) | 0.67(0.63, 0.71) | 0.89 |
| **Hyperuricemia** | | | | | | | | | |
| Total | BRI | 5 | 0.62(0.56, 0.67) | 0.57(0.50, 0.64) | 1.4(1.3, 1.6) | 0.67(0.61, 0.73) | 2(2, 3) | 0.63(0.59, 0.67) | 0.86 |
| Male | BRI | 8 | 0.71(0.61, 0.79) | 0.48(0.36, 0.59) | 1.4(1.2, 1.5) | 0.61(0.55, 0.68) | 2(2, 3) | 0.64(0.59, 0.68) | 0.71 |
|  | BMI | 8 | 0.66(0.61, 0.71) | 0.52(0.46, 0.59) | 1.4(1.3, 1.5) | 0.64(0.58, 0.71) | 2(2, 3) | 0.63(0.59, 0.68) | 1.0 |
|  | WC | 7 | 0.66(0.60, 0.72) | 0.52(0.46, 0.59) | 1.4(1.3, 1.5) | 0.64(0.56, 0.74) | 2(2, 3) | 0.63(0.58, 0.67) | 0.82 |
|  | WHtR | 7 | 0.72(0.57, 0.84) | 0.45(0.30, 0.62) | 1.3(1.2, 1.5) | 0.61(0.53, 0.70) | 2(2, 2) | 0.63(0.58, 0.67) | 0.90 |
|  | ABSI | 5 | 0.62(0.55, 0.69) | 0.42(0.36, 0.49) | 1.1(1.0, 1.2) | 0.89(0.81, 0.99) | 1(1, 1) | 0.53(0.48, 0.57) | 0.29 |
|  | LAP | 5 | 0.65(0.60, 0.69) | 0.60(0.50, 0.70) | 1.6(1.4, 1.9) | 0.59(0.56, 0.61) | 3(2, 3) | 0.67(0.63, 0.71) | 0.84 |
| Female | BRI | 8 | 0.67(0.62, 0.72) | 0.59(0.54, 0.64) | 1.7(1.5, 1.8) | 0.55(0.49, 0.62) | 3(3, 4) | 0.67(0.63, 0.71) | 0.91 |
|  | BMI | 8 | 0.65(0.57, 0.72) | 0.62(0.56, 0.68) | 1.7(1.6, 1.8) | 0.57(0.49, 0.66) | 3(2, 4) | 0.67(0.63, 0.71) | 0.81 |
|  | WC | 7 | 0.68(0.62, 0.73) | 0.59(0.54, 0.64) | 1.7(1.5, 1.8) | 0.54(0.48, 0.61) | 3(3, 4) | 0.67(0.63, 0.71) | 0.28 |
|  | WHtR | 7 | 0.73(0.64, 0.81) | 0.51(0.37, 0.65) | 1.5(1.2, 1.8) | 0.52(0.46, 0.60) | 3(2, 4) | 0.69(0.65, 0.73) | 0.85 |
|  | ABSI | 5 | 0.68(0.66, 0.70) | 0.44(0.39, 0.49) | 1.2(1.1, 1.3) | 0.73(0.67, 0.78) | 2(1, 2) | 0.64(0.60, 0.68) | 0.70 |
|  | LAP | 5 | 0.66(0.61, 0.71) | 0.69(0.63, 0.74) | 2.1(1.8, 2.4) | 0.49(0.44, 0.55) | 4(4, 5) | 0.72(0.68, 0.76) | 0.93 |
| Abbreviations: CKM, Cardiovascular-Kidney-Metabolic; N, Number; Se, Sensitivity; Sp, Specificity; PLR, Positive Likelihood Ratio; NLR, Negative Likelihood Ratio; dOR, Diagnostic Odds Ratio; AUC-SROC, Area Under the Summary Receiver Operating Characteristic Curve; BMI, body mass index; WC, waist circumference; WHtR, waist-to-height ratio; BRI, body roundness index; CI, conicity index; LAP, lipid accumulation product index; ABSI, A body shape index; | | | | | | | | | |
|  |  |  |  |  |  |  |  |  |  |
|  |  |  |  |  |  |  |  |  |  |

| Suppl. Table 4 The association between different anthropometric indices and the risk of CKM Syndrome-related diseases. | | | | | | | | | | |  |
| --- | --- | --- | --- | --- | --- | --- | --- | --- | --- | --- | --- |
| Subgroup | Indicator | No.study | Outcome Assessment | Effect (95% CI) | Test of heterogeneity | | Egger's test | Sensitivity Analysis | Effect (Excl.)  (95% CI) | I^2^ (Excl.) |  |
|  |  |  |  |  | P value | I^2^ |  |  |  |  |  |
| **Metabolic Syndrome** | | | | | | | | | | |  |
| Total | BRI | 3 | OR | 2.23 (1.73, 2.88) | 0.008 | 79.30% | 0.213 | Duan et al.,2022 | 1.98(1.68, 2.33) | 0 |  |
|  | BMI | 2 | OR | 1.23 (1.17, 1.30) | 0.375 | 0 | NA | NA | NA | NA |  |
|  | ABSI | 2 | OR | 1.15 (1.08, 1.22) | 0.753 | 0 | NA | NA | NA | NA |  |
|  | LAP | 2 | OR | 1.06 (1.05, 1.07) | 0.115 | 59.80% | NA | NA | NA | NA |  |
| Male | BRI | 4 | OR | 2.82 (2.06, 3.84) | 0.006 | 76.10% | 0.403 | Duan et al.,2022 | 2.43(1.93, 3.07) | 24.40% |  |
|  | BMI | 2 | OR | 1.86 (0.98, 3.54) | 0.031 | 78.50% | NA | NA | NA | NA |  |
|  | WC | 3 | OR | 2.00 (1.10, 3.62) | 0.001 | 99.10% | 0.864 | Mungvongsa et al.,2025 | 2.40(2.34, 2.47) | 0 |  |
|  | WHtR | 2 | OR | 3.54 (1.65, 7.63) | 0.001 | 93.50% | NA | NA | NA | NA |  |
|  | VAI | 2 | OR | 3.94 (1.23, 12.61) | 0.063 | 71.00% | NA | NA | NA | NA |  |
|  | ABSI | 3 | OR | 2.46 (0.73, 8.28) | 0.001 | 98.70% | 0.538 | Mungvongsa et al.,2025 | 1.33(1.03, 1.71) | 42.40% |  |
| Female | BRI | 4 | OR | 2.27 (1.90, 2.71) | 0.041 | 63.80% | 0.818 | Robust | NA | NA |  |
|  | BMI | 2 | OR | 1.55 (0.71, 3.38) | 0.001 | 92.30% | NA | NA | NA | NA |  |
|  | WC | 3 | OR | 1.94 (1.14, 3.30) | 0.001 | 99.60% | 0.773 | Mungvongsa et al.,2025 | 2.39(1.94, 2.94) | 43.20% |  |
|  | WHtR | 2 | OR | 4.01 (1.40, 11.49) | 0.001 | 99.70% | NA | NA | NA | NA |  |
|  | VAI | 2 | OR | 5.20 (1.29, 21.03) | 0.051 | 73.80% | NA | NA | NA | NA |  |
|  | ABSI | 3 | OR | 2.19 (1.03, 4.67) | 0.001 | 85.60% | 0.180 | Mungvongsa et al.,2025 | 1.54(0.88, 2.70) | 77.70% |  |
| Total | BRI | 4 | AUC | 0.80 (0.75, 0.85) | 0.001 | 89.50% | 0.473 | Ensan et al.,2025 | 0.82(0.79, 0.84) | 16.70% |  |
|  | BMI | 4 | AUC | 0.79 (0.75, 0.82) | 0.008 | 74.70% | 0.306 | Duan et al.,2022 | 0.80(0.77, 0.84) | 54.10% |  |
|  | WC | 2 | AUC | 0.80 (0.70, 0.90) | 0.001 | 93.60% | NA | NA | NA | NA |  |
|  | WHtR | 2 | AUC | 0.78 (0.68, 0.88) | 0.001 | 91.00% | NA | NA | NA | NA |  |
|  | ABSI | 3 | AUC | 0.59 (0.49, 0.71) | 0.001 | 92.10% | 0.220 | Ensan et al.,2025 | 0.64(0.58, 0.72) | 38.90% |  |
|  | AVI | 2 | AUC | 0.76 (0.74, 0.80) | 0.051 | 73.70% | NA | NA | NA | NA |  |
|  | VAI | 2 | AUC | 0.84 (0.82, 0.87) | 0.795 | 0 | NA | NA | NA | NA |  |
|  | LAP | 2 | AUC | 0.89 (0.86, 0.93) | 0.114 | 60.00% | NA | NA | NA | NA |  |
|  | BAI | 2 | AUC | 0.67 (0.65, 0.69) | 0.786 | 0 | NA | NA | NA | NA |  |
| Male | BRI | 9 | AUC | 0.78 (0.72, 0.85) | 0.001 | 99.50% | 0.986 | Robust | NA | NA |  |
|  | BMI | 6 | AUC | 0.75 (0.73, 0.77) | 0.001 | 91.30% | 0.065 | Robust | NA | NA |  |
|  | WC | 4 | AUC | 0.73 (0.71, 0.76) | 0.001 | 87.20% | 0.410 | Zhou et al.,2020 | 0.72(0.72, 0.73) | 0 |  |
|  | WHtR | 5 | AUC | 0.74 (0.72, 0.76) | 0.001 | 89.20% | 0.494 | Suliga et al.,2019 | 0.73(0.71, 0.75) | 77.20% |  |
|  | WHR | 2 | AUC | 0.66 (0.62, 0.69) | 0.014 | 83.40% | NA | NA | NA | NA |  |
|  | AVI | 2 | AUC | 0.78 (0.68, 0.89) | 0.001 | 97.40% | NA | NA | NA | NA |  |
|  | BAI | 2 | AUC | 0.69 (0.67, 0.70) | 0.506 | 0 | NA | NA | NA | NA |  |
|  | LAP | 2 | AUC | 0.88 (0.86, 0.90) | 0.831 | 0 | NA | NA | NA | NA |  |
|  | VAI | 3 | AUC | 0.85 (0.82, 0.88) | 0.003 | 83.10% | 0.382 | Baveicy et al.,2020 | 0.86(0.84, 0.88) | 0 |  |
|  | ABSI | 7 | AUC | 0.56 (0.53, 0.59) | 0.001 | 95.90% | 0.572 | Baveicy et al.,2020 | 0.57(0.55, 0.60) | 92.40% |  |
|  | CI | 3 | AUC | 0.70 (0.64, 0.77) | 0.001 | 95.90% | 0.410 | Somdee et al.,2023 | 0.67(0.64, 0.70) | 65.60% |  |
| Female | BRI | 9 | AUC | 0.75 (0.72, 0.79) | 0.001 | 98.60% | 0.376 | Baveicy et al.,2020 | 0.77(0.74, 0.81) | 97.50% |  |
|  | BMI | 6 | AUC | 0.72 (0.70, 0.75) | 0.001 | 93.00% | 0.188 | Tian et al.,2016 | 0.73(0.71, 0.76) | 87.20% |  |
|  | WC | 4 | AUC | 0.74 (0.69, 0.79) | 0.001 | 96.10% | 0.557 | Robust | NA | NA |  |
|  | WHtR | 5 | AUC | 0.74 (0.71, 0.78) | 0.001 | 95.60% | 0.290 | Tian et al.,2016 | 0.76(0.74, 0.79) | 89.00% |  |
|  | WHR | 2 | AUC | 0.66 (0.64, 0.67) | 0.478 | 0 | NA | NA | NA | NA |  |
|  | AVI | 2 | AUC | 0.72 (0.60, 0.86) | 0.001 | 98.80% | NA | NA | NA | NA |  |
|  | BAI | 2 | AUC | 0.66 (0.56, 0.76) | 0.008 | 85.80% | NA | NA | NA | NA |  |
|  | LAP | 2 | AUC | 0.88 (0.86, 0.89) | 0.452 | 0 | NA | NA | NA | NA |  |
|  | VAI | 3 | AUC | 0.86 (0.85, 0.87) | 0.001 | 20.20% | 0.226 | Robust | NA | NA |  |
|  | ABSI | 7 | AUC | 0.57 (0.53, 0.62) | 0.001 | 97.90% | 0.118 | Baveicy et al.,2020 | 0.59(0.56, 0.63) | 95.60% |  |
|  | CI | 3 | AUC | 0.67 (0.62, 0.72) | 0.001 | 88.30% | 0.724 | Zhou et al.,2020 | 0.65(0.62, 0.67) | 51.30% |  |
| **Type 2 Diabetes** | | | | | | | | | | |  |
| Total | BRI | 6 | OR | 1.43 (1.26, 1.62) | 0.001 | 83.40% | 0.003 | Feng et al.,2024a; Zhou et al.,2025 | 1.63(1.47, 1.82) | 0 |  |
|  | BMI | 4 | OR | 1.37 (1.13, 1.67) | 0.014 | 71.80% | 0.081 | Robust | NA | NA |  |
|  | WC | 3 | OR | 1.54 (1.07, 2.22) | 0.001 | 93.60% | 0.468 | Feng et al.,2024a | 1.85(1.61,2.13) | 0 |  |
|  | WHtR | 3 | OR | 1.56 (1.14, 2.15) | 0.001 | 93.80% | 0.277 | Feng et al.,2024a | 1.82(1.60,2.06) | 0 |  |
|  | ABSI | 4 | OR | 1.24 (0.98, 1.56) | 0.001 | 85.40% | 0.827 | Feng et al.,2024a | 1.33(1.02, 1.73) | 67.40% |  |
|  | WHR | 4 | OR | 1.43 (1.10, 1.86) | 0.001 | 88.40% | 0.821 | Feng et al.,2024a | 1.54(1.14, 2.08) | 73.00% |  |
|  | AVI | 3 | OR | 1.43 (1.02, 1.99) | 0.001 | 84.50% | 0.243 | Feng et al.,2024a | 1.70(1.37,2.11) | 0 |  |
|  | BAI | 3 | OR | 1.04 (0.83, 1.29) | 0.005 | 80.80% | 0.879 | Sadeghi et al.,2024 | 0.94(0.79, 1.11) | 59.30% |  |
|  | VAI | 3 | OR | 1.32 (1.11, 1.57) | 0.088 | 58.80% | 0.369 | Robust | NA | NA |  |
| Male | BRI | 4 | OR | 1.42 (1.17, 1.72) | 0.001 | 90.20% | 0.051 | Chung et al.,2023 | 1.53(1.27, 1.86) | 73.50% |  |
|  | BMI | 2 | OR | 1.60 (1.37, 1.86) | 0.194 | 40.70% | NA | NA | NA | NA |  |
|  | CI | 2 | OR | 1.60 (1.24, 2.05) | 0.081 | 67.10% | NA | NA | NA | NA |  |
|  | WHtR | 2 | OR | 2.14 (1.78, 2.57) | 0.235 | 29.10% | NA | NA | NA | NA |  |
| Female | BRI | 4 | OR | 1.62 (1.13, 2.33) | 0.001 | 96.00% | 0.051 | Chung et al.,2023 | 1.86(1.39, 2.50) | 83.50% |  |
|  | BMI | 2 | OR | 1.82 (1.04, 3.20) | 0.001 | 95.80% | NA | NA | NA | NA |  |
|  | CI | 2 | OR | 1.98 (1.63, 2.41) | 0.371 | 0 | NA | NA | NA | NA |  |
|  | WHtR | 2 | OR | 1.99 (1.44, 2.76) | 0.032 | 78.30% | NA | NA | NA | NA |  |
| Total | BRI | 3 | AUC | 0.65 (0.57, 0.74) | 0.001 | 95.30% | 0.931 | Sekgala et al.,2024 | 0.61(0.60, 0.63) | 0 |  |
|  | BMI | 3 | AUC | 0.63 (0.58, 0.69) | 0.001 | 90.70% | 0.928 | Sekgala et al.,2024 | 0.60(0.59, 0.62) | 0 |  |
|  | WC | 3 | AUC | 0.65 (0.58, 0.73) | 0.001 | 93.60% | 0.900 | Sekgala et al.,2024 | 0.62(0.60, 0.63) | 0 |  |
|  | WHtR | 2 | AUC | 0.66 (0.57, 0.78) | 0.001 | 97.60% | NA | NA | NA | NA |  |
|  | ABSI | 2 | AUC | 0.58 (0.52, 0.64) | 0.001 | 92.80% | NA | NA | NA | NA |  |
|  | BAI | 2 | AUC | 0.56 (0.44, 0.71) | 0.001 | 98.50% | NA | NA | NA | NA |  |
|  | LAP | 2 | AUC | 0.64 (0.63, 0.66) | 0.695 | 0 | NA | NA | NA | NA |  |
|  | VAI | 2 | AUC | 0.62 (0.61, 0.64) | 0.863 | 0 | NA | NA | NA | NA |  |
|  | WHR | 2 | AUC | 0.66 (0.61, 0.71) | 0.003 | 89.00% | NA | NA | NA | NA |  |
|  | CI | 2 | AUC | 0.63 (0.54, 0.75) | 0.001 | 98.10% | NA | NA | NA | NA |  |
|  | AVI | 3 | AUC | 0.65 (0.58, 0.73) | 0.001 | 95.40% | 0.977 | Sekgala et al.,2024 | 0.61(0.60, 0.63) | 0 |  |
| Male | BRI | 6 | AUC | 0.65 (0.62, 0.68) | 0.001 | 84.30% | 0.511 | Robust | NA | NA |  |
|  | BMI | 6 | AUC | 0.63 (0.59, 0.66) | 0.001 | 85.10% | 0.197 | Robust | NA | NA |  |
|  | WC | 4 | AUC | 0.63 (0.59, 0.67) | 0.001 | 84.60% | 0.796 | Wu et al.,2022c | 0.61(0.59, 0.64) | 56.80% |  |
|  | WHtR | 5 | AUC | 0.64 (0.61, 0.67) | 0.001 | 81.80% | 0.345 | Chung et al.,2023 | 0.63(0.61, 0.65) | 39.00% |  |
|  | ABSI | 5 | AUC | 0.57 (0.53, 0.61) | 0.001 | 87.70% | 0.990 | Yang et al.,2018 | 0.59(0.55, 0.63) | 78.80% |  |
|  | VAI | 5 | AUC | 0.62 (0.58, 0.65) | 0.001 | 85.80% | 0.083 | Wang et al.,2024 | 0.63(0.60, 0.66) | 78.40% |  |
|  | LAP | 3 | AUC | 0.64 (0.59, 0.70) | 0.001 | 91.90% | 0.221 | Wang et al.,2024 | 0.67(0.64, 0.71) | 77.80% |  |
|  | CI | 3 | AUC | 0.61 (0.58, 0.63) | 0.057 | 65.10% | 0.390 | Wang et al.,2024 | 0.62(0.60, 0.64) | 33.40% |  |
| Female | BRI | 6 | AUC | 0.66 (0.62, 0.70) | 0.001 | 92.40% | 0.925 | Robust | NA | NA |  |
|  | BMI | 6 | AUC | 0.65 (0.60, 0.69) | 0.001 | 92.10% | 0.831 | Robust | NA | NA |  |
|  | WC | 4 | AUC | 0.64 (0.60, 0.67) | 0.01 | 73.40% | 0.166 | Wu et al.,2022c | 0.62(0.60, 0.64) | 31.10% |  |
|  | WHtR | 5 | AUC | 0.65 (0.61, 0.69) | 0.001 | 93.50% | 0.208 | Chung et al.,2023 | 0.64(0.60, 0.68) | 87.80% |  |
|  | CI | 3 | AUC | 0.62 (0.60, 0.63) | 0.211 | 35.70% | 0.341 | Robust | NA | NA |  |
|  | ABSI | 5 | AUC | 0.59 (0.53, 0.66) | 0.001 | 95.50% | 0.809 | Robust | NA | NA |  |
|  | VAI | 5 | AUC | 0.64 (0.60, 0.69) | 0.001 | 92.80% | 0.015 | Yang et al.,2018 | 0.66(0.62, 0.70) | 91.60% |  |
|  | LAP | 3 | AUC | 0.68 (0.63, 0.75) | 0.001 | 95.80% | 0.299 | Chung et al.,2023 | 0.66(0.63, 0.68) | 55.90% |  |
| **Diabetes Mellitus** | | | | | | | | | | |  |
| Total | BRI | 6 | OR | 1.31 (1.15, 1.50) | 0.001 | 98.20% | 0.533 | Zhang et al.,2025a | 1.36(1.25, 1.47) | 86.10% |  |
|  | BMI | 2 | OR | 1.39 (1.37, 1.41) | 0.833 | 0 | NA | NA | NA | NA |  |
|  | LAP | 2 | HR | 1.31 (1.27, 1.35) | 0.382 | 0 | NA | NA | NA | NA |  |
|  | WC | 4 | OR | 1.49 (1.46, 1.52) | 0.374 | 3.80% | 0.095 | Robust | NA | NA |  |
|  | WHtR | 5 | OR | 1.46 (1.43, 1.49) | 0.42 | 0 | 0.215 | Robust | NA | NA |  |
|  | ABSI | 2 | HR | 1.08 (1.01, 1.15) | 0.22 | 33.60% | NA | NA | NA | NA |  |
|  | VAI | 2 | HR | 1.16 (1.03, 1.31) | 0.001 | 91.90% | NA | NA | NA | NA |  |
| Male | BRI | 2 | OR | 1.36 (1.00, 1.86) | 0.004 | 88.10% | NA | NA | NA | NA |  |
| Female | BRI | 2 | OR | 1.25 (1.14, 1.38) | 0.137 | 54.70% | NA | NA | NA | NA |  |
| Total | BRI | 3 | AUC | 0.64 (0.57, 0.72) | 0.001 | 98.00% | 0.35 | Yin et al.,2025 | 0.68(0.67, 0.70) | 0 |  |
|  | BMI | 2 | AUC | 0.63 (0.52, 0.75) | 0.001 | 98.40% | NA | NA | NA | NA |  |
|  | WHtR | 3 | AUC | 0.64 (0.58, 0.70) | 0.001 | 96.80% | 0.425 | Yin et al.,2025 | 0.68(0.66, 0.69) | 0 |  |
|  | WHR | 2 | AUC | 0.61 (0.53, 0.70) | 0.001 | 97.30% | NA | NA | NA | NA |  |
|  | BAI | 2 | AUC | 0.58 (0.51, 0.66) | 0.001 | 95.80% | NA | NA | NA | NA |  |
|  | ABSI | 3 | AUC | 0.57 (0.46, 0.71) | 0.001 | 99.10% | 0.179 | Zhang et al.,2025a | 0.51(0.49, 0.54) | 75.00% |  |
| Male | BRI | 4 | AUC | 0.69 (0.66, 0.72) | 0.001 | 81.50% | 0.111 | Liu et al.,2023 | 0.68(0.65, 0.70) | 74.20% |  |
|  | BMI | 4 | AUC | 0.67 (0.63, 0.72) | 0.001 | 89.30% | 0.659 | Zhao et al.,2018 | 0.69(0.66, 0.73) | 77.60% |  |
|  | WC | 3 | AUC | 0.69 (0.66, 0.72) | 0.022 | 73.80% | 0.125 | Robust | NA | NA |  |
|  | WHtR | 3 | AUC | 0.70 (0.66, 0.75) | 0.001 | 86.00% | 0.097 | Robust | NA | NA |  |
|  | WHR | 2 | AUC | 0.66 (0.60, 0.71) | 0.001 | 91.50% | NA | NA | NA | NA |  |
|  | ABSI | 4 | AUC | 0.59 (0.51, 0.68) | 0.001 | 97.30% | 0.782 | Ensan et al.,2025 | 0.63(0.61, 0.66) | 43.00% |  |
| Female | BRI | 4 | AUC | 0.71 (0.70, 0.73) | 0.334 | 11.70% | 0.308 | Robust | NA | NA |  |
|  | BMI | 4 | AUC | 0.66 (0.64, 0.67) | 0.377 | 3.20% | 0.567 | Robust | NA | NA |  |
|  | WC | 3 | AUC | 0.69 (0.67, 0.71) | 0.536 | 0 | 0.073 | Robust | NA | NA |  |
|  | WHtR | 3 | AUC | 0.70 (0.68, 0.72) | 0.654 | 0 | 0.279 | Robust | NA | NA |  |
|  | WHR | 2 | AUC | 0.68 (0.65, 0.70) | 0.944 | 0 | NA | NA | NA | NA |  |
|  | ABSI | 4 | AUC | 0.62 (0.56, 0.68) | 0.001 | 93.70% | 0.014 | Zhao et al.,2018 | 0.59(0.55, 0.64) | 75.60% |  |
| **Dyslipidemia** | | | | | | | | | | |  |
| Total | BRI | 3 | AUC | 0.59 (0.55, 0.64) | 0.001 | 96.50% | 0.444 | Ensan et al.,2025 | 0.62(0.58, 0.66) | 97.20% |  |
|  | BMI | 3 | AUC | 0.60 (0.56, 0.65) | 0.001 | 97.30% | 0.366 | Ensan et al.,2025 | 0.63(0.59, 0.68) | 97.30% |  |
|  | WC | 2 | AUC | 0.59 (0.48, 0.72) | 0.001 | 97.80% | NA | NA | NA | NA |  |
|  | WHtR | 3 | AUC | 0.61 (0.57, 0.64) | 0.001 | 94.10% | 0.204 | Ensan et al.,2025 | 0.63(0.62, 0.65) | 78.60% |  |
|  | WHR | 3 | AUC | 0.58 (0.55, 0.62) | 0.001 | 95.80% | 0.075 | Ensan et al.,2025 | 0.62(0.61, 0.63) | 0 |  |
|  | ABSI | 3 | AUC | 0.54 (0.51, 0.59) | 0.001 | 95.90% | 0.499 | Yin et al.,2025 | 0.53(0.52, 0.54) | 0 |  |
|  | BF | 2 | AUC | 0.57 (0.52, 0.63) | 0.001 | 98.20% | NA | NA | NA | NA |  |
|  | BAI | 3 | AUC | 0.56 (0.52, 0.59) | 0.001 | 93.60% | 0.879 | Feng et al.,2024a | 0.57(0.57, 0.58) | 0 |  |
|  | CI | 2 | AUC | 0.60 (0.57, 0.63) | 0.001 | 94.90% | NA | NA | NA | NA |  |
|  | AVI | 2 | AUC | 0.58 (0.47, 0.72) | 0.001 | 98.20% | NA | NA | NA | NA |  |
| Male | BRI | 2 | AUC | 0.60 (0.48, 0.75) | 0.001 | 98.50% | NA | NA | NA | NA |  |
|  | BMI | 2 | AUC | 0.60 (0.46, 0.79) | 0.001 | 98.70% | NA | NA | NA | NA |  |
|  | WC | 2 | AUC | 0.60 (0.47, 0.77) | 0.001 | 98.60% | NA | NA | NA | NA |  |
|  | WHtR | 2 | AUC | 0.60 (0.48, 0.75) | 0.001 | 98.10% | NA | NA | NA | NA |  |
|  | WHR | 2 | AUC | 0.57 (0.44, 0.74) | 0.001 | 98.60% | NA | NA | NA | NA |  |
|  | ABSI | 2 | AUC | 0.54 (0.51, 0.57) | 0.063 | 71.10% | NA | NA | NA | NA |  |
| Female | BRI | 2 | AUC | 0.60 (0.48, 0.75) | 0.001 | 98.60% | NA | NA | NA | NA |  |
|  | BMI | 2 | AUC | 0.63 (0.54, 0.72) | 0.072 | 69.20% | NA | NA | NA | NA |  |
|  | WC | 2 | AUC | 0.62 (0.51, 0.75) | 0.021 | 81.20% | NA | NA | NA | NA |  |
|  | WHtR | 2 | AUC | 0.61 (0.49, 0.77) | 0.008 | 85.60% | NA | NA | NA | NA |  |
|  | WHR | 2 | AUC | 0.63 (0.57, 0.70) | 0.150 | 51.80% | NA | NA | NA | NA |  |
|  | ABSI | 2 | AUC | 0.56 (0.50, 0.63) | 0.154 | 50.70% | NA | NA | NA | NA |  |
| **Hyperuricemia** | | | | | | | | | | |  |
| Total | BRI | 3 | OR | 1.45 (1.19, 1.75) | 0.001 | 99.50% | 0.500 | Su et al.,2023 | 1.54(1.33, 1.79) | 96.40% |  |
|  | BMI | 3 | OR | 1.38 (1.07, 1.78) | 0.001 | 99.70% | 0.285 | Su et al.,2023 | 1.52(1.34, 1.71) | 94.50% |  |
|  | WHtR | 2 | OR | 1.24 (0.95, 1.61) | 0.001 | 99.00% | NA | NA | NA | NA |  |
|  | ABSI | 2 | OR | 1.10 (1.00, 1.21) | 0.001 | 90.70% | NA | NA | NA | NA |  |
|  | WWI | 2 | OR | 1.30 (1.04, 1.63) | 0.001 | 98.20% | NA | NA | NA | NA |  |
| Male | BRI | 4 | OR | 1.32 (1.10, 1.59) | 0.001 | 99.80% | 0.272 | Zhang et al.,2018 | 1.21(1.04, 1.41) | 99.60% |  |
|  | BMI | 4 | OR | 1.37 (1.05, 1.79) | 0.001 | 99.60% | 0.381 | Zhang et al.,2018 | 1.26(1.09, 1.45) | 93.10% |  |
|  | WC | 3 | OR | 1.56 (1.36, 1.79) | 0.001 | 90.80% | 0.340 | Zhang et al.,2018 | 1.47(1.38, 1.56) | 0 |  |
|  | VAI | 2 | OR | 1.24 (1.22, 1.26) | 0.353 | 0 | NA | NA | NA | NA |  |
|  | AVI | 2 | OR | 1.25 (0.98, 1.59) | 0.002 | 89.30% | NA | NA | NA | NA |  |
|  | ABSI | 3 | OR | 1.14 (1.01, 1.30) | 0.001 | 98.40% | 0.589 | Zhang et al.,2016 | 1.21(1.12, 1.30) | 77.00% |  |
|  | WHtR | 3 | OR | 1.46 (0.97, 2.19) | 0.001 | 99.50% | 0.272 | Zhang et al.,2018 | 1.24(0.94, 1.23) | 98.20% |  |
|  | CI | 2 | OR | 1.47 (1.43, 1.50) | 0.703 | 0 | NA | NA | NA | NA |  |
| Female | BRI | 4 | OR | 1.51 (1.25, 1.82) | 0.001 | 98.20% | 0.285 | Zhang et al.,2018 | 1.39(1.26, 1.53) | 89.50% |  |
|  | BMI | 4 | OR | 1.52 (1.16, 1.99) | 0.001 | 99.20% | 0.087 | Su et al.,2023 | 1.66(1.40, 1.98) | 94.90% |  |
|  | WC | 3 | OR | 1.70 (1.44, 2.00) | 0.001 | 93.60% | 0.773 | Zhang et al.,2018 | 1.56(1.48, 1.64) | 0 |  |
|  | VAI | 2 | OR | 1.34 (1.32, 1.36) | 0.987 | 0 | NA | NA | NA | NA |  |
|  | AVI | 2 | OR | 1.35 (1.00, 1.81) | 0.001 | 97.40% | NA | NA | NA | NA |  |
|  | ABSI | 3 | OR | 1.10 (1.00, 1.20) | 0.001 | 93.60% | 0.207 | Zhang et al.,2018 | 1.05(0.99, 1.12) | 86.60% |  |
|  | WHtR | 3 | OR | 1.40 (0.99, 1.98) | 0.001 | 99.80% | 0.299 | Su et al.,2023 | 1.60(1.38, 1.85) | 94.60% |  |
|  | CI | 2 | OR | 1.44 (1.24, 1.67) | 0.001 | 94.00% | NA | NA | NA | NA |  |
| Total | BRI | 6 | AUC | 0.63 (0.60, 0.68) | 0.001 | 99.50% | 0.152 | Su et al.,2023 | 0.62(0.59, 0.65) | 98.10% |  |
|  | BMI | 5 | AUC | 0.65 (0.61, 0.69) | 0.001 | 99.30% | 0.075 | Su et al.,2023 | 0.64(0.62, 0.66) | 94.30% |  |
|  | WHtR | 4 | AUC | 0.64 (0.60, 0.68) | 0.001 | 99.10% | 0.371 | Su et al.,2023 | 0.62(0.62, 0.63) | 0 |  |
|  | VAI | 3 | AUC | 0.65 (0.61, 0.69) | 0.001 | 99.20% | 0.036 | Li et al.,2024b | 0.67(0.64, 0.70) | 98.30% |  |
|  | WHR | 2 | AUC | 0.63 (0.54, 0.74) | 0.001 | 94.90% | NA | NA | NA | NA |  |
|  | AVI | 2 | AUC | 0.67 (0.58, 0.76) | 0.001 | 94.50% | NA | NA | NA | NA |  |
|  | ABSI | 5 | AUC | 0.54 (0.52, 0.56) | 0.001 | 95.40% | 0.664 | Chen et al.,2024 | 0.53(0.52, 0.55) | 73.90% |  |
|  | CMI | 2 | AUC | 0.64 (0.62, 0.65) | 0.053 | 73.20% | NA | NA | NA | NA |  |
|  | WWI | 4 | AUC | 0.56 (0.52, 0.61) | 0.001 | 98.90% | 0.643 | Mao et al.,2024 | 0.55(0.52, 0.58) | 94.50% |  |
|  | LAP | 3 | AUC | 0.68 (0.63, 0.73) | 0.001 | 99.50% | 0.035 | Li et al.,2024b | 0.70(0.66, 0.75) | 99.20% |  |
|  | TyG | 2 | AUC | 0.63 (0.54, 0.73) | 0.001 | 99.20% | NA | NA | NA | NA |  |
|  | CI | 2 | AUC | 0.61 (0.61, 0.62) | 0.961 | 0 | NA | NA | NA | NA |  |
|  | BAI | 2 | AUC | 0.54 (0.54, 0.55) | 0.578 | 0 | NA | NA | NA | NA |  |
| Male | BRI | 10 | AUC | 0.61 (0.60, 0.63) | 0.001 | 95.10% | 0.237 | Kahaer et al.,2022 | 0.62(0.61, 0.64) | 94.00% |  |
|  | VAI | 5 | AUC | 0.64 (0.62, 0.65) | 0.001 | 87.40% | 0.487 | Kahaer et al.,2022 | 0.65(0.63, 0.66) | 81.60% |  |
|  | WC | 8 | AUC | 0.62 (0.60, 0.64) | 0.001 | 93.30% | 0.413 | Kahaer et al.,2022 | 0.64(0.62, 0.65) | 87.20% |  |
|  | WHtR | 9 | AUC | 0.61 (0.60, 0.63) | 0.001 | 93.70% | 0.076 | Kahaer et al.,2022 | 0.62(0.61, 0.63) | 90.90% |  |
|  | ABSI | 7 | AUC | 0.53 (0.52, 0.55) | 0.001 | 92.20% | 0.330 | Zhang et al.,2016 | 0.52(0.51, 0.53) | 79.40% |  |
|  | CMI | 3 | AUC | 0.63 (0.57, 0.69) | 0.001 | 93.30% | 0.914 | Liu et al.,2024b | 0.60(0.55, 0.65) | 88.50% |  |
|  | LAP | 6 | AUC | 0.66 (0.64, 0.67) | 0.001 | 93.60% | 0.494 | Kahaer et al.,2022 | 0.67(0.65, 0.68) | 90.80% |  |
|  | BMI | 10 | AUC | 0.63 (0.61, 0.64) | 0.001 | 94.60% | 0.118 | Kahaer et al.,2022 | 0.64(0.62, 0.65) | 92.30% |  |
|  | CI | 3 | AUC | 0.58 (0.57, 0.59) | 0.001 | 86.40% | 0.564 | Zhang et al.,2018 | 0.57(0.57, 0.58) | 0 |  |
|  | TyG | 3 | AUC | 0.63 (0.60, 0.66) | 0.001 | 93.00% | 0.604 | Kahaer et al.,2022 | 0.65(0.62, 0.67) | 93.90% |  |
| Female | BRI | 10 | AUC | 0.68 (0.66, 0.70) | 0.001 | 96.80% | 0.003 | Li et al.,2025b | 0.69(0.67, 0.70) | 95.10% |  |
|  | VAI | 5 | AUC | 0.70 (0.68, 0.73) | 0.001 | 86.90% | 0.305 | Liu et al.,2024b | 0.71(0.70, 0.73) | 94.10% |  |
|  | WC | 8 | AUC | 0.67 (0.65, 0.70) | 0.001 | 95.00% | 0.03 | Zhang et al.,2018 | 0.67(0.65, 0.69) | 84.00% |  |
|  | WHtR | 9 | AUC | 0.67 (0.65, 0.70) | 0.001 | 97.40% | 0.466 | Robust | NA | NA |  |
|  | ABSI | 7 | AUC | 0.59 (0.56, 0.62) | 0.001 | 98.20% | 0.808 | Su et al.,2023 | 0.60(0.58, 0.63) | 93.20% |  |
|  | CMI | 3 | AUC | 0.69 (0.65, 0.72) | 0.005 | 81.40% | 0.023 | Kahaer et al.,2022 | 0.67(0.65, 0.68) | 37.30% |  |
|  | LAP | 6 | AUC | 0.73 (0.72, 0.75) | 0.001 | 87.00% | 0.051 | Liu et al.,2024a | 0.74(0.73, 0.76) | 84.10% |  |
|  | BMI | 10 | AUC | 0.67 (0.64, 0.69) | 0.001 | 97.60% | 0.074 | Kahaer et al.,2022 | 0.67(0.65, 0.70) | 97.70% |  |
|  | CI | 3 | AUC | 0.64 (0.60, 0.68) | 0.001 | 96.30% | 0.975 | Zhang et al.,2018 | 0.63(0.62, 0.63) | 0 |  |
|  | TyG | 3 | AUC | 0.73 (0.70, 0.76) | 0.001 | 86.20% | 0.662 | Chen et al.,2024 | 0.75(0.74, 0.75) | 0 |  |
| **Non-alcoholic Fatty Liver Disease** | | | | | | | | | | |  |
| Male | BRI | 4 | OR | 3.00 (2.16, 4.18) | 0.001 | 98.80% | 0.929 | Zhao et al.,2024; Motamed et al.,2016 | 3.16(2.90, 3.44) | 80.80% |  |
|  | BMI | 3 | OR | 3.83 (3.06, 4.78) | 0.001 | 95.10% | 0.347 | Motamed et al.,2016 | 3.20(3.09, 3.32) | 11.10% |  |
|  | WC | 3 | OR | 3.81 (2.99, 4.84) | 0.001 | 86.20% | 0.961 | Motamed et al.,2016 | 3.47(3.16, 3.82) | 3.50% |  |
|  | WHR | 2 | OR | 3.32 (2.98, 3.72) | 0.138 | 54.70% | NA | NA | NA | NA |  |
|  | WHtR | 2 | OR | 4.03 (2.38, 6.82) | 0.001 | 97.20% | NA | NA | NA | NA |  |
|  | ABSI | 3 | OR | 1.27 (1.25, 1.30) | 0.484 | 0 | 0.282 | Robust | NA | NA |  |
| Female | BRI | 4 | OR | 2.54 (1.53, 4.22) | 0.001 | 99.40% | 0.845 | Zhao et al.,2024 | 3.11(2.59, 3.72) | 89.90% |  |
|  | BMI | 3 | OR | 3.49 (3.14, 3.87) | 0.042 | 68.40% | 0.503 | Robust | NA | NA |  |
|  | WC | 3 | OR | 3.60 (3.30, 3.93) | 0.155 | 46.40% | 0.821 | Robust | NA | NA |  |
|  | WHR | 2 | OR | 2.16 (1.25, 3.73) | 0.001 | 97.30% | NA | NA | NA | NA |  |
|  | WHtR | 2 | OR | 3.63 (3.46, 3.81) | 0.465 | 0 | NA | NA | NA | NA |  |
|  | ABSI | 3 | OR | 1.14 (1.01, 1.29) | 0.001 | 87.30% | 0.411 | Motamed et al.,2016 | 1.23(1.19, 1.28) | 0 |  |
| Male | BRI | 2 | AUC | 0.78 (0.72, 0.85) | 0.001 | 92.50% | NA | NA | NA | NA |  |
|  | BMI | 2 | AUC | 0.79 (0.73, 0.85) | 0.001 | 91.80% | NA | NA | NA | NA |  |
|  | WHtR | 2 | AUC | 0.78 (0.72, 0.85) | 0.001 | 92.50% | NA | NA | NA | NA |  |
|  | ABSI | 2 | AUC | 0.56 (0.51, 0.61) | 0.030 | 78.90% | NA | NA | NA | NA |  |
|  | VAI | 2 | AUC | 0.69 (0.57, 0.83) | 0.001 | 96.70% | NA | NA | NA | NA |  |
| Female | BRI | 2 | AUC | 0.79 (0.63, 0.98) | 0.001 | 99.50% | NA | NA | NA | NA |  |
|  | BMI | 2 | AUC | 0.79 (0.63, 0.98) | 0.001 | 99.50% | NA | NA | NA | NA |  |
|  | WHtR | 2 | AUC | 0.79 (0.63, 0.98) | 0.001 | 99.50% | NA | NA | NA | NA |  |
|  | ABSI | 2 | AUC | 0.56 (0.46, 0.68) | 0.001 | 97.80% | NA | NA | NA | NA |  |
|  | VAI | 2 | AUC | 0.72 (0.54, 0.95) | 0.001 | 99.50% | NA | NA | NA | NA |  |
| **Metabolic Dysfunction-associated Fatty Liver Disease** | | | | | | | | | | |  |
| Total | BRI | 3 | AUC | 0.79 (0.77, 0.81) | 0.024 | 73.10% | 0.394 | Wang et al.,2023 | 0.80(0.79, 0.81) | 0 |  |
|  | BMI | 3 | AUC | 0.82 (0.75, 0.90) | 0.001 | 99.20% | 0.126 | Wang et al.,2023 | 0.79(0.78, 0.80) | 0 |  |
|  | HSI | 2 | AUC | 0.80 (0.78, 0.82) | 0.244 | 26.40% | NA | NA | NA | NA |  |
|  | LAP | 2 | AUC | 0.84 (0.79, 0.90) | 0.001 | 98.30% | NA | NA | NA | NA |  |
|  | WHR | 2 | AUC | 0.71 (0.64, 0.79) | 0.002 | 89.70% | NA | NA | NA | NA |  |
|  | WHtR | 3 | AUC | 0.82 (0.78, 0.86) | 0.001 | 95.90% | 0.449 | Wang et al.,2023 | 0.80(0.79, 0.81) | 0 |  |
|  | CI | 2 | AUC | 0.72 (0.66, 0.79) | 0.010 | 85.10% | NA | NA | NA | NA |  |
|  | ABSI | 2 | AUC | 0.54 (0.46, 0.63) | 0.001 | 92.60% | NA | NA | NA | NA |  |
|  | AVI | 2 | AUC | 0.81 (0.80, 0.82) | 0.795 | 0 | NA | NA | NA | NA |  |
|  | TyG | 2 | AUC | 0.71 (0.65, 0.78) | 0.006 | 86.80% | NA | NA | NA | NA |  |
| Male | BRI | 2 | AUC | 0.78 (0.69, 0.87) | 0.001 | 98.80% | NA | NA | NA | NA |  |
|  | BMI | 2 | AUC | 0.82 (0.79, 0.86) | 0.001 | 90.20% | NA | NA | NA | NA |  |
|  | WHtR | 2 | AUC | 0.80 (0.76, 0.85) | 0.001 | 94.80% | NA | NA | NA | NA |  |
| Female | BRI | 2 | AUC | 0.82 (0.79, 0.84) | 0.008 | 85.70% | NA | NA | NA | NA |  |
|  | BMI | 2 | AUC | 0.84 (0.75, 0.94) | 0.001 | 98.80% | NA | NA | NA | NA |  |
|  | WHtR | 2 | AUC | 0.83 (0.78, 0.89) | 0.001 | 96.60% | NA | NA | NA | NA |  |
| **Hypertension** | | | | | | | | | | |  |
| Total | BRI | 8 | OR | 1.44 (1.32, 1.57) | 0.001 | 97.00% | 0.208 | Zhan et al.,2024 | 1.50(1.40, 1.61) | 94.70% |  |
|  | BMI | 7 | OR | 1.48 (1.38, 1.60) | 0.001 | 96.10% | 0.194 | Robust | NA | NA |  |
|  | WC | 6 | OR | 1.42 (1.18, 1.70) | 0.001 | 99.60% | 0.703 | Oliveira et al.,2022 | 1.52(1.40, 1.64) | 96.60% |  |
|  | WHtR | 6 | OR | 1.49 (1.39, 1.60) | 0.001 | 94.90% | 0.118 | Zhang et al.,2024b | 1.54(1.44, 1.64) | 91.90% |  |
|  | ABSI | 5 | OR | 1.24 (1.09, 1.42) | 0.001 | 96.30% | 0.346 | Wu et al.,2022b | 1.07(1.05, 1.08) | 0 |  |
|  | VAI | 2 | OR | 1.16 (1.12, 1.20) | 0.737 | 0 | NA | NA | NA | NA |  |
|  | BAI | 3 | OR | 1.12 (0.93, 1.34) | 0.001 | 96.50% | 0.217 | Mansoori et al.,2024 | 1.20(1.06, 1.37) | 24.70% |  |
|  | CI | 3 | OR | 1.31 (1.14, 1.51) | 0.001 | 99.00% | 0.751 | Wu et al.,2022b | 1.23(1.14, 1.32) | 87.30% |  |
|  | LAP | 2 | OR | 1.35 (1.26, 1.44) | 0.037 | 77.10% | NA | NA | NA | NA |  |
| Male | BRI | 5 | OR | 1.55 (1.31, 1.84) | 0.001 | 96.70% | 0.553 | Robust | NA | NA |  |
|  | BMI | 5 | OR | 1.60 (1.20, 2.15) | 0.001 | 99.30% | 0.808 | Hosseini et al.,2024 | 1.76(1.46, 2.11) | 96.40% |  |
|  | WC | 3 | OR | 1.83 (1.78, 1.89) | 0.633 | 0 | 0.799 | Robust | NA | NA |  |
|  | LAP | 2 | OR | 1.39 (1.18, 1.64) | 0.942 | 0 | NA | NA | NA | NA |  |
|  | WHtR | 3 | OR | 1.69 (1.32, 2.18) | 0.001 | 97.20% | 0.894 | Lee et al.,2022b | 1.86(1.81, 1.92) | 0 |  |
|  | ABSI | 3 | OR | 1.14 (1.04, 1.25) | 0.008 | 79.40% | 0.203 | Lee et al.,2022a | 1.08(1.05, 1.11) | 0 |  |
|  | VAI | 3 | OR | 1.13 (1.01, 1.27) | 0.001 | 89.10% | 0.010 | Hosseini et al.,2024 | 1.19(1.11, 1.28) | 43.00% |  |
|  | CI | 5 | OR | 1.25 (1.10, 1.42) | 0.001 | 99.30% | 0.224 | Zhang et al.,2018 | 1.15(1.06, 1.24) | 96.80% |  |
|  | WHR | 3 | OR | 1.31 (1.07, 1.62) | 0.001 | 96.00% | 0.119 | Lee et al.,2022a | 1.12(0.96, 1.32) | 94.70% |  |
|  | BAI | 2 | OR | 1.15 (0.95, 1.39) | 0.001 | 96.60% | NA | NA | NA | NA |  |
|  | AVI | 3 | OR | 1.34 (1.07, 1.68) | 0.001 | 96.60% | 0.152 | Lee et al.,2022a | 1.17(0.96, 1.43) | 96.60% |  |
| Female | BRI | 5 | OR | 1.51 (1.35, 1.68) | 0.001 | 92.00% | 0.721 | Zhang et al.,2018 | 1.46(1.31,1.64) | 87.70% |  |
|  | BMI | 5 | OR | 1.57 (1.22, 2.03) | 0.001 | 99.20% | 0.194 | Hosseini et al.,2024 | 1.71(1.46,1.99) | 94.70% |  |
|  | WC | 3 | OR | 1.53 (1.10, 2.13) | 0.001 | 99.40% | 0.11 | Hosseini et al.,2024 | 1.69(1.63,1.77) | 0 |  |
|  | LAP | 2 | OR | 1.18 (0.99, 1.40) | 0.001 | 91.60% | NA | NA | NA | NA |  |
|  | WHtR | 3 | OR | 1.61 (1.32, 1.96) | 0.001 | 95.20% | 0.917 | Lee et al.,2022b | 1.73(1.65, 1.81) | 0 |  |
|  | ABSI | 2 | OR | 1.16 (0.92, 1.46) | 0.137 | 54.80% | NA | NA | NA | NA |  |
|  | VAI | 3 | OR | 1.18 (1.12, 1.24) | 0.023 | 73.60% | 0.468 | Robust | Robust | Robust |  |
|  | CI | 5 | OR | 1.15 (1.07, 1.22) | 0.001 | 97.40% | 0.016 | Lee et al.,2022b; Hosseini et al.,2024 | 1.30 (1.22, 1.39) | 40.20% |  |
|  | WHR | 3 | OR | 1.21 (1.02, 1.42) | 0.001 | 95.40% | 0.199 | Lee et al.,2022a | 1.10(0.94, 1.28) | 96.60% |  |
|  | BAI | 2 | OR | 1.20 (1.01, 1.44) | 0.001 | 97.50% | NA | NA | NA | NA |  |
|  | AVI | 3 | OR | 1.31 (1.14, 1.51) | 0.001 | 93.40% | 0.217 | Lee et al.,2022a | 1.21(1.07, 1.36) | 93.80% |  |
| Total | BRI | 8 | AUC | 0.64 (0.61, 0.67) | 0.001 | 98.60% | 0.276 | Wu et al.,2022b | 0.63(0.60, 0.66) | 96.60% |  |
|  | BMI | 6 | AUC | 0.63 (0.60, 0.66) | 0.001 | 94.50% | 0.534 | Wu et al.,2022b | 0.62(0.60, 0.63) | 56.90% |  |
|  | WC | 5 | AUC | 0.65 (0.60, 0.69) | 0.001 | 97.10% | 0.579 | Wu et al.,2022b | 0.63(0.60, 0.66) | 80.60% |  |
|  | CI | 4 | AUC | 0.60 (0.51, 0.70) | 0.001 | 99.80% | 0.212 | Wu et al.,2022b | 0.57(0.54, 0.60) | 96.40% |  |
|  | ABSI | 7 | AUC | 0.57 (0.53, 0.62) | 0.001 | 99.10% | 0.102 | Robust | NA | NA |  |
|  | WHR | 4 | AUC | 0.61 (0.58, 0.63) | 0.001 | 84.90% | 0.512 | Robust | NA | NA |  |
|  | BAI | 3 | AUC | 0.58 (0.55, 0.61) | 0.001 | 93.00% | 0.644 | Feng et al.,2024a | 0.59(0.57, 0.61) | 58.80% |  |
|  | LAP | 3 | AUC | 0.61 (0.59, 0.64) | 0.001 | 95.80% | 0.554 | Li et al.,2024b | 0.63(0.62, 0.63) | 0 |  |
|  | WHtR | 6 | AUC | 0.63 (0.61, 0.65) | 0.001 | 92.20% | 0.222 | Wu et al.,2022b | 0.63(0.61, 0.65) | 81.70% |  |
| Male | BRI | 9 | AUC | 0.65 (0.63, 0.68) | 0.001 | 96.20% | 0.595 | Zhang et al.,2018 | 0.64(0.62, 0.66) | 89.30% |  |
|  | BMI | 9 | AUC | 0.63 (0.61, 0.65) | 0.001 | 95.50% | 0.318 | Zhang et al.,2018 | 0.62(0.60, 0.64) | 83.70% |  |
|  | WC | 7 | AUC | 0.64 (0.61, 0.68) | 0.001 | 98.00% | 0.332 | Zhang et al.,2018 | 0.63(0.61, 0.66) | 92.50% |  |
|  | VAI | 3 | AUC | 0.58 (0.56, 0.59) | 0.147 | 47.80% | 0.052 | Robust | NA | NA |  |
|  | AVI | 4 | AUC | 0.64 (0.59, 0.69) | 0.001 | 92.40% | 0.339 | Lee et al.,2022a | 0.60(0.59, 0.62) | 39.90% |  |
|  | WHtR | 9 | AUC | 0.65 (0.63, 0.68) | 0.001 | 96.20% | 0.459 | Zhang et al.,2018 | 0.64(0.63, 0.66) | 84.50% |  |
|  | ABSI | 8 | AUC | 0.58 (0.56, 0.61) | 0.001 | 95.40% | 0.654 | Zhang et al.,2018 | 0.58(0.55, 0.62) | 84.50% |  |
|  | LAP | 3 | AUC | 0.61 (0.59, 0.64) | 0.01 | 78.20% | 0.352 | Robust | NA | NA |  |
|  | CI | 6 | AUC | 0.63 (0.60, 0.66) | 0.001 | 92.20% | 0.927 | Lee et al.,2022a | 0.61(0.59, 0.64) | 90.10% |  |
|  | WHR | 5 | AUC | 0.64 (0.60, 0.69) | 0.001 | 93.80% | 0.667 | Lee et al.,2022a | 0.62(0.60, 0.64) | 71.00% |  |
| Female | BRI | 9 | AUC | 0.69 (0.65, 0.73) | 0.001 | 97.90% | 0.395 | Zhang et al.,2018 | 0.68(0.66, 0.70) | 91.20% |  |
|  | BMI | 9 | AUC | 0.66 (0.62, 0.69) | 0.001 | 97.40% | 0.456 | Zhang et al.,2018 | 0.65(0.62, 0.67) | 90.10% |  |
|  | WC | 7 | AUC | 0.68 (0.64, 0.72) | 0.001 | 97.70% | 0.458 | Zhang et al.,2019 | 0.67(0.64, 0.70) | 91.70% |  |
|  | VAI | 3 | AUC | 0.60 (0.57, 0.63) | 0.001 | 86.60% | 0.120 | Li et al.,2023a | 0.61(0.60, 0.63) | 72.30% |  |
|  | AVI | 4 | AUC | 0.69 (0.62, 0.77) | 0.001 | 93.70% | 0.433 | Lee et al.,2022a | 0.66(0.62, 0.71) | 70.70% |  |
|  | WHtR | 9 | AUC | 0.69 (0.66, 0.73) | 0.001 | 97.70% | 0.348 | Zhang et al.,2018 | 0.68(0.66, 0.70) | 89.90% |  |
|  | ABSI | 8 | AUC | 0.61 (0.58, 0.64) | 0.001 | 94.70% | 0.862 | Zhang et al.,2018 | 0.61(0.58, 0.64) | 91.40% |  |
|  | LAP | 3 | AUC | 0.64 (0.61, 0.66) | 0.001 | 85.40% | 0.004 | Li et al.,2023a | 0.65(0.64, 0.66) | 11.70% |  |
|  | CI | 6 | AUC | 0.66 (0.61, 0.72) | 0.001 | 97.80% | 0.910 | Zhang et al.,2018 | 0.65(0.60, 0.70) | 94.70% |  |
|  | WHR | 5 | AUC | 0.67 (0.63, 0.72) | 0.001 | 93.40% | 0.673 | Lee et al.,2022a | 0.64(0.63, 0.66) | 19.50% |  |
| **Chronic Kidney Disease** | | | | | | | | | | |  |
| Total | BRI | 5 | OR | 1.15 (1.03, 1.28) | 0.001 | 92.20% | 0.607 | Rezaee et al.,2025; Chen et al.,2025 | 1.14(1.09, 1.19) | 3.30% |  |
|  | BMI | 2 | OR | 1.15 (0.97, 1.35) | 0.001 | 94.40% | NA | NA | NA | NA |  |
|  | WC | 2 | OR | 1.15 (0.94, 1.40) | 0.001 | 96.40% | NA | NA | NA | NA |  |
|  | WHtR | 3 | OR | 1.29 (1.16, 1.42) | 0.324 | 11.20% | 0.437 | Robust | NA | NA |  |
|  | ABSI | 3 | OR | 1.05 (0.73, 1.51) | 0.015 | 76.40% | 0.588 | Rezaee et al.,2025 | 1.18(1.10, 1.26) | 3.70% |  |
|  | CI | 2 | OR | 1.29 (1.23, 1.37) | 0.486 | 0 | NA | NA | NA | NA |  |
| Male | BRI | 2 | OR | 1.10 (0.97, 1.25) | 0.006 | 86.60% | NA | NA | NA | NA |  |
| Female | BRI | 2 | OR | 1.03 (1.01, 1.05) | 1.000 | 0 | NA | NA | NA | NA |  |
| Total | BRI | 4 | AUC | 0.62 (0.60, 0.63) | 0.087 | 54.40% | 0.277 | Zhang et al.,2024c | 0.60(0.61, 0.62) | 0 |  |
|  | BMI | 3 | AUC | 0.55 (0.54, 0.56) | 0.316 | 13.20% | 0.296 | Robust | NA | NA |  |
|  | WC | 3 | AUC | 0.60 (0.59, 0.60) | 0.462 | 0.00% | 0.112 | Robust | NA | NA |  |
|  | WHtR | 3 | AUC | 0.62 (0.61, 0.63) | 0.571 | 0 | 0.228 | Robust | NA | NA |  |
|  | ABSI | 4 | AUC | 0.64 (0.60, 0.67) | 0.001 | 94.60% | 0.919 | Zhang et al.,2024c | 0.65(0.60, 0.69) | 76.00% |  |
|  | CI | 2 | AUC | 0.66 (0.66, 0.67) | 0.956 | 0 | NA | NA | NA | NA |  |
|  | VAI | 2 | AUC | 0.57 (0.53, 0.61) | 0.846 | 0 | NA | NA | NA | NA |  |
|  | LAP | 2 | AUC | 0.59 (0.55, 0.64) | 0.18 | 44.40% | NA | NA | NA | NA |  |
| **Cardiovascular Disease** | | | | | | | | | | |  |
| Total | BRI | 8 | OR | 1.16 (1.08, 1.26) | 0.001 | 93.20% | 0.001 | Li et al.,2025a | 1.19(1.11,1.28) | 77.20% |  |
|  | BMI | 4 | OR | 1.11 (0.99, 1.23) | 0.002 | 79.80% | 0.285 | Robust | NA | NA |  |
|  | WC | 3 | OR | 1.13 (0.97, 1.31) | 0.001 | 92.70% | 0.377 | Hu et al.,2025 | 1.03(0.98, 1.08) | 33.80% |  |
|  | CVAI | 2 | OR | 1.22 (1.07, 1.38) | 0.001 | 69.60% | NA | NA | NA | NA |  |
| Male | BRI | 4 | OR | 1.23 (1.02, 1.48) | 0.001 | 96.50% | 0.92 | Wu et al.,2022a | 1.13(1.03,1.23) | 74.80% |  |
| Female | BRI | 5 | OR | 1.14 (1.08, 1.20) | 0.01 | 70.10% | 0.648 | Wu et al.,2022a | 1.10(1.07,1.14) | 0 |  |
| **Heart disease** | | | | | | | | | | |  |
| Total | BRI | 3 | OR | 1.08 (0.99, 1.19) | 0.024 | 73.10% | 0.294 | Zhang et al.,2023 | 1.13(1.03, 1.25) | 41.10% |  |
|  | BMI | 2 | OR | 1.13 (1.05, 1.22) | 0.335 | 0 | NA | NA | NA | NA |  |
|  | WC | 2 | OR | 1.16 (1.03, 1.30) | 0.120 | 58.70% | NA | NA | NA | NA |  |
| **Coronary heart disease** | | | | | | | | | | |  |
| Total | BRI | 3 | RR | 1.13 (1.05, 1.22) | 0.252 | 27.50% | 0.822 | Robust | NA | NA |  |
|  | BMI | 2 | RR | 1.12 (1.08, 1.17) | 0.735 | 0 | NA | NA | NA | NA |  |
|  | WC | 2 | RR | 1.16 (1.11, 1.21) | 0.662 | 0 | NA | NA | NA | NA |  |
|  | WHtR | 2 | RR | 1.06 (0.86, 1.31) | 0.069 | 69.70% | NA | NA | NA | NA |  |
|  | ABSI | 2 | RR | 1.09 (1.03, 1.14) | 0.359 | 0 | NA | NA | NA | NA |  |
| **Stroke** | | | | | | | | | | |  |
| Total | BRI | 9 | OR | 1.14 (1.10, 1.19) | 0.012 | 59.30% | 0.771 | Robust | NA | NA |  |
|  | BMI | 3 | OR | 1.13 (1.04, 1.23) | 0.035 | 70.10% | 0.261 | Robust | NA | NA |  |
|  | WC | 3 | OR | 1.14 (1.06, 1.23) | 0.064 | 63.50% | 0.484 | Hu et al.,2025 | 1.12(1.07, 1.16) | 6.40% |  |
|  | WHtR | 3 | OR | 1.12 (0.96, 1.31) | 0.001 | 91.70% | 0.738 | Qin et al.,2023 | 1.13(0.79, 1.61) | 94.30% |  |
|  | ABSI | 2 | OR | 1.11 (1.07, 1.16) | 0.835 | 0 | NA | NA | NA | NA |  |
| Male | BRI | 3 | HR | 1.07 (0.95, 1.20) | 0.045 | 67.70% | NA | NA | NA | NA |  |
| Female | BRI | 3 | HR | 1.10 (0.99, 1.21) | 0.040 | 69.00% | NA | NA | NA | NA |  |
| **All-cause Mortality** | | | | | | | | | | |  |
| Total | BRI | 6 | HR | 1.00 (0.95, 1.05) | 0.001 | 88.20% | 0.149 | Liu et al.,2023 | 1.01(0.97, 1.06) | 85.10% |  |
| Non-Hispanic  White | BRI | 2 | HR | 0.95 (0.84, 1.08) | 0.006 | 86.60% | NA | NA | NA | NA |  |
| Non-Hispanic  Black | BRI | 2 | HR | 0.95 (0.81, 1.11) | 0.013 | 83.60% | NA | NA | NA | NA |  |
| Mexican  American | BRI | 2 | HR | 1.00 (0.93, 1.07) | 0.448 | 0 | NA | NA | NA | NA |  |
| Male | BRI | 3 | HR | 1.07 (1.01, 1.12) | 0.301 | 16.70% | 0.852 | Robust | NA | NA |  |
| Female | BRI | 3 | HR | 1.00 (0.87, 1.15) | 0.001 | 84.90% | 0.837 | Liu et al.,2025 | 0.93(0.81, 1.07) | 83.50% |  |
| HTN | BRI | 3 | HR | 1.02 (0.90, 1.16) | 0.001 | 88.40% | 0.968 | Ding et al.,2025 | 1.09(0.97, 1.22) | 76.20% |  |
| Non-HTN | BRI | 3 | HR | 0.97 (0.91, 1.03) | 0.265 | 24.70% | 0.695 | Robust | NA | NA |  |
| **Cardiovascular Disease Mortality** | | | | | | | | | | |  |
| Total | BRI | 4 | HR | 1.11 (1.05, 1.17) | 0.012 | 72.50% | 0.037 | Liu et al.,2025 | 1.08(1.04, 1.12) | 41.40% |  |
| Abbreviations: No.study, Number of Study; CKM, Cardiovascular-Kidney-Metabolic; Excl., Exclusion of influential study(ies) identified by leave-one-out sensitivity analysis; Robust: No significant change in effect size or I^2^ after excluding any study; NA, Not applicable (insufficient studies to perform leave-one-out analysis); OR, odds ratio; RR, relative risk; HR, hazard ratios; AUC, Area Under the Curve; 95% CI, 95% confidence interval; BMI, body mass index; WC, waist circumference; WHtR, waist-to-height ratio; BRI, body roundness index; CI, conicity index; LAP, lipid accumulation product index; VAI, visceral adiposity index; CVAI, Chinese visceral adiposity index; CUN-BAE, Clínica Universidad de Navarra-Body Adiposity Estimator; ABSI, A body shape index; BF, Body Fat Ratio; WHR, Waist-to-Hip Ratio; BAI, Body Adiposity Index; HC, hip circumference; CMI, cardiometabolic index; WWI, weight-adjusted-waist; AVI, Abdominal Volume Index; TyG, Triglyceride-glucose; HSI, hepatic steatosis index; TG/HDL-C, triglyceride to high-density lipoprotein cholesterol ratio; NC, Neck Circumference; AIP, atherogenic index of plasma; WHHR, waist-hip-height ratio; | | | | | | | | | | |  |
|  |  |  |  |  |  |  |  |  |  |  |  |
|  |  |  |  |  |  |  |  |  |  |  |  |
|  |  |  |  |  |  |  |  |  |  |  |  |
|  |  |  |  |  |  |  |  |  |  |  |  |
|  |  |  |  |  |  |  |  |  |  |  |  |
|  | | | | | | | | | | | |
|  |  |  |  |  |  |  |  |  |  |  |  |

| Suppl. Table 5 The subgroup analysis by study design. | | | | | | | | | | |
| --- | --- | --- | --- | --- | --- | --- | --- | --- | --- | --- |
| Subgroup | Indicator | No.study | Outcome assessment | Effect (95% CI) | Test of heterogeneity | | Egger's test | Sensitivity Analysis | Effect (Excl.)  (95% CI) | I^2^ (Excl.) |
|  |  |  |  |  | P value | I^2^ |  |  |  |  |
| **Cardiovascular Disease** | | | | | | | | | | |
| Cross-sectional Study | BRI | 5 | OR | 1.19(1.03, 1.37) | 0.001 | 93.90% | 0.017 | Li et al., 2025a | 1.23 (1.13, 1.35) | 57.00% |
| Cohort Study | BRI | 3 | HR | 1.15(1.05, 1.26) | 0.023 | 73.50% | 0.115 | Robust | NA | NA |
| **Type 2 diabetes** | | | | | | | | | | |
| Cross-sectional Study | BRI | 3 | OR | 1.47 (1.10, 1.96) | 0.001 | 87.60% | 0.045 | Robust | NA | NA |
| Cohort Study | BRI | 3 | HR | 1.47 (1.19, 1.81) | 0.006 | 80.20% | 0.277 | Robust | NA | NA |
| **Diabetes Mellitus** | | | | | | | | | | |
| Cross-sectional Study | BRI | 2 | OR | 1.44(1.42, 1.46) | 0.741 | 0 | NA | NA | NA | NA |
| Cohort Study | BRI | 4 | HR | 1.26(1.11, 1.42) | 0.001 | 94.00% | 0.142 | Robust | NA | NA |
| Abbreviations: No.study, Number of Study; Excl., Exclusion of influential study(ies) identified by leave-one-out sensitivity analysis; Robust: No significant change in effect size or I2 after excluding any study; NA, Not applicable (insufficient studies to perform leave-one-out analysis); OR, odds ratio;HR, hazard ratios; 95% CI, 95% confidence interval;BRI, body roundness index; | | | | | | | | | | |
|  |  |  |  |  |  |  |  |  |  |  |

| Suppl. Table 6 Quality assessment of studies included in the review | | | | | | | | | | | | | | | | | | | | | | | | | | | | | | | | | |
| --- | --- | --- | --- | --- | --- | --- | --- | --- | --- | --- | --- | --- | --- | --- | --- | --- | --- | --- | --- | --- | --- | --- | --- | --- | --- | --- | --- | --- | --- | --- | --- | --- | --- |
| Study ID criteria | 1 | 2 | 3 | 4 | 5 | 6 | 7 | 8 | 9 | 10 | 11 | 12 | 13 | 14 | 15 | 16 | 17 | 18 | 19 | 20 | 21 | 22 | 23 | 24 | 25 | 26 | 27 | 28 | 29 | 30 | 31 | 32 | 33 |
| 1. Was the research question or objective in this paper clearly stated? | Y | Y | Y | Y | Y | Y | Y | Y | Y | Y | Y | Y | Y | Y | Y | Y | Y | Y | Y | Y | Y | Y | Y | Y | Y | Y | Y | Y | Y | Y | Y | Y | Y |
| 2. Was the study population clearly specified and defined? | Y | Y | Y | Y | Y | Y | Y | Y | Y | Y | Y | Y | Y | Y | Y | Y | Y | Y | Y | Y | Y | Y | Y | Y | Y | Y | Y | Y | Y | Y | Y | Y | Y |
| 3. Was the participation rate of eligible persons at least 50%? | Y | Y | Y | Y | Y | Y | Y | Y | Y | Y | Y | Y | Y | Y | Y | Y | Y | Y | Y | Y | Y | Y | Y | Y | Y | Y | Y | Y | Y | Y | Y | Y | Y |
| 4. Were the all the subjects selected or recruited from the same or similar population (including the same time period? Were inclusion and exclusion criteria for being in the study pre-specified and applied uniformly to all participants? | Y | Y | Y | Y | Y | Y | Y | Y | Y | Y | Y | Y | Y | Y | Y | Y | Y | Y | Y | Y | Y | Y | Y | Y | Y | Y | Y | Y | Y | Y | Y | Y | Y |
| 5. Was a simple size justification, power description or variance and effect estimates provided? | N | N | N | Y | N | N | N | Y | N | N | N | N | N | N | N | N | N | N | N | N | N | N | N | N | N | N | N | N | N | N | N | N | N |
| 6. For the analysis in this paper, were the exposure(s) of interest measured prior to the outcome(s) being measured? | Y | Y | Y | N | Y | N | Y | N | Y | N | N | Y | N | N | N | Y | N | Y | Y | Y | Y | Y | Y | Y | N | N | N | N | N | N | N | N | Y |
| 7. Was the timeframe sufficient so that one could reasonably expect to see an association between exposure and outcome if it existed? | Y | Y | Y | N | Y | N | Y | N | Y | N | N | Y | N | N | N | Y | N | Y | Y | Y | Y | Y | Y | Y | N | N | N | N | N | N | N | N | Y |
| 8. For exposures that can vary in amount or level, did the study examine different levels of the exposure as related to the outcome (e.g categories of exposure or exposure measured as continuous variable)? | Y | Y | Y | Y | Y | Y | Y | Y | Y | Y | Y | Y | Y | Y | Y | Y | Y | Y | Y | Y | Y | Y | Y | Y | Y | Y | Y | Y | Y | Y | Y | Y | N |
| 9. Were the exposure measures (indepent variable) clearly defined, valid, reliable and implemented consistently across all study participant? | Y | Y | Y | Y | Y | Y | Y | Y | Y | Y | Y | Y | Y | Y | Y | Y | Y | Y | Y | Y | Y | Y | Y | Y | Y | Y | Y | Y | Y | Y | Y | Y | Y |
| 10. Was the exposure(s) assessed more than once over time? | Y | N | N | N | N | N | N | N | Y | N | N | N | N | N | N | N | N | N | N | N | N | N | N | N | N | N | N | N | N | N | N | N | N |
| 11. Were the outcome measures (dependent variables) clearly defined, valid reliable and implemented consistently across all study participants? | Y | Y | Y | Y | Y | Y | Y | Y | Y | Y | Y | Y | Y | Y | Y | Y | Y | Y | Y | Y | Y | Y | Y | Y | Y | Y | Y | Y | Y | Y | Y | Y | Y |
| 12. Were the outcome assessors blinded to the exposure status of participants? | NR | NR | NR | NR | NR | NR | NR | NR | NR | NR | NR | NR | NR | NR | NR | NR | NR | NR | NR | NR | NR | NR | NR | NR | NR | NR | NR | NR | NR | NR | NR | NR | Y |
| 13. Was loss to follow-up after baseline 20% or less? | Y | Y | N | NA | Y | NA | Y | NA | Y | NA | NA | Y | NA | NA | NA | Y | NA | Y | Y | Y | Y | Y | Y | N | NA | NA | NA | NA | NA | NA | NA | NA | Y |
| 14. Were key potential confounding variables measured and adjusted statistically for their impact relationship between exposure(s) and outcome(s)? | Y | Y | Y | Y | Y | Y | Y | Y | Y | Y | Y | Y | Y | Y | Y | Y | N | Y | Y | Y | Y | Y | Y | Y | Y | Y | Y | Y | Y | Y | Y | Y | Y |
| Sum of SCOREs | 12 | 11 | 10 | 9 | 11 | 8 | 11 | 9 | 12 | 8 | 8 | 11 | 8 | 8 | 8 | 11 | 7 | 11 | 11 | 11 | 11 | 11 | 11 | 10 | 8 | 8 | 8 | 8 | 8 | 8 | 8 | 8 | 11 |
| Abbreviations: CD: cannot determine; N: no; Y: Yes;NA: not applicable; NR: not reported | | | | | | | | | | | | | | | | | | | | | | | | | | | | | | | | | |

| **Continued Suppl. Table 6** Quality assessment of studies included in the review | | | | | | | | | | | | | | | | | | | | | | | | | | | | | | | | | |
| --- | --- | --- | --- | --- | --- | --- | --- | --- | --- | --- | --- | --- | --- | --- | --- | --- | --- | --- | --- | --- | --- | --- | --- | --- | --- | --- | --- | --- | --- | --- | --- | --- | --- |
| Study ID criteria | 34 | 35 | 36 | 37 | 38 | 39 | 40 | 41 | 42 | 43 | 44 | 45 | 46 | 47 | 48 | 49 | 50 | 51 | 52 | 53 | 54 | 55 | 56 | 57 | 58 | 59 | 60 | 61 | 62 | 63 | 64 | 65 | 66 |
| 1. Was the research question or objective in this paper clearly stated? | Y | Y | Y | Y | Y | Y | Y | Y | Y | Y | Y | Y | Y | Y | Y | Y | Y | Y | Y | Y | Y | Y | Y | Y | Y | Y | Y | Y | Y | Y | Y | Y | Y |
| 2. Was the study population clearly specified and defined? | Y | Y | Y | Y | Y | Y | Y | Y | Y | Y | Y | Y | Y | Y | Y | Y | Y | Y | Y | Y | Y | Y | Y | Y | Y | Y | Y | Y | Y | Y | Y | Y | Y |
| 3. Was the participation rate of eligible persons at least 50%? | Y | Y | Y | Y | Y | Y | Y | Y | Y | Y | Y | Y | Y | Y | Y | Y | Y | Y | Y | Y | Y | Y | Y | Y | Y | Y | Y | Y | Y | Y | Y | Y | Y |
| 4. Were the all the subjects selected or recruited from the same or similar population (including the same time period? Were inclusion and exclusion criteria for being in the study pre-specified and applied uniformly to all participants? | Y | Y | Y | Y | Y | Y | Y | Y | Y | Y | Y | Y | Y | Y | Y | Y | Y | Y | Y | Y | Y | Y | Y | Y | Y | Y | Y | Y | Y | Y | Y | Y | Y |
| 5. Was a simple size justification, power description or variance and effect estimates provided? | N | N | N | N | N | N | N | N | N | N | N | N | N | N | N | N | N | N | N | N | N | N | N | Y | Y | N | N | N | Y | N | N | N | N |
| 6. For the analysis in this paper, were the exposure(s) of interest measured prior to the outcome(s) being measured? | N | Y | N | N | N | N | Y | Y | Y | N | N | Y | N | N | N | Y | N | Y | N | N | Y | N | Y | N | N | N | Y | Y | N | Y | Y | N | N |
| 7. Was the timeframe sufficient so that one could reasonably expect to see an association between exposure and outcome if it existed? | N | Y | N | N | N | N | Y | Y | Y | N | N | Y | N | N | N | Y | N | Y | N | N | Y | N | Y | N | N | N | Y | Y | N | Y | Y | N | N |
| 8. For exposures that can vary in amount or level, did the study examine different levels of the exposure as related to the outcome (e.g categories of exposure or exposure measured as continuous variable)? | Y | Y | Y | Y | Y | Y | Y | Y | Y | Y | Y | Y | Y | Y | Y | Y | Y | Y | Y | Y | Y | Y | Y | Y | Y | Y | Y | Y | Y | Y | Y | Y | Y |
| 9. Were the exposure measures (indepent variable) clearly defined, valid, reliable and implemented consistently across all study participant? | Y | Y | Y | Y | Y | Y | Y | Y | Y | Y | Y | Y | Y | Y | Y | Y | Y | Y | Y | Y | Y | Y | Y | Y | Y | Y | Y | Y | Y | Y | Y | Y | Y |
| 10. Was the exposure(s) assessed more than once over time? | N | N | N | N | N | N | N | N | N | N | N | Y | N | N | N | N | N | N | N | N | N | N | N | N | N | N | N | N | N | N | N | N | N |
| 11. Were the outcome measures (dependent variables) clearly defined, valid reliable and implemented consistently across all study participants? | Y | Y | Y | Y | Y | Y | Y | Y | Y | Y | Y | Y | Y | Y | Y | Y | Y | Y | Y | Y | Y | Y | Y | Y | Y | Y | Y | Y | Y | Y | Y | Y | Y |
| 12. Were the outcome assessors blinded to the exposure status of participants? | NR | NR | NR | NR | NR | NR | NR | NR | NR | NR | NR | NR | NR | NR | NR | NR | NR | NR | NR | NR | NR | NR | NR | NR | NR | NR | NR | NR | NR | NR | NR | NR | NR |
| 13. Was loss to follow-up after baseline 20% or less? | NA | Y | NA | NA | NA | NA | Y | Y | Y | NA | NA | Y | NA | NA | NA | Y | NA | Y | NA | NA | Y | NA | Y | NA | NA | NA | Y | Y | NA | Y | Y | NA | NA |
| 14. Were key potential confounding variables measured and adjusted statistically for their impact relationship between exposure(s) and outcome(s)? | Y | Y | Y | Y | Y | Y | Y | Y | Y | Y | Y | Y | Y | Y | Y | Y | Y | Y | Y | Y | Y | Y | Y | Y | Y | Y | Y | Y | Y | Y | Y | Y | Y |
| Sum of SCOREs | 8 | 11 | 8 | 8 | 8 | 8 | 11 | 11 | 11 | 8 | 8 | 12 | 8 | 8 | 8 | 11 | 8 | 11 | 8 | 8 | 11 | 8 | 11 | 9 | 9 | 8 | 11 | 11 | 9 | 11 | 11 | 8 | 8 |
| Abbreviations: CD: cannot determine; N: no; Y: Yes;NA: not applicable; NR: not reported | | | | | | | | | | | | | | | | | | | | | | | | | | | | | | | | | |

| **Continued Suppl. Table 6** Quality assessment of studies included in the review | | | | | | | | | | | | | | | | | | | | | | | | | | | |
| --- | --- | --- | --- | --- | --- | --- | --- | --- | --- | --- | --- | --- | --- | --- | --- | --- | --- | --- | --- | --- | --- | --- | --- | --- | --- | --- | --- |
| Study ID criteria | 67 | 68 | 69 | 70 | 71 | 72 | 73 | 74 | 75 | 76 | 77 | 78 | 79 | 80 | 81 | 82 | 83 | 84 | 85 | 86 | 87 | 88 | 89 | 90 | 91 | 92 | 93 |
| 1. Was the research question or objective in this paper clearly stated? | Y | Y | Y | Y | Y | Y | Y | Y | Y | Y | Y | Y | Y | Y | Y | Y | Y | Y | Y | Y | Y | Y | Y | Y | Y | Y | Y |
| 2. Was the study population clearly specified and defined? | Y | Y | Y | Y | Y | Y | Y | Y | Y | Y | Y | Y | Y | Y | Y | Y | Y | Y | Y | Y | Y | Y | Y | Y | Y | Y | Y |
| 3. Was the participation rate of eligible persons at least 50%? | Y | Y | Y | Y | Y | Y | Y | Y | Y | Y | Y | Y | Y | Y | Y | Y | Y | Y | Y | Y | Y | Y | Y | Y | Y | Y | Y |
| 4. Were the all the subjects selected or recruited from the same or similar population (including the same time period? Were inclusion and exclusion criteria for being in the study pre-specified and applied uniformly to all participants? | Y | Y | Y | Y | Y | Y | Y | Y | Y | Y | Y | Y | Y | Y | Y | Y | Y | Y | Y | Y | Y | Y | Y | Y | Y | Y | Y |
| 5. Was a simple size justification, power description or variance and effect estimates provided? | N | N | N | Y | N | N | N | N | N | N | N | N | N | Y | N | N | N | N | N | N | N | N | N | N | N | N | N |
| 6. For the analysis in this paper, were the exposure(s) of interest measured prior to the outcome(s) being measured? | N | N | Y | N | Y | N | N | Y | N | N | Y | Y | N | Y | N | Y | N | N | N | N | N | N | Y | Y | N | N | N |
| 7. Was the timeframe sufficient so that one could reasonably expect to see an association between exposure and outcome if it existed? | N | N | Y | N | Y | N | N | Y | N | N | Y | Y | N | Y | N | Y | N | N | N | N | N | N | Y | Y | N | N | N |
| 8. For exposures that can vary in amount or level, did the study examine different levels of the exposure as related to the outcome (e.g categories of exposure or exposure measured as continuous variable)? | Y | Y | Y | Y | Y | Y | Y | Y | Y | Y | Y | Y | Y | Y | Y | Y | Y | Y | Y | Y | Y | Y | Y | Y | Y | Y | Y |
| 9. Were the exposure measures (indepent variable) clearly defined, valid, reliable and implemented consistently across all study participant? | Y | Y | Y | Y | Y | Y | Y | Y | Y | Y | Y | Y | Y | Y | Y | Y | Y | Y | Y | Y | Y | Y | Y | Y | Y | Y | Y |
| 10. Was the exposure(s) assessed more than once over time? | N | N | N | N | N | N | N | N | N | N | N | N | N | Y | N | N | N | N | N | N | N | N | N | N | N | N | N |
| 11. Were the outcome measures (dependent variables) clearly defined, valid reliable and implemented consistently across all study participants? | Y | Y | Y | Y | Y | Y | Y | Y | Y | Y | Y | Y | Y | Y | Y | Y | Y | Y | Y | Y | Y | Y | Y | Y | Y | Y | Y |
| 12. Were the outcome assessors blinded to the exposure status of participants? | NR | NR | NR | NR | NR | NR | NR | NR | NR | NR | NR | NR | NR | NR | NR | NR | NR | NR | NR | NR | NR | NR | NR | NR | NR | NR | NR |
| 13. Was loss to follow-up after baseline 20% or less? | NA | NA | Y | NA | Y | NA | NA | Y | NA | NA | Y | Y | NA | Y | NA | Y | NA | NA | NA | NA | NA | NA | Y | Y | NA | NA | NA |
| 14. Were key potential confounding variables measured and adjusted statistically for their impact relationship between exposure(s) and outcome(s)? | Y | Y | Y | Y | Y | Y | Y | Y | Y | Y | Y | Y | Y | Y | Y | Y | Y | Y | Y | Y | Y | Y | Y | Y | Y | Y | Y |
| Sum of SCOREs | 8 | 8 | 11 | 9 | 11 | 8 | 8 | 11 | 8 | 8 | 11 | 11 | 8 | 13 | 8 | 11 | 8 | 8 | 8 | 8 | 8 | 8 | 11 | 11 | 8 | 8 | 8 |
| Abbreviations: CD: cannot determine; N: no; Y: Yes;NA: not applicable; NR: not reported | | | | | | | | | | | | | | | | | | | | | | | | | | | |

**Guidance for Assessing the Quality of Observational Cohort and Cross-Sectional Studies**

The guidance document below is organized by question number from the tool for quality assessment of observational cohort and cross-sectional studies.

**Question 1. Research question**

Did the authors describe their goal in conducting this research? Is it easy to understand what they were looking to find? This issue is important for any scientific paper of any type. Higher quality scientific research explicitly defines a research question.

**Questions 2 and 3. Study population**

**Did the authors describe the group of people from which the study participants were selected or recruited, using demographics, location, and time period? If you were to conduct this study again, would you know who to recruit, from where, and from what time period? Is the cohort population free of the outcomes of interest at the time they were recruited?**

An example would be men over 40 years old with type 2 diabetes who began seeking medical care at Phoenix Good Samaritan Hospital between January 1, 1990 and December 31, 1994. In this example, the population is clearly described as: (1) who (men over 40 years old with type 2 diabetes); (2) where (Phoenix Good Samaritan Hospital); and (3) when (between January 1, 1990 and December 31, 1994). Another example is women ages 34 to 59 years of age in 1980 who were in the nursing profession and had no known coronary disease, stroke, cancer, hypercholesterolemia, or diabetes, and were recruited from the 11 most populous States, with contact information obtained from State nursing boards.

In cohort studies, it is crucial that the population at baseline is free of the outcome of interest. For example, the nurses' population above would be an appropriate group in which to study incident coronary disease. This information is usually found either in descriptions of population recruitment, definitions of variables, or inclusion/exclusion criteria.

You may need to look at prior papers on methods in order to make the assessment for this question. Those papers are usually in the reference list.

If fewer than 50% of eligible persons participated in the study, then there is concern that the study population does not adequately represent the target population. This increases the risk of bias.

**Question 4. Groups recruited from the same population and uniform eligibility criteria**

Were the inclusion and exclusion criteria developed prior to recruitment or selection of the study population? Were the same underlying criteria used for all of the subjects involved? This issue is related to the description of the study population, above, and you may find the information for both of these questions in the same section of the paper.

Most cohort studies begin with the selection of the cohort; participants in this cohort are then measured or evaluated to determine their exposure status. However, some cohort studies may recruit or select exposed participants in a different time or place than unexposed participants, especially retrospective cohort studieswhich is when data are obtained from the past (retrospectively), but the analysis examines exposures prior to outcomes. For example, one research question could be whether diabetic men with clinical depression are at higher risk for cardiovascular disease than those without clinical depression. So, diabetic men with depression might be selected from a mental health clinic, while diabetic men without depression might be selected from an internal medicine or endocrinology clinic. This study recruits groups from different clinic populations, so this example would get a "no."

However, the women nurses described in the question above were selected based on the same inclusion/exclusion criteria, so that example would get a "yes."

**Question 5. Sample size justification**

Did the authors present their reasons for selecting or recruiting the number of people included or analyzed? Do they note or discuss the statistical power of the study? This question is about whether or not the study had enough participants to detect an association if one truly existed.

A paragraph in the methods section of the article may explain the sample size needed to detect a hypothesized difference in outcomes. You may also find a discussion of power in the discussion section (such as the study had 85 percent power to detect a 20 percent increase in the rate of an outcome of interest, with a 2-sided alpha of 0.05). Sometimes estimates of variance and/or estimates of effect size are given, instead of sample size calculations. In any of these cases, the answer would be "yes."

However, observational cohort studies often do not report anything about power or sample sizes because the analyses are exploratory in nature. In this case, the answer would be "no." This is not a "fatal flaw." It just may indicate that attention was not paid to whether the study was sufficiently sized to answer a prespecified question–i.e., it may have been an exploratory, hypothesis-generating study.

**Question 6. Exposure assessed prior to outcome measurement**

This question is important because, in order to determine whether an exposure causes an outcome, the exposure must come before the outcome.

For some prospective cohort studies, the investigator enrolls the cohort and then determines the exposure status of various members of the cohort (large epidemiological studies like Framingham used this approach). However, for other cohort studies, the cohort is selected based on its exposure status, as in the example above of depressed diabetic men (the exposure being depression). Other examples include a cohort identified by its exposure to fluoridated drinking water and then compared to a cohort living in an area without fluoridated water, or a cohort of military personnel exposed to combat in the Gulf War compared to a cohort of military personnel not deployed in a combat zone.

With either of these types of cohort studies, the cohort is followed forward in time (i.e., prospectively) to assess the outcomes that occurred in the exposed members compared to nonexposed members of the cohort. Therefore, you begin the study in the present by looking at groups that were exposed (or not) to some biological or behavioral factor, intervention, etc., and then you follow them forward in time to examine outcomes. If a cohort study is conducted properly, the answer to this question should be "yes," since the exposure status of members of the cohort was determined at the beginning of the study before the outcomes occurred.

For retrospective cohort studies, the same principal applies. The difference is that, rather than identifying a cohort in the present and following them forward in time, the investigators go back in time (i.e., retrospectively) and select a cohort based on their exposure status in the past and then follow them forward to assess the outcomes that occurred in the exposed and nonexposed cohort members. Because in retrospective cohort studies the exposure and outcomes may have already occurred (it depends on how long they follow the cohort), it is important to make sure that the exposure preceded the outcome.

Sometimes cross-sectional studies are conducted (or cross-sectional analyses of cohort-study data), where the exposures and outcomes are measured during the same timeframe. As a result, cross-sectional analyses provide weaker evidence than regular cohort studies regarding a potential causal relationship between exposures and outcomes. For cross-sectional analyses, the answer to Question 6 should be "no."

**Question 7. Sufficient timeframe to see an effect**

Did the study allow enough time for a sufficient number of outcomes to occur or be observed, or enough time for an exposure to have a biological effect on an outcome? In the examples given above, if clinical depression has a biological effect on increasing risk for CVD, such an effect may take years. In the other example, if higher dietary sodium increases BP, a short timeframe may be sufficient to assess its association with BP, but a longer timeframe would be needed to examine its association with heart attacks.

The issue of timeframe is important to enable meaningful analysis of the relationships between exposures and outcomes to be conducted. This often requires at least several years, especially when looking at health outcomes, but it depends on the research question and outcomes being examined.

Cross-sectional analyses allow no time to see an effect, since the exposures and outcomes are assessed at the same time, so those would get a "no" response.

**Question 8. Different levels of the exposure of interest**

If the exposure can be defined as a range (examples: drug dosage, amount of physical activity, amount of sodium consumed), were multiple categories of that exposure assessed? (for example, for drugs: not on the medication, on a low dose, medium dose, high dose; for dietary sodium, higher than average U.S. consumption, lower than recommended consumption, between the two). Sometimes discrete categories of exposure are not used, but instead exposures are measured as continuous variables (for example, mg/day of dietary sodium or BP values).

In any case, studying different levels of exposure (where possible) enables investigators to assess trends or dose-response relationships between exposures and outcomes–e.g., the higher the exposure, the greater the rate of the health outcome. The presence of trends or dose-response relationships lends credibility to the hypothesis of causality between exposure and outcome.

For some exposures, however, this question may not be applicable (e.g., the exposure may be a dichotomous variable like living in a rural setting versus an urban setting, or vaccinated/not vaccinated with a one-time vaccine). If there are only two possible exposures (yes/no), then this question should be given an "NA," and it should not count negatively towards the quality rating.

**Question 9. Exposure measures and assessment**

Were the exposure measures defined in detail? Were the tools or methods used to measure exposure accurate and reliable–for example, have they been validated or are they objective? This issue is important as it influences confidence in the reported exposures. When exposures are measured with less accuracy or validity, it is harder to see an association between exposure and outcome even if one exists. Also as important is whether the exposures were assessed in the same manner within groups and between groups; if not, bias may result.

For example, retrospective self-report of dietary salt intake is not as valid and reliable as prospectively using a standardized dietary log plus testing participants' urine for sodium content. Another example is measurement of BP, where there may be quite a difference between usual care, where clinicians measure BP however it is done in their practice setting (which can vary considerably), and use of trained BP assessors using standardized equipment (e.g., the same BP device which has been tested and calibrated) and a standardized protocol (e.g., patient is seated for 5 minutes with feet flat on the floor, BP is taken twice in each arm, and all four measurements are averaged). In each of these cases, the former would get a "no" and the latter a "yes."

Here is a final example that illustrates the point about why it is important to assess exposures consistently across all groups: If people with higher BP (exposed cohort) are seen by their providers more frequently than those without elevated BP (nonexposed group), it also increases the chances of detecting and documenting changes in health outcomes, including CVD-related events. Therefore, it may lead to the conclusion that higher BP leads to more CVD events. This may be true, but it could also be due to the fact that the subjects with higher BP were seen more often; thus, more CVD-related events were detected and documented simply because they had more encounters with the health care system. Thus, it could bias the results and lead to an erroneous conclusion.

**Question 10. Repeated exposure assessment**

Was the exposure for each person measured more than once during the course of the study period? Multiple measurements with the same result increase our confidence that the exposure status was correctly classified. Also, multiple measurements enable investigators to look at changes in exposure over time, for example, people who ate high dietary sodium throughout the followup period, compared to those who started out high then reduced their intake, compared to those who ate low sodium throughout. Once again, this may not be applicable in all cases. In many older studies, exposure was measured only at baseline. However, multiple exposure measurements do result in a stronger study design.

**Question 11. Outcome measures**

Were the outcomes defined in detail? Were the tools or methods for measuring outcomes accurate and reliable–for example, have they been validated or are they objective? This issue is important because it influences confidence in the validity of study results. Also important is whether the outcomes were assessed in the same manner within groups and between groups.

An example of an outcome measure that is objective, accurate, and reliable is death–the outcome measured with more accuracy than any other. But even with a measure as objective as death, there can be differences in the accuracy and reliability of how death was assessed by the investigators. Did they base it on an autopsy report, death certificate, death registry, or report from a family member? Another example is a study of whether dietary fat intake is related to blood cholesterol level (cholesterol level being the outcome), and the cholesterol level is measured from fasting blood samples that are all sent to the same laboratory. These examples would get a "yes." An example of a "no" would be self-report by subjects that they had a heart attack, or self-report of how much they weigh (if body weight is the outcome of interest).

Similar to the example in Question 9, results may be biased if one group (e.g., people with high BP) is seen more frequently than another group (people with normal BP) because more frequent encounters with the health care system increases the chances of outcomes being detected and documented.

**Question 12. Blinding of outcome assessors**

Blinding means that outcome assessors did not know whether the participant was exposed or unexposed. It is also sometimes called "masking." The objective is to look for evidence in the article that the person(s) assessing the outcome(s) for the study (for example, examining medical records to determine the outcomes that occurred in the exposed and comparison groups) is masked to the exposure status of the participant. Sometimes the person measuring the exposure is the same person conducting the outcome assessment. In this case, the outcome assessor would most likely not be blinded to exposure status because they also took measurements of exposures. If so, make a note of that in the comments section.

As you assess this criterion, think about whether it is likely that the person(s) doing the outcome assessment would know (or be able to figure out) the exposure status of the study participants. If the answer is no, then blinding is adequate. An example of adequate blinding of the outcome assessors is to create a separate committee, whose members were not involved in the care of the patient and had no information about the study participants' exposure status. The committee would then be provided with copies of participants' medical records, which had been stripped of any potential exposure information or personally identifiable information. The committee would then review the records for prespecified outcomes according to the study protocol. If blinding was not possible, which is sometimes the case, mark "NA" and explain the potential for bias.

**Question 13. Followup rate**

Higher overall followup rates are always better than lower followup rates, even though higher rates are expected in shorter studies, whereas lower overall followup rates are often seen in studies of longer duration. Usually, an acceptable overall followup rate is considered 80 percent or more of participants whose exposures were measured at baseline. However, this is just a general guideline. For example, a 6-month cohort study examining the relationship between dietary sodium intake and BP level may have over 90 percent followup, but a 20-year cohort study examining effects of sodium intake on stroke may have only a 65 percent followup rate.

**Question 14. Statistical analyses**

Were key potential confounding variables measured and adjusted for, such as by statistical adjustment for baseline differences? Logistic regression or other regression methods are often used to account for the influence of variables not of interest.

This is a key issue in cohort studies, because statistical analyses need to control for potential confounders, in contrast to an RCT, where the randomization process controls for potential confounders. All key factors that may be associated both with the exposure of interest and the outcome–that are not of interest to the research question–should be controlled for in the analyses.

For example, in a study of the relationship between cardiorespiratory fitness and CVD events (heart attacks and strokes), the study should control for age, BP, blood cholesterol, and body weight, because all of these factors are associated both with low fitness and with CVD events. Well-done cohort studies control for multiple potential confounders.

Some general guidance for determining the overall quality rating of observational cohort and cross-sectional studies The questions on the form are designed to help you focus on the key concepts for evaluating the internal validity of a study. They are not intended to create a list that you simply tally up to arrive at a summary judgment of quality.

Internal validity for cohort studies is the extent to which the results reported in the study can truly be attributed to the exposure being evaluated and not to flaws in the design or conduct of the study–in other words, the ability of the study to draw associative conclusions about the effects of the exposures being studied on outcomes. Any such flaws can increase the risk of bias.

Critical appraisal involves considering the risk of potential for selection bias, information bias, measurement bias, or confounding (the mixture of exposures that one cannot tease out from each other). Examples of confounding include co-interventions, differences at baseline in patient characteristics, and other issues throughout the questions above. High risk of bias translates to a rating of poor quality. Low risk of bias translates to a rating of good quality. (Thus, the greater the risk of bias, the lower the quality rating of the study.)

In addition, the more attention in the study design to issues that can help determine whether there is a causal relationship between the exposure and outcome, the higher quality the study. These include exposures occurring prior to outcomes, evaluation of a dose-response gradient, accuracy of measurement of both exposure and outcome, sufficient timeframe to see an effect, and appropriate control for confounding–all concepts reflected in the tool.

Generally, when you evaluate a study, you will not see a "fatal flaw," but you will find some risk of bias. By focusing on the concepts underlying the questions in the quality assessment tool, you should ask yourself about the potential for bias in the study you are critically appraising. For any box where you check "no" you should ask, "What is the potential risk of bias resulting from this flaw in study design or execution?" That is, does this factor cause you to doubt the results that are reported in the study or doubt the ability of the study to accurately assess an association between exposure and outcome?

The best approach is to think about the questions in the tool and how each one tells you something about the potential for bias in a study. The more you familiarize yourself with the key concepts, the more comfortable you will be with critical appraisal. Examples of studies rated good, fair, and poor are useful, but each study must be assessed on its own based on the details that are reported and consideration of the concepts for minimizing bias.


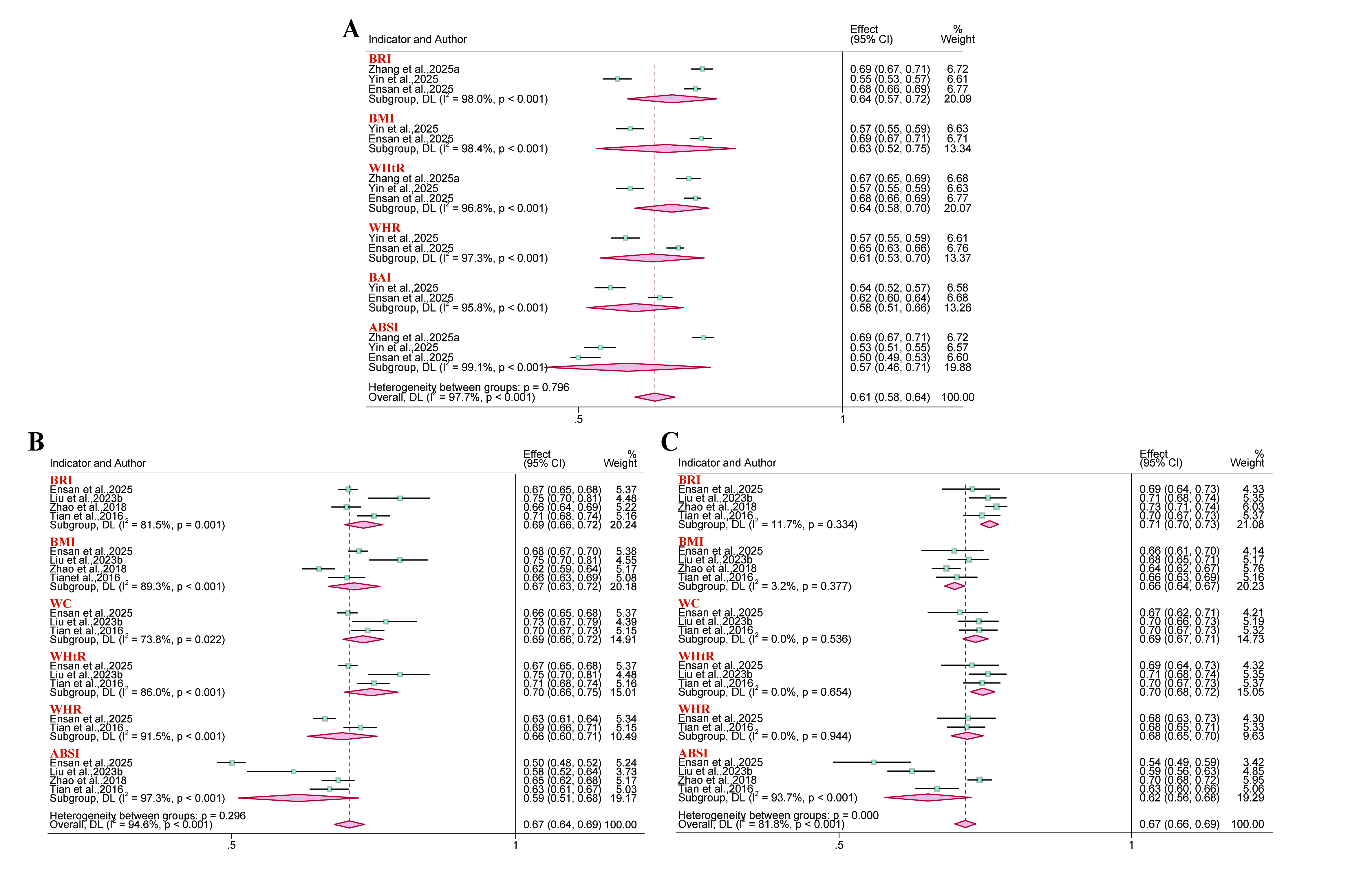


Suppl. Fig. S1. Pooled AUC values of different anthropometric indices for predicting diabetes risk. (A) Overall population; (B) Male population; (C) Female population.


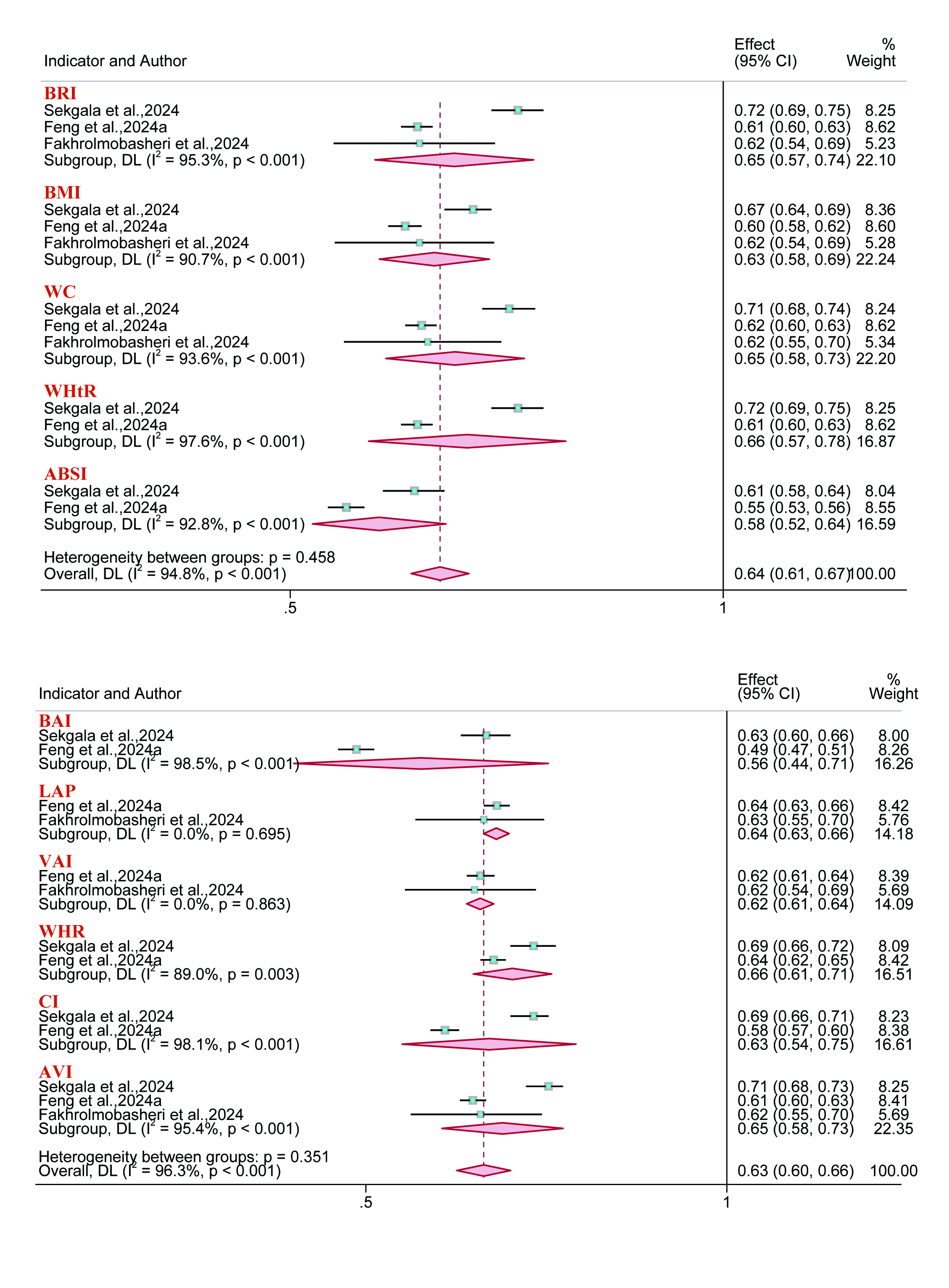


Suppl. Fig. S2. Pooled AUC values of different anthropometric indices for predicting type 2 diabetes risk in the overall population.


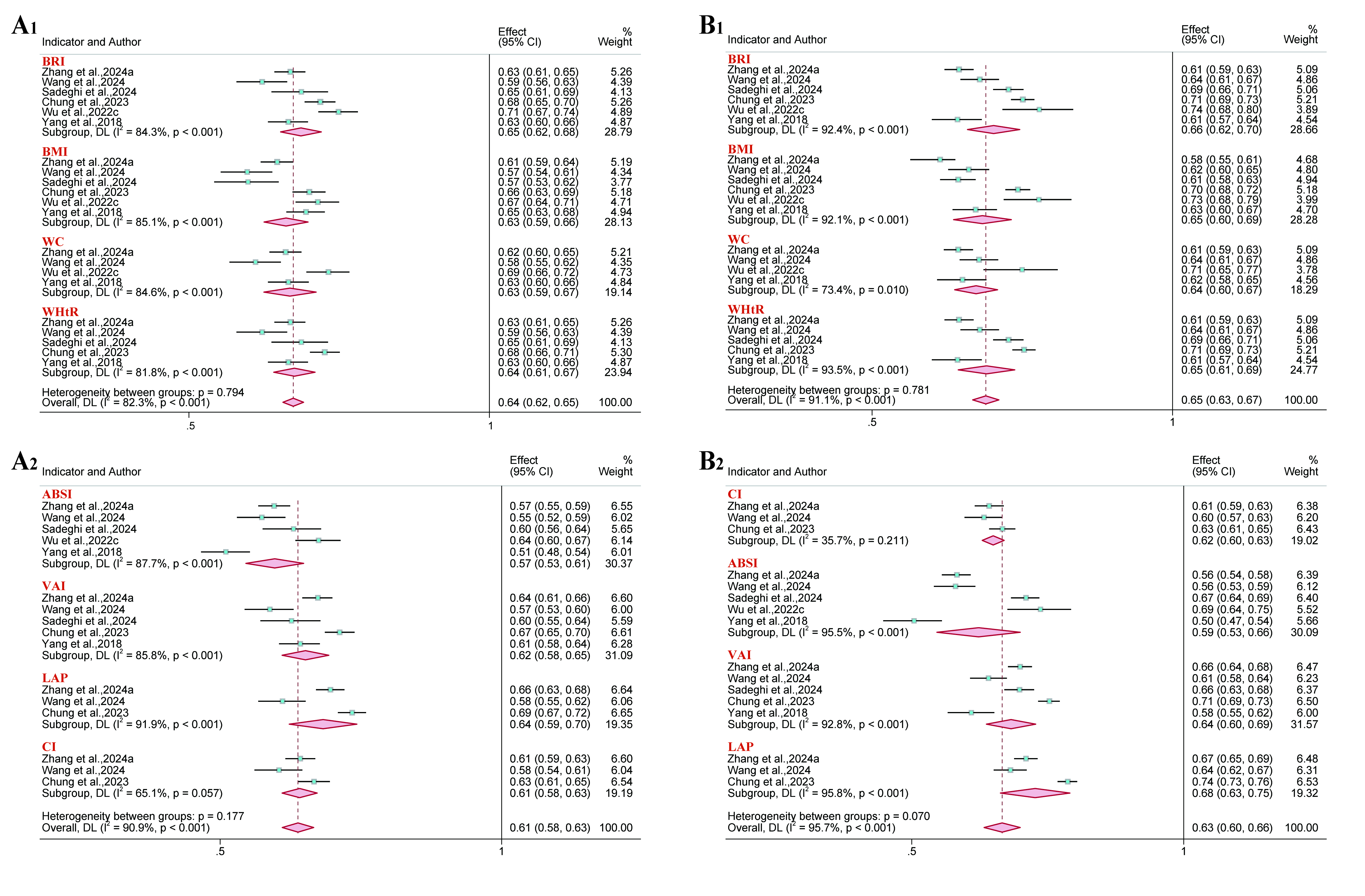


Suppl. Fig. S3. Pooled AUC values of different anthropometric indices for predicting type 2 diabetes risk. (A1-A2) Male population; (B1-B2) Female population.


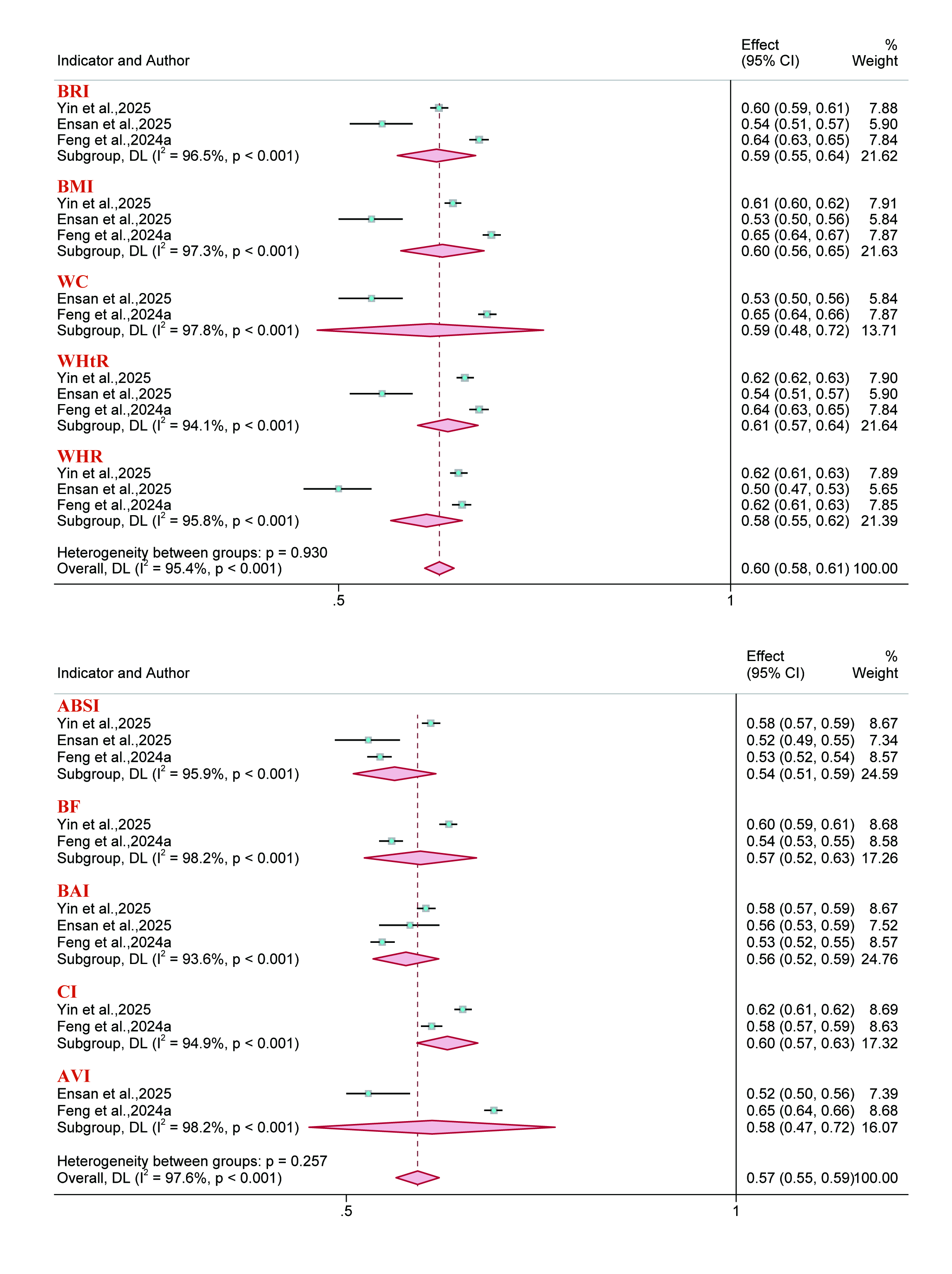


Suppl. Fig. S4. Pooled AUC values of different anthropometric indices for predicting dyslipidemia risk in the overall population.


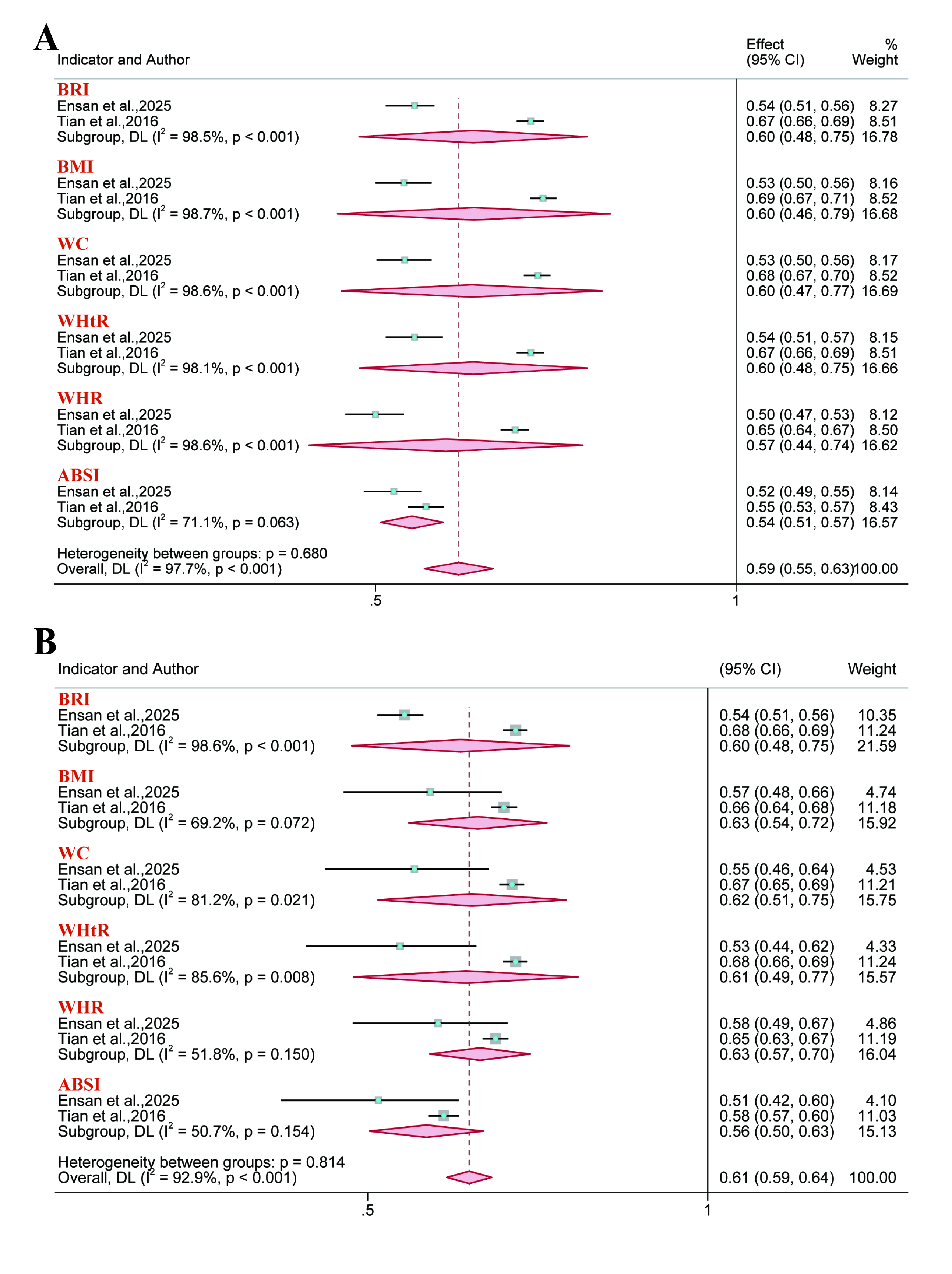


Suppl. Fig. S5. Pooled AUC values of different anthropometric indices for predicting dyslipidemia risk. (A) Male population; (B) Female population.


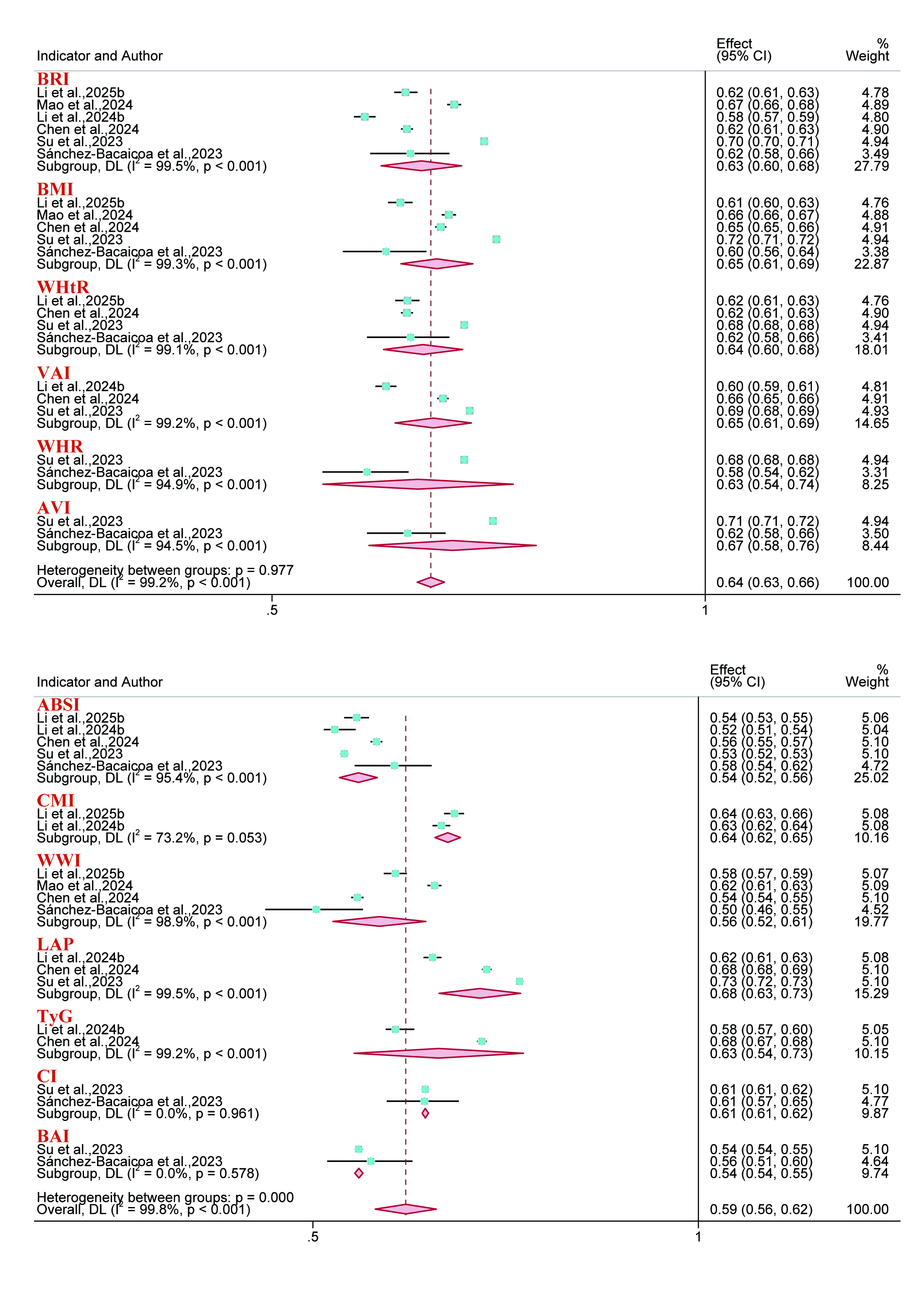


Suppl. Fig. S6. Pooled AUC values of different anthropometric indices for predicting hyperuricemia risk in the overall population.


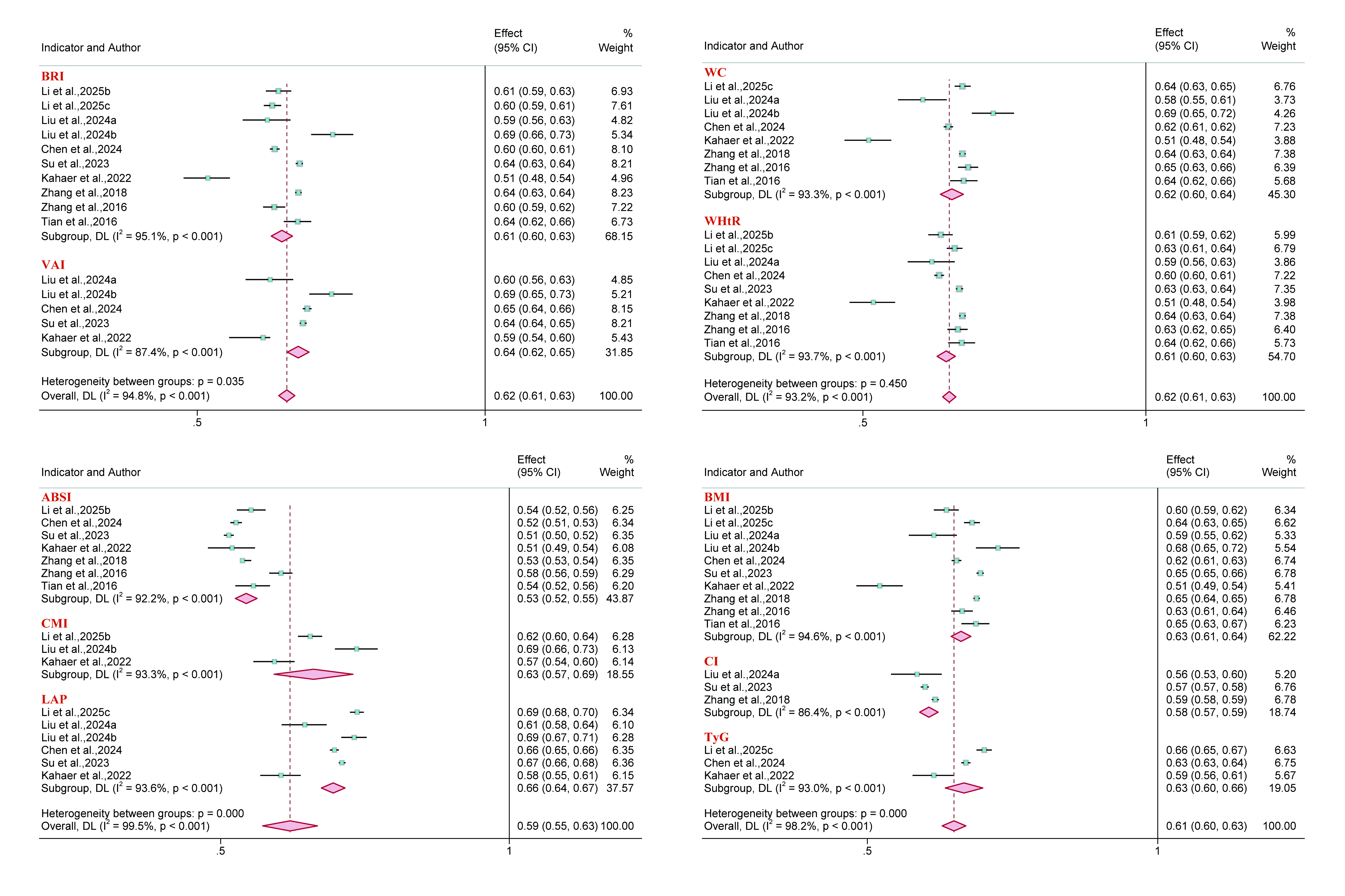


Suppl. Fig. S7. Pooled AUC values of different anthropometric indices for predicting hyperuricemia risk in the male population.


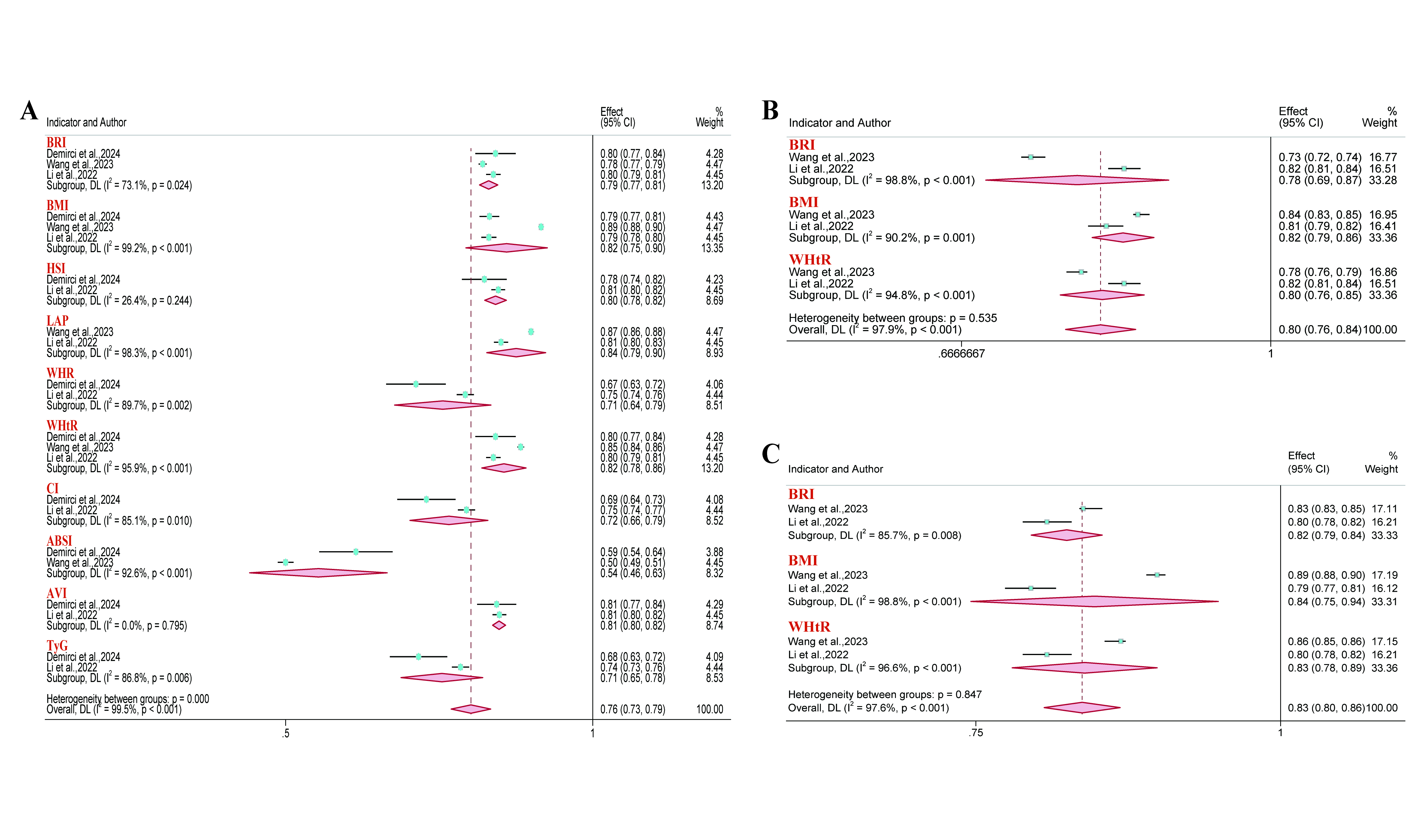


Suppl. Fig. S8. Pooled AUC values of different anthropometric indices for predicting MAFLD risk. (A) Overall population; (B) Male population; (C) Female population.


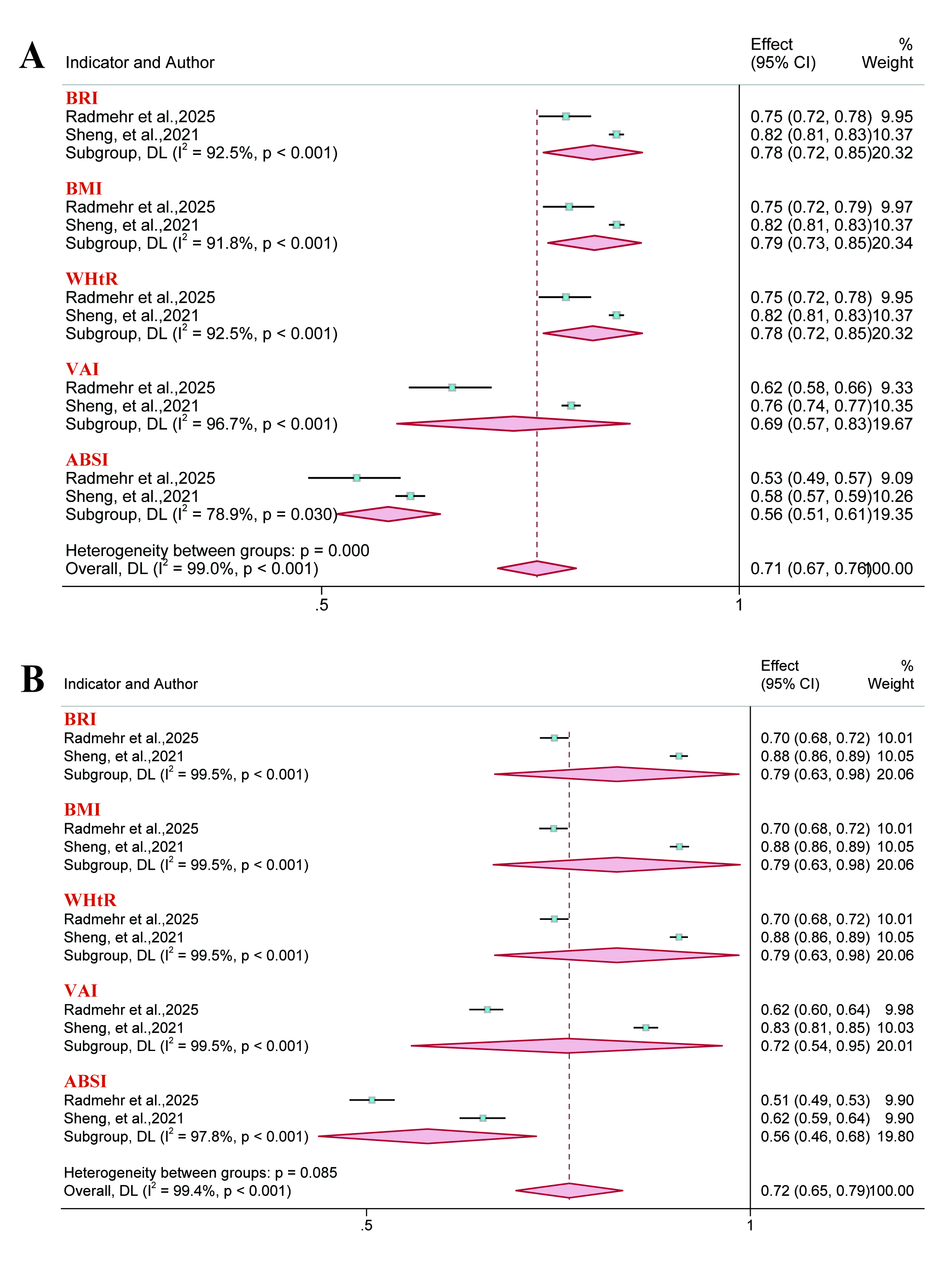


Suppl. Fig. S9. Pooled AUC values of different anthropometric indices for predicting NAFLD risk. (A) Male population; (B) Female population.


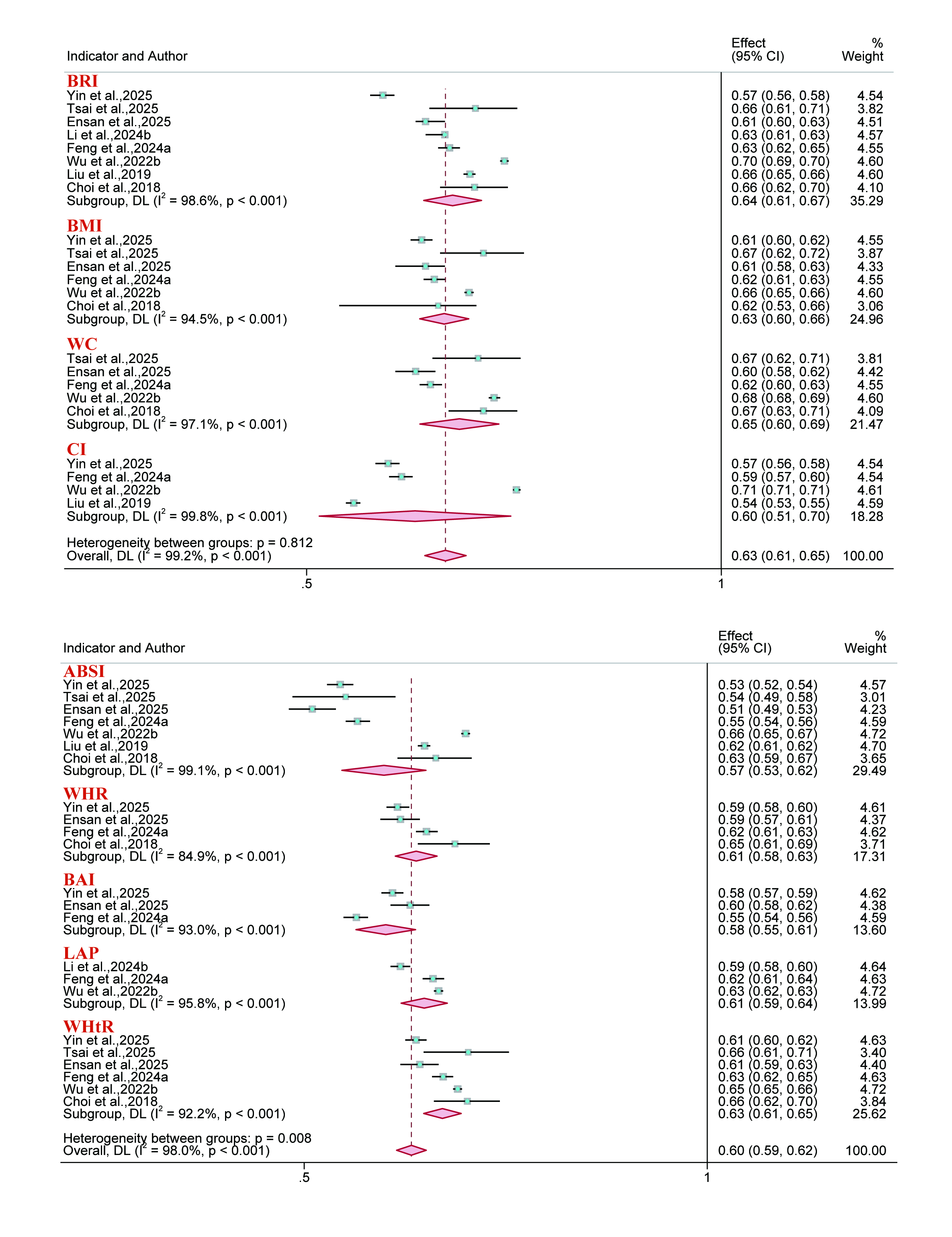


Suppl. Fig. S10. Pooled AUC values of different anthropometric indices for predicting hypertension risk in the overall population.


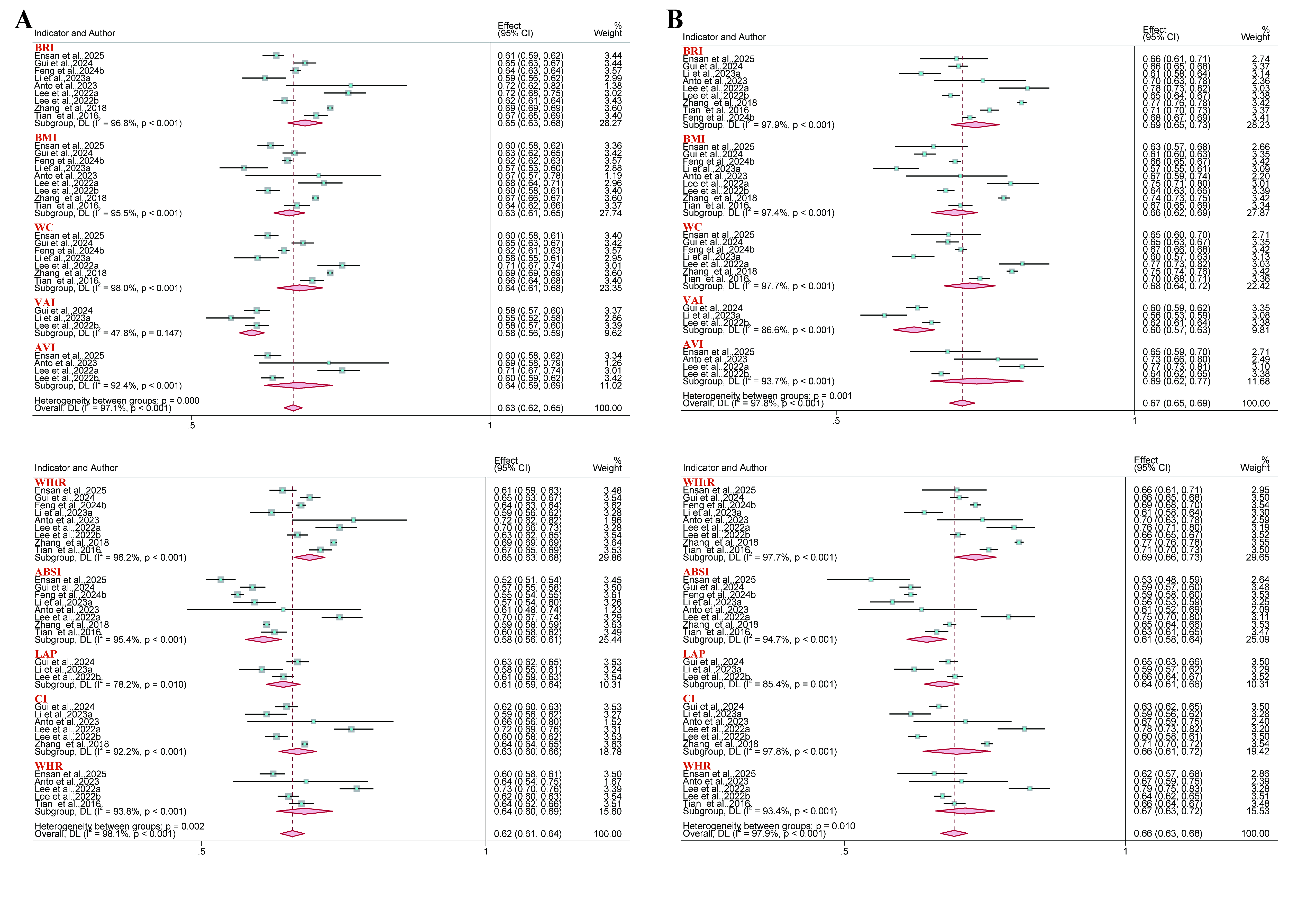


Suppl. Fig. S11. Pooled AUC values of different anthropometric indices for predicting hypertension risk. (A) Male population; (B) Female population.


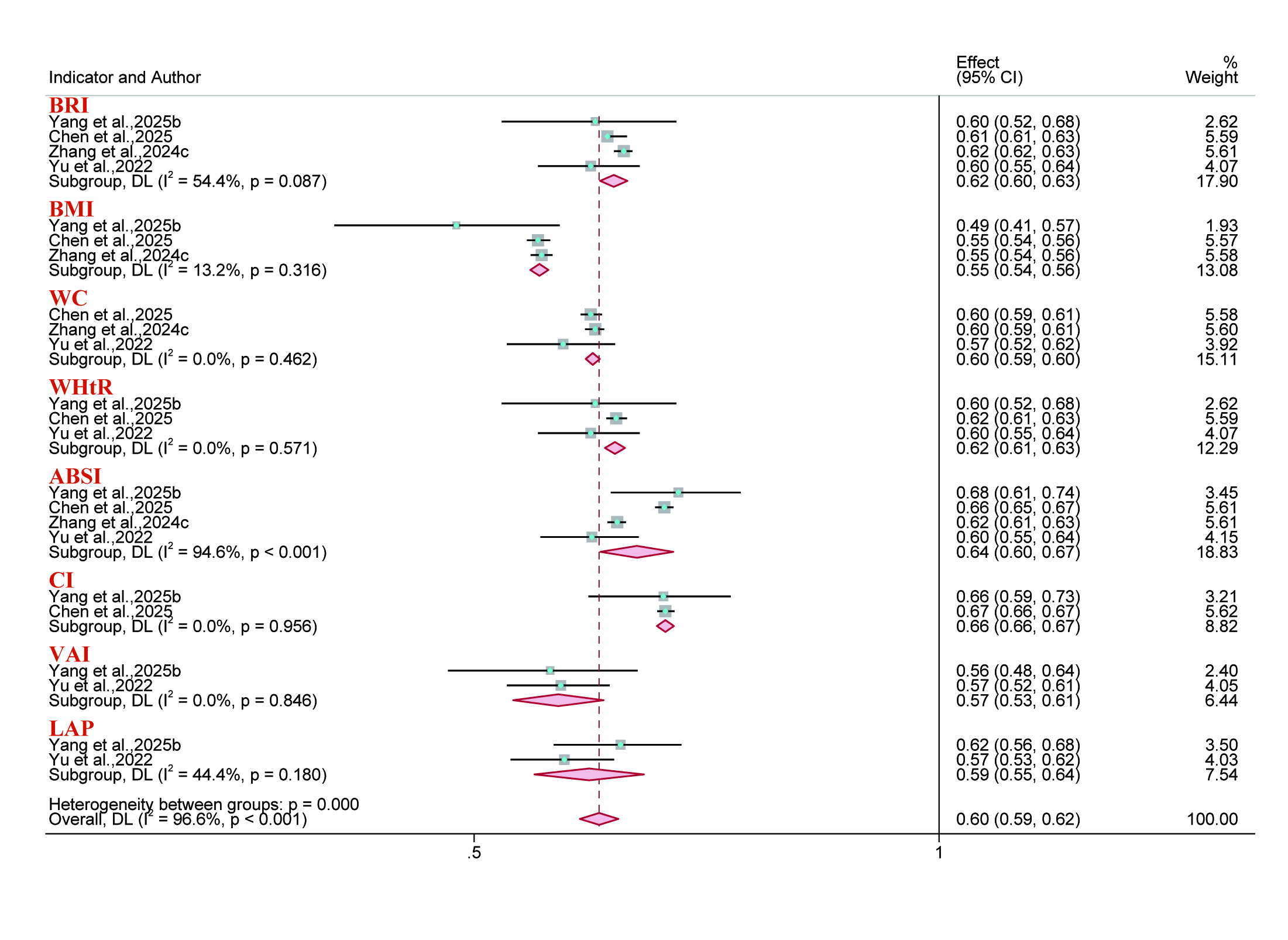


Suppl. Fig. S12. Pooled AUC values of different anthropometric indices for predicting chronic kidney disease risk in the overall population.


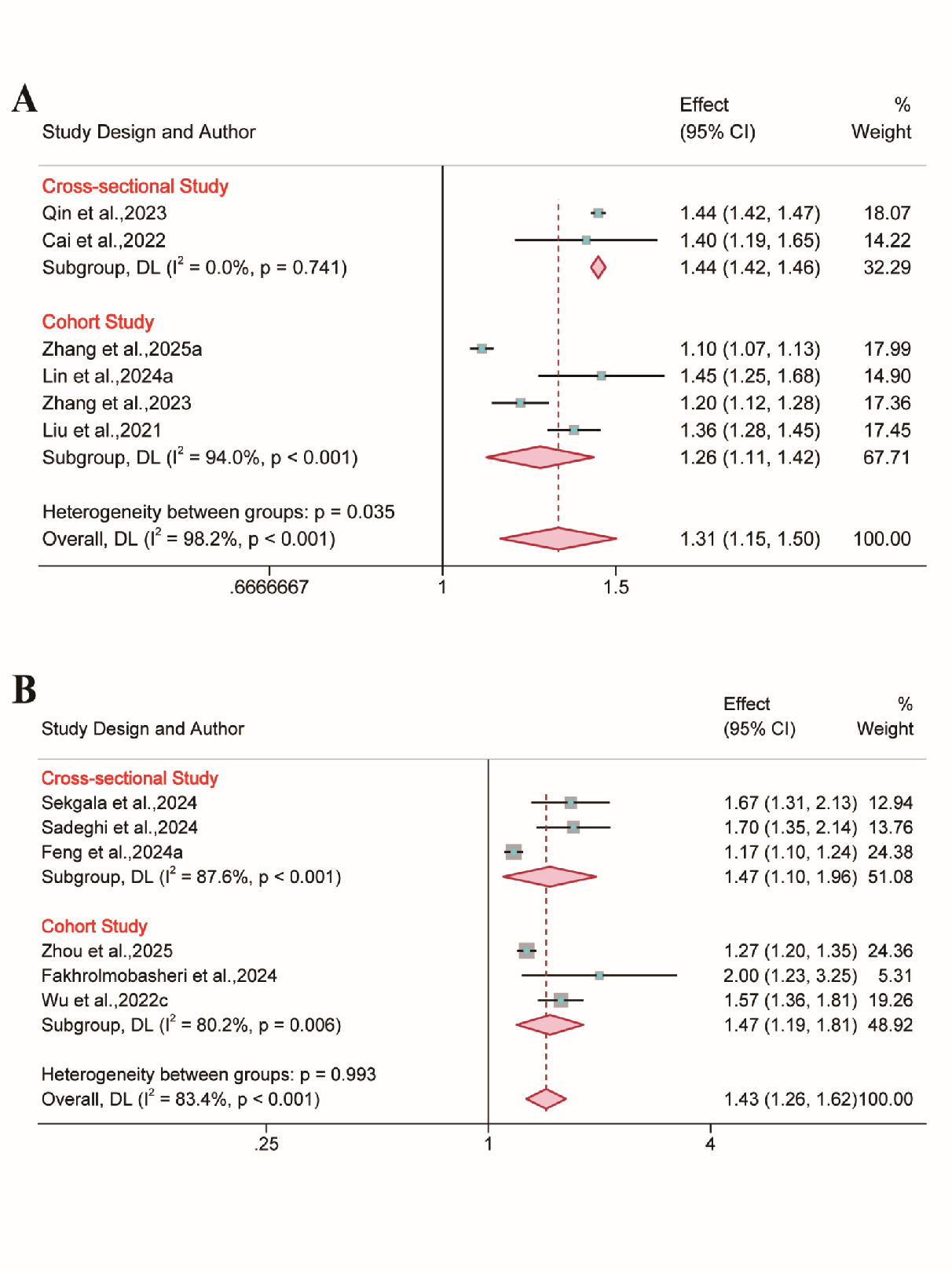


Suppl. Fig. S13. The subgroup analysis by study design. (A) Diabetes Mellitus; (B) Type 2 diabetes.


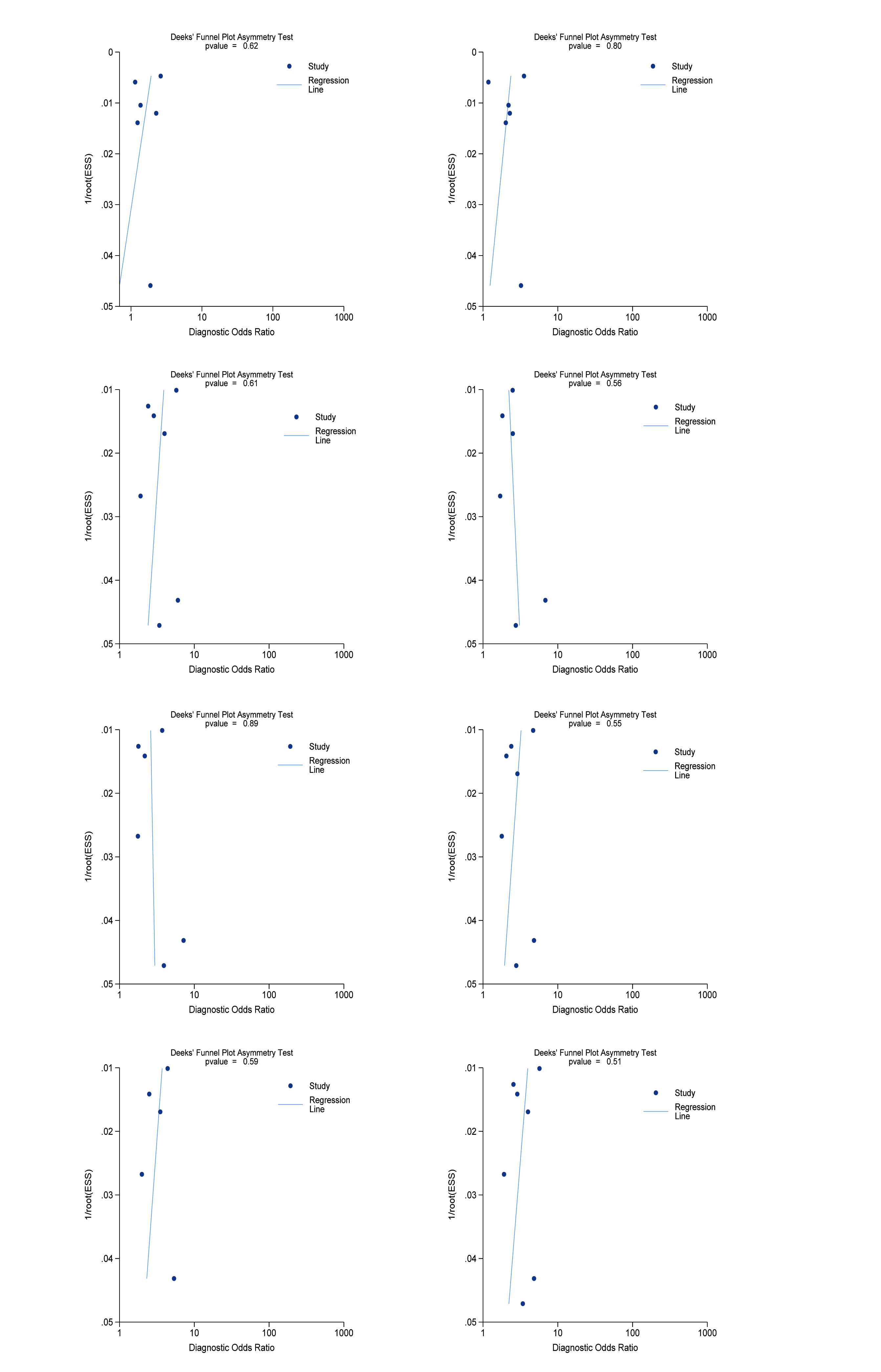


Suppl. Fig. S14. Publication bias assessment for included hypertension studies via Deek’ s Funnel Plot Asymmetry Test.


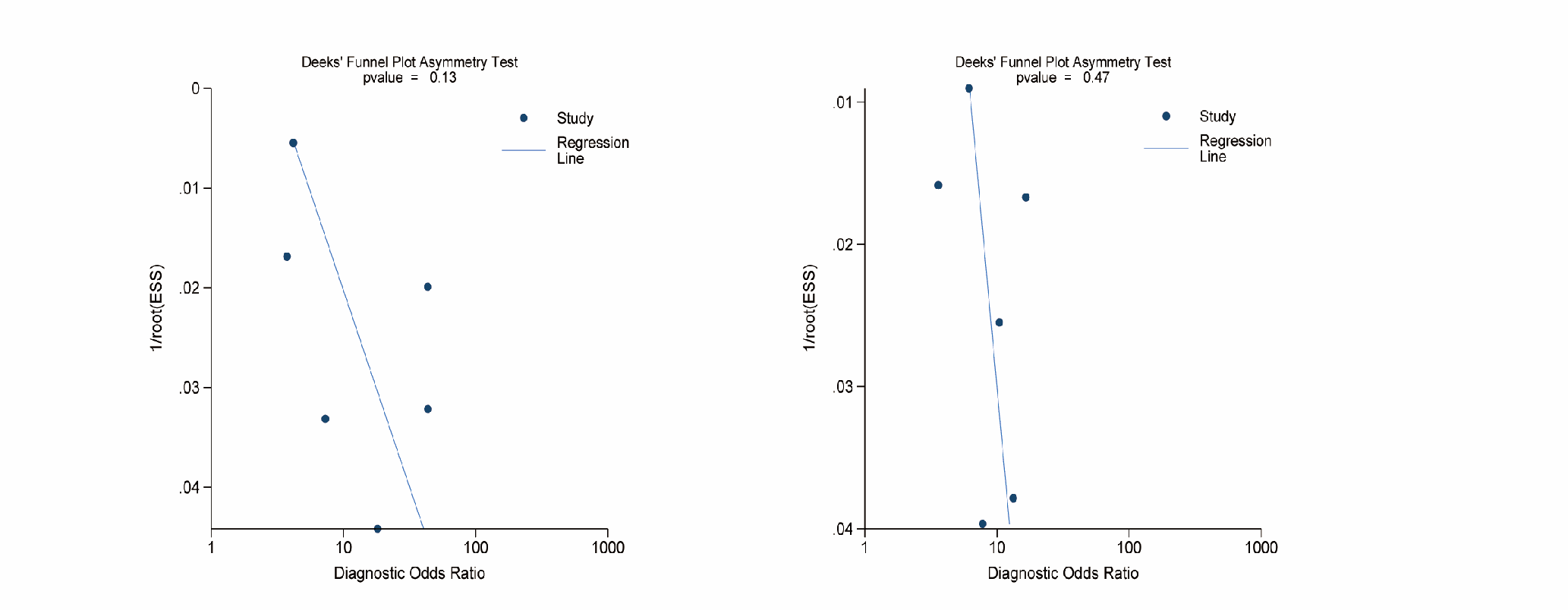


Suppl. Fig. S15. Publication bias assessment for included metabolic syndrome studies via Deek’s Funnel Plot Asymmetry Test.


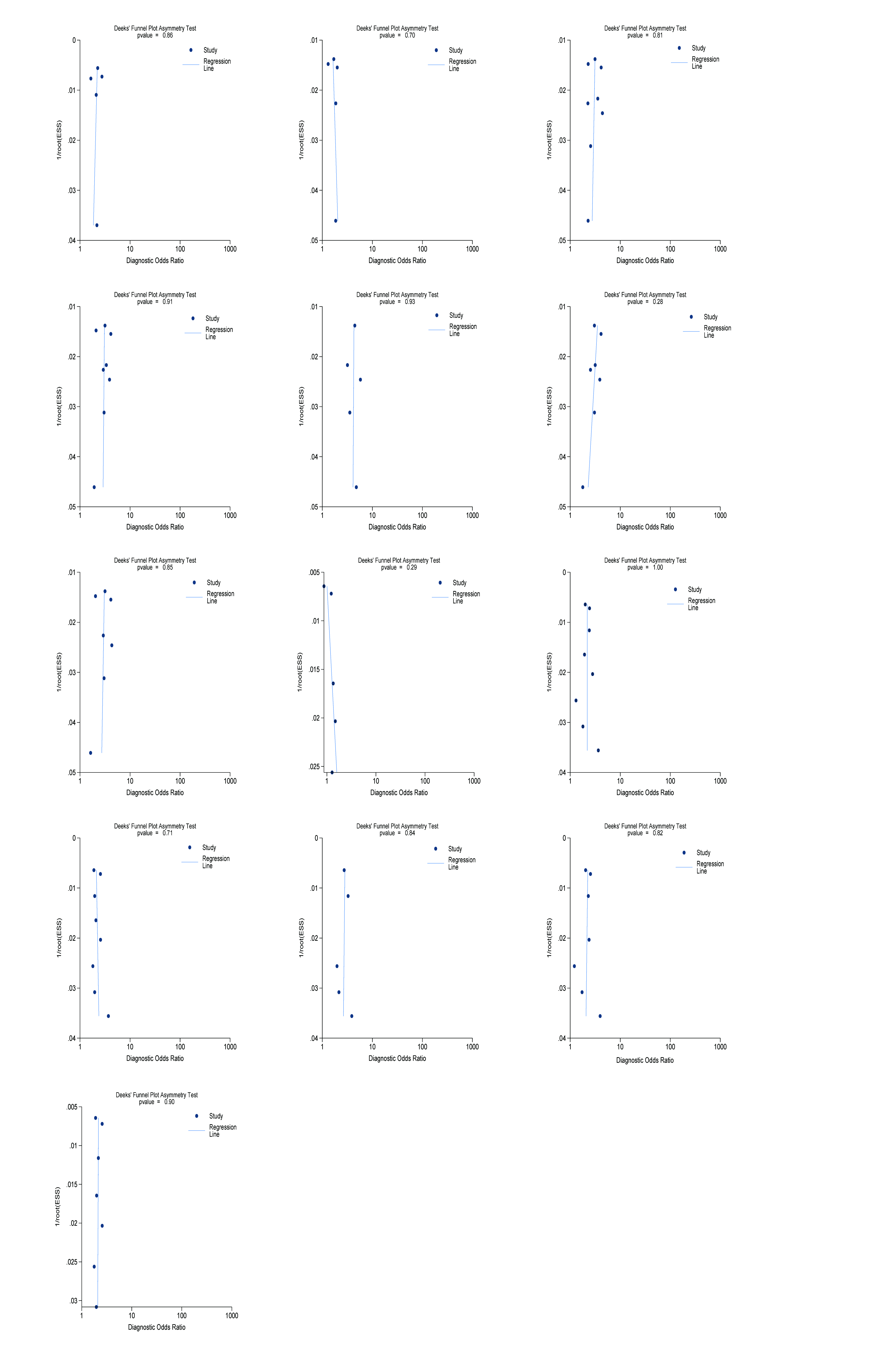


Suppl. Fig. S16. Publication bias assessment for included hyperuricemia studies via Deek’s Funnel Plot Asymmetry Test.
